# Seed protein content and its relationships with agronomic traits in pigeonpea is controlled by both main and epistatic effects QTLs

**DOI:** 10.1038/s41598-019-56903-z

**Published:** 2020-01-14

**Authors:** Jimmy Obala, Rachit K. Saxena, Vikas K. Singh, Sandip M. Kale, Vanika Garg, C. V. Sameer Kumar, K. B. Saxena, Pangirayi Tongoona, Julia Sibiya, Rajeev K. Varshney

**Affiliations:** 1 International Crops Research Institute for the Semi-Arid Tropics (ICRISAT), Hyderabad, 502324 India; 2 University of KwaZulu-Natal, African Center for Crop Improvement, Scottsville, 3209 Pietermaritzburg South Africa; 3 Professor Jayashankar Telangana State Agricultural University, Rajendranagar, Hyderabad, 500030 Telangana India; 4 Present Address: Former Principal Scientist (ICRISAT); 17, NMC Housing, Al Ain, Abu Dhabi UAE

**Keywords:** Genetics, Plant sciences

## Abstract

The genetic architecture of seed protein content (SPC) and its relationships to agronomic traits in pigeonpea is poorly understood. Accordingly, five F_2_ populations segregating for SPC and four agronomic traits (seed weight (SW), seed yield (SY), growth habit (GH) and days to first flowering (DFF)) were phenotyped and genotyped using genotyping-by-sequencing approach. Five high-density population-specific genetic maps were constructed with an average inter-marker distance of 1.6 to 3.5 cM, and subsequently, integrated into a consensus map with average marker spacing of 1.6 cM. Based on analysis of phenotyping data and genotyping data, 192 main effect QTLs (M-QTLs) with phenotypic variation explained (PVE) of 0.7 to 91.3% were detected for the five traits across the five populations. Major effect (PVE ≥ 10%) M-QTLs included 14 M-QTLs for SPC, 16 M-QTLs for SW, 17 M-QTLs for SY, 19 M-QTLs for GH and 24 M-QTLs for DFF. Also, 573 epistatic QTLs (E-QTLs) were detected with PVE ranging from 6.3 to 99.4% across traits and populations. Colocalization of M-QTLs and E-QTLs explained the genetic basis of the significant (P < 0.05) correlations of SPC with SW, SY, DFF and GH. The nature of genetic architecture of SPC and its relationship with agronomic traits suggest that genomics-assisted breeding targeting genome-wide variations would be effective for the simultaneous improvement of SPC and other important traits.

## Introduction

Protein deficiency affects the health of millions of children and their mothers, but protein-rich plant foods may offer solutions particularly in areas of the world where intake of animal protein is low^1^. One such crop is pigeonpea (*Cajanus cajan* (L.) Millsp), which serves as an important source of dietary protein to over one billion people globally^2^. It is widely cultivated in the tropics and semi-arid tropics of Asia and Africa. Pigeonpea maintains better yields than other legume crops under environmental extremes such as heat, drought and low soil fertility conditions^3,4^. These attributes position pigeonpea as the preffered crop for the resources-poor farmers in marginal environments^5^. However, increasing seed protein content (SPC) of pigeonpea is, therefore, an important contribution towards alleviating malnutrition among the poor. Improvement of SPC requires an understanding of its genetic architecture and how it relates to traits of agronomic importance.

Few studies have been reported on genetic control of SPC in pigeonpea with results suggesting quantitative inheritance^6,7^. However, the classical quantitative genetics approaches used in the reported studies are limited in power and resolution to dissect the genetic architecture of a quantitative trait like SPC. Similarly, information is limited on the genetic basis of the often positive or negative or no relationships of SPC with agronomic traits such as seed yield, seed weight, days to flowering, and growth habit in the crop^7,8^. Determining the genetic basis of trait correlations in pigeonpea is essential in designing breeding strategies that aim at improving and stabilizing SPC while maintaining yield and other desirable agronomic attributes. The available genomics, transcriptomics and proteomics resources in pigeonpea coupled with advances in high-throughput genotyping technologies provide opportunity to dissect the genetic architecture of several quantitative traits in the crop^2,9–15^. However, genetic architecture of SPC in pigeonpea and the basis of its relationships with other traits of importance has remained untouched by the genomic revolution in the crop. A common genomics approach to understand the genetic architecture of quantitative traits involves whole genome scans to find quantitative trait loci (QTLs)^16^.

Through QTL analysis, genetic parameters such as number of loci, effect types and sizes and epistasis, which constitute the genetic architecture underlying quantitative phenotypic variation can be estimated^17^. The parameters, however, are commonly population specific^18^. As a result, QTLs identified in a given population may not necessarily be found in another population^19^. Thus, an account of the genetic architecture of a trait based on a single population likely describes only a small proportion of all the loci, their effects, and potential interactions that contribute to the intraspecific phenotypic variation for a trait^17,20^. Similarly, QTL analysis involving multiple traits allows for the decomposition of genetic bases of within trait variations as well as correlations among traits in terms of the signs and magnitudes of QTL effects^21^. To this end, the use of two or more segregating mapping populations with two or more measured traits in a single study have become common^20,22,23^. Regardless of the number of segregating populations, QTL analysis is preceded by the development of appropriate mapping populations and anchoring of markers on a genetic map.

In view of the above, the present study reports on the first attempt to dissect the genetic architecture of SPC in pigeonpea in a manner that incorporates an investigation of the genetic basis of its correlations with other important agronomic traits. To achieve this, five segregating F_2_ populations were phenotyped for SPC and agronomic traits including seed yield (SY), 100-seed weight (SW), days to first flowering (DFF) and growth habit (GH). These populations were genotyped using genotyping-by-sequencing (GBS) approach. Five SNPs-based population-specific, and a consensus genetic maps were constructed. Analysis of both main effect QTLs (M-QTL_S_) and epistatic QTLs (E-QTLs) revealed the genetic architecture of SPC variation and the basis of its correlations with the measured agronomic traits.

## Results

### Phenotypic variation in SPC and agronomic traits in five mapping populations

The mean value of SPC in low protein containing parents ranged from 19.3 to 21.5% and from 22.3 to 24.6% among high protein containing parents (Table 1). The lowest SPC difference (0.8%) was observed between crossing parents of Pop2 and highest (3.1%) was in crossing parents of Pop5. In the case of F_2_s, SPC ranged from 5.8% in Pop5 to 10.3% (Pop3) while mean SPC ranged from 19.44 ± 1.28% in Pop4 to 23.06 ± 1.08% (Pop5). Similar statistics for DFF, SW and SY have been presented (Table 1). Shapiro-Wilk test showed that distributions for SPC in Pop1, Pop3 and Pop5 were not significantly (P > 0.05) different from a Gaussian distribution while Pop2 and Pop4 differed significantly (P ≤ 0.05) from a normal distribution. Such non-Gaussian distributions were also noted for most of the other traits such as DFF in Pop1, Pop2, Pop3 and Pop4; SW (Pop2 and Pop5) and SY in all five populations.

**Table 1:** Population size, mean, variance, skewness, kurtosis, minimum and maximum values, and w-test for seed protein content (SPC), days to first flowering (DFF), 100-seed weight (SW) and seed yield (SY) in five F_2_ mapping populations of pigeonpea.

Trait	P_1_	P_2_	|P_1_-P_2_|	n	Mean ± s.d.	CV (%)	S	K	F_2_-range	W-test
**Pop1 (ICP 11605 × ICP 14209)**										
SPC (%)	21.5	23.1	1.6	178	22.2 ± 1.2	5.6	0.2	1.1	19.1–26.5	1.0^NS^
DFF (days)	66.0	138.0	72.0	178	101.1 ± 12.6	12.4	−0.4	0.1	69.0–133.0	0.9***
SW (g)	12.2	8.7	3.5	178	9.1 ± 1.1	12.0	0.0	0.1	6.2–12.2	1.0^NS^
SY (g/plant)	22.5	17.3	5.2	178	53.4 ± 32.5	60.8	1.1	1.6	8.7–186.9	0.9***
**Pop2 (ICP 8863 × ICP 11605)**										
SPC (%)	22.3	21.5	0.8	175	21.7 ± 1.5	6.9	−0.5	−0.2	17.5–24.8	1.0**
DFF (days)	90.0	66.0	24.0	175	83.5 ± 11.1	13.3	0.4	0.5	58.0–117.0	1.0***
SW (g)	9.9	12.2	2.3	175	11.3 ± 1.4	12.4	1.4	9.8	7.5–20.6	0.9***
SY (g/plant)	24.5	22.5	2.0	175	37.3 ± 27.6	73.9	1.5	1.7	8.0–127.5	0.8***
**Pop3 (HPL 24 × ICP 11605)**										
SPC (%)	23.0	21.5	1.5	157	22.4 ± 1.7	7.5	0.3	0.5	17.7–28.0	1.0^NS^
DFF (days)	112.0	66.0	46.0	157	93.4 ± 15.2	16.3	−0.6	−0.8	66.0–123.0	0.9***
SW (g)	8.1	12.2	4.1	157	10.4 ± 1.3	12.8	−0.3	0.2	5.7–13.7	1.0^NS^
SY (g/plant)	152.7	22.5	130.2	157	33.6 ± 21.6	64.4	1.1	0.8	5.7–106.5	0.9***
**Pop4 (ICP 8863 × ICPL 87119)**										
SPC (%)	22.3	19.3	3.0	137	19.4 ± 1.3	6.6	−0.4	−0.3	16.0–21.8	1.0*
DFF (days)	90.0	103.0	13.0	137	95.5 ± 8.7	9.1	−0.4	0.5	62.0–116.0	1.0^NS^
SW (g)	9.9	11.1	1.2	137	11.6 ± 1.1	9.1	−0.3	0.5	8.6–14.1	1.0^NS^
SY (g/plant)	24.5	38.9	14.4	137	50.9 ± 32.6	64.0	1.8	3.9	7.9–192	0.8***
**Pop5 (ICP 5529 × ICP 11605)**										
SPC (%)	24.6	21.5	3.1	179	23.0 ± 1.1	4.7	0.0	0.2	20.2–26.6	1.0 ns
DFF (days)	104.0	66.0	38.0	179	81.2 ± 9.2	11.3	−0.2	−0.5	65.0–102.0	0.9***
SW (g)	8.6	12.2	3.6	179	10.3 ± 1.4	13.1	−0.6	1.2	5.3–13.4	1.0*
SY (g/plant)	23.3	22.5	0.8	179	47.8 ± 38.7	80.9	1.8	3.5	5.3–203.1	0.8***

NS: not significantly different from a Gaussian distribution at P = 0.05; *, ** and ***: significantly different from a Gaussian distribution at 0.05, 0.01 and 0.001 probability levels, respectively. P_1_: parent 1, P_2_: parent 2, |P_1_-P_2_|: absolute difference in trait value between two parents of a cross, S: skewness, K: kurtosis.

### Phenotypic correlation among traits

Correlations among traits are presented in Table 2. Correlations were negative between SPC and DFF in all mapping populations but significant (P ≤ 0.05) in only two populations (Pop1 and Pop3). Similarly, correlations between SPC and SY were negative and significant in all populations except in Pop4. In contrast, significant positive correlations were noted between SPC and GH in three of the five populations. While correlations between SPC and SW were positive in all populations except Pop3, although only significant in two populations (Pop1 and Pop2). Correlations between agronomic traits were generally negative and significant for DFF × GH and SY × GH, positive and nonsignificant for SW × GH and DFF × SY, and negative and nonsignificant for SY × SW.

**Table 2:** Correlation coefficient among traits in five F_2_ mapping populations of pigeonpea.

Traits	SPC	DFF	GH	SW	SPC	DFF	GH	SW
**Pop1 (ICP11605** × **ICP 14209)**					**Pop2 (ICP 8863** × **ICP 11605)**			
DFF	−0.1676				−0.1058			
	(0.0254)				(0.1635)			
GH	0.2004	−0.6891			0.1841	−0.5564		
	(0.0073)	(0.0000)			(0.0147)	(0.0000)		
SW	0.2249	0.0412	0.1396		0.3013	−0.0626	0.0532	
	(0.0025)	(0.5847)	(0.0631)		(0.0001)	(0.4102)	(0.4842)	
SY	−0.1810	0.1543	−0.2381	−0.1074	−0.2303	0.0890	−0.2520	−0.0293
	(0.0156)	(0.0398)	(0.0014)	(0.1536)	(0.0022)	(0.2413)	(0.0008)	(0.7001)
**Pop3 (HPL 24** × **ICP 11605)**					**Pop4 (ICP 8863** × **ICPL 87119)**			
DFF	−0.3046				−0.0009			
	(0.0001)				(0.9920)			
GH	0.3380	−0.7446			—	—		
	(0.0000)	(0.0000)			—	—		
SW	0.1337	0.0023	0.1492		0.0444	−0.1784	—	
	(0.0951)	(0.9770)	(0.0621)		(0.6074)	(0.0377)	—	
SY	−0.2811	0.1419	−0.2874	0.0172	−0.0643	−0.1456	—	0.1803
	(0.0004)	(0.0763)	(0.0003)	(0.8304)	(0.4568)	(0.0909)	—	(0.0357)
**Pop5 (ICP 5529** × **ICP 11606)**					**Combined across populations**			
DFF	−0.0047				−0.2111			
	(0.9542)				(0.0000)			
GH	0.1565	−0.5905			0.2102	−0.5574		
	(0.0518)	(0.0000)			(0.0000)	(0.0000)		
SW	−0.0245	−0.1860	0.1138		−0.1305	−0.1813	0.1198	
	(0.7620)	(0.0205)	(0.1586)		(0.0002)	(0.0000)	(0.0020)	
SY	−0.2392	0.2463	−0.2804	0.1551	−0.1955	0.1586	−0.2608	−0.0196
	(0.0027)	(0.0020)	(0.0004)	(0.0540)	(0.0000)	(0.0000)	(0.0000)	(0.5795)

P values are in parentheses.

### Sequence data and SNP discovery

In total, 403.66 million reads (40.77 Gb), 343.26 million reads (34.76 Gb), 339.25 million reads (33.89 Gb), 284.77 million reads (28.76 Gb) and 298.56 million reads (30.15 Gb) of clean GBS reads were generated using HiSeq. 2500 platform from parents and 178 F_2_s (Pop1), 175 F_2_s (Pop2), 157 F_2_s (Pop3), 137 F_2_s (Pop4) and 179 F_2_s (Pop5), respectively (Table 3; Suplementary Table S1). It is important to mention that sequence data generated and SNPs identified in Pop5 have been taken from Saxena *et al*.^12^. The reads from individual progenies ranged from 0.79 to 5.82 million reads in Pop1, 0.49 to 9.52 million reads in Pop2, 0.73 to 6.84 million reads in Pop3, 0.84 to 8.19 million reads in Pop4, and 0.41 to 5.26 million reads in Pop5. Also, a total of 1.13 (ICP 11605) and 2.60 (ICP 14209) million reads of Pop1 parents, 3.00 (ICP 8863) and 7.59 (ICP 11605) million reads of Pop2 parents, 2.96 (HPL 24) and 3.31 (ICP 11605) million reads of Pop3 parents, 2.56 (ICP 8863) million reads of Pop4 parent, and 5.37 (ICP 5529) and 1.61 (ICP 11605) million reads of Pop5 parents, were generated. The final number of good quality SNPs produced were 15,728 in Pop1, 7,494 in Pop2, 12,030 in Pop3, 11,526 in Pop4 and 12,654 in Pop5^12^ (Table 4).

**Table 3:** Number of reads and data size in gigabytes (Gb) generated in five F_2_ mapping populations of pigeonpea. P_1_, parent 1_;_ P_2_, parent 2; Pop1, ICP 11605 (P_1_) × ICP 14209 (P_2_); Pop2, ICP 8863 (P_1_) × ICP 11605 (P_2_); Pop3, HPL 24 (P_1_) × ICP 11605 (P_2_); Pop4, ICP 8863 (P_1_) × ICPL 87119 (P_2_); Pop5, ICP 5529 (P_1_) × ICP 11605 (P_2_); n, F_2_ population size; ^†^Information obtained from Saxena *et al*.^12^.

Data features/generation	Pop1 (n = 178)	Pop2 (n = 175)	Pop3 (n = 157)	Pop4 (n = 137)	Pop5^†^ (n = 179)
**Number of reads (millions)**					
Total	403.66	343.26	339.25	284.77	298.56
P_1_	1.13	3.0	2.96	2.56	5.37
P_2_	2.6	7.6	3.31	—	1.61
F_2_ - range	0.79–5.82	0.49–9.52	0.73–6.84	0.84–8.19	0.41–5.26
F_2_ - average	2.25	1.9	2.09	2.06	1.67
**Data size (Gb)**					
Total	40.77	34.76	33.89	28.76	30.15
P_1_	0.114	0.303	0.299	0.258	0.543
P_2_	0.263	0.766	0.335	—	0.163
F_2_ - range	0.079–0.587	0.049–0.962	0.074–0.691	0.084–0.827	0.041–0.531
F_2_ - average	0.267	0.192	0.212	0.208	0.168

**Table 4:** Features of individual genetic maps from five F_2_ mapping populations of pigeonpea Pop1: ICP 11605 × ICP 14209, Pop2: ICP 8863 × ICP 11605, Pop3: HPL 24 × ICP 11605, Pop4: ICP8863 × ICPL 87119, Pop5: ICP 5529 × ICP 11605, ^†^Information obtained from Saxena *et al*.^12^.

Features	Individual genetic maps				
	Pop1	Pop2	Pop3	Pop4	Pop5^†^
No. of total SNPs	15728	7494	12030	11526	12654
No. of SNPs showing severe segregation distortion (P < 1.0 × 10^–9^)	12121	6075	9129	7585	9727
No. of markers segregating at 1:2:1 at P ≥ 1.0 × 10^−9^	3607	1419	2901	3941	2935
No. of markers in anchor maps	82	90	94	29	140
Length of anchor maps	561.9	696.2	578.2	374.5	584.2
No. of total mapped loci	662	363	607	996	787
- Mapped non-distorted loci	160	132	178	182	262
- Mapped distorted loci	502	248	517	814	525
Total map length (cM)	1419.1	1327.6	1546.8	1599.8	1454.0
Average marker spacing (cM)	2.1	3.5	2.3	1.6	1.8
Number of gaps > 10.0 cM	13	33	29	15	21
Largest gap (cM)	22.3	40.0	26.0	29.0	25.4

### Population specific genetic maps

From a total of 15,728, 7,494, 12,030, 11,526 and 12,662 SNPs identified, 3,607, 1,419 2,901, 3,941, and 2,935 SNPs in Pop1, Pop2, Pop3, Pop4 and Pop5 (genetic map information for Pop5 has been taken from Saxena *et al*.^12^), respectively, which segregated in 1:2:1 F_2_ genotypic ratio at a χ^2^ cutoff P ≥ 10^−9^ were retained for genetic mapping (Table 4). Owing to high distortion from the expected F_2_ segregation ratio, SNPs segregating in a 1:2:1 ratio at P > 0.05 were used as anchor markers for initial genetic map construction. As a result, a total of 82, 90, 94, 29 and 140 SNPs in Pop1, Pop2, Pop3, Pop4 and Pop5, respectively could be mapped in the base or anchor genetic maps. A further 580, 273, 513, 967 and 647 SNPs, which segregated in 1:2:1 ratio at P < 0.05 ≥ 10^−9^ could be added to the base map resulting in 662, 363, 607, 996 and 787 SNPs mapped, with map lengths of 1419.1 cM, 1327.6 cM, 1546.8 cM, 1599.8 cM and 1454.0 cM in Pop1, Pop2, Pop3, Pop4 and Pop5, respectively. The average marker spacing in cM among the five genetic maps were 2.1 (Pop1), 3.5 (Pop2), 2.3 (Pop3), 1.6 (Pop4) and 1.8 (Pop5). The number of gaps larger than 10.0 cM ranged from 13 in Pop1 to 33 (Pop2). The largest gaps on the genetic maps ranged from 22.3 cM in Pop1 to 40 cM in Pop2 (Table 4, Supplementary Figs. S1–S5).

### Consensus genetic map

All markers used in the construction of the consensus map in the present study were SNPs. As a result, there was no discrepancy in marker names among the individual maps. Segregation data for 3,400 markers from five mapping populations were used to integrate the multiple genetic maps into a consensus map (Table 5). Among the markers, 2,386 were distinctive to particular mapping populations, 617 were common between two, 227 among three and 170 among four mapping populations. The common markers were used as anchor points for integration of the individual genetic maps. Most of the linkage groups for the population-specific genetic maps were integrated into the consensus genetic map. All common markers together led to the production of a consensus genetic map comprising 984 loci on 11 CcLGs covering a map distance of 1,609.5 cM with an average inter-marker distance of 1.6 cM (Supplementary Fig. S7).

**Table 5:** Number of common markers among five F_2_ mapping populations of pigeonpea. Pop1: ICP 11605 × ICP 14209, Pop2: ICP 8863 × ICP 11605, Pop3: HPL 24 × ICP 11605, Pop4: ICP8863 × ICPL 87119, Pop5: ICP 5529 × ICP 11605. n, number of markers.

Population	Size	Total	Number of markers common to ‘n’ mapping pop				Total common	
			n = 0	n = 1	n = 2	n = 3	Number	%
Pop1	178	647	413	141	50	43	234	36.2
Pop2	175	363	170	107	44	42	193	53.2
Pop3	157	607	356	150	58	43	251	41.4
Pop4	137	996	915	62	17	2	81	8.1
Pop5	179	787	532	157	58	40	255	32.4
Total	826	3,400	2,386	617	227	170	1,014	29.8

### Collinearity between component and consensus genetic maps

**Figure 1:**
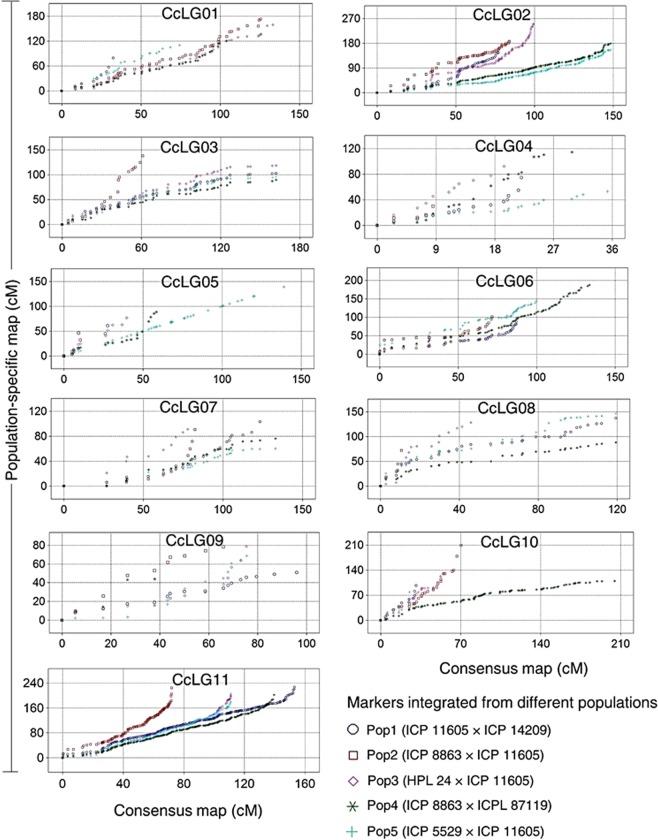
Scatter plots showing the extent of correlations among population-specific and consensus genetic maps of pigeonpea.

**Figure 2:**
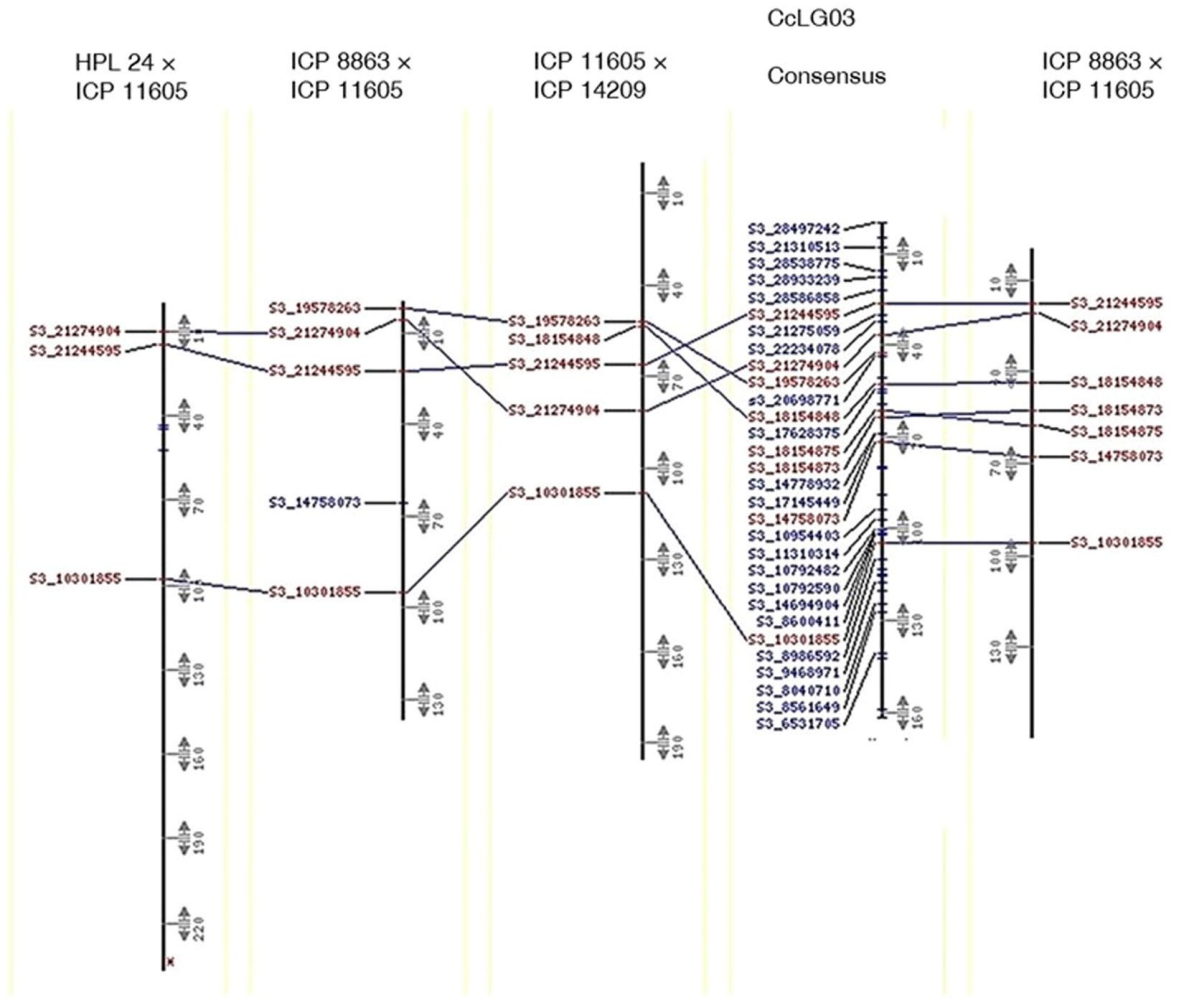
A chart depicting marker-based correspondences of consensus with individual genetic maps, a case of CcLG03. Only common markers are included to visually assess the collinearity of marker orders and marker positions. Linkage groups are aligned together using comparative mapping programme CMap version 1.01. This figure and for all the other linkage groups are presented as Supplementary Fig. S6.

All genetic maps were, to a large extent, collinear with the consensus map (Table 6; Figs. 1 and 2; Supplementary Fig. S6). However, component CcLGs from Pop1 (CcLG02, CcLG07, CcLG08 and CcLG11), Pop2 (CcLG07), Pop3 (CcLG08 and CcLG11) and Pop5 (CcLG05, CcLG07and CcLG09) showed a reversal of marker order between component genetic map and consensus map as revealed by the negative correlation coefficients (“*r*”; Table 6). Similarly, CcLGs from Pop4 that contributed any markers to the consensus map displayed poor collinearity with the consensus map. Finally, genome-wide, there were 13 gaps larger than 10 cM (one each on CcLG02 and CcLG11, two each on CcLG05, CcLG09 and CcLG10, and three each on CcLG03 and CcLG07). Such gaps have been thought to result from recombination hotspots or regions that are identical-by-descent and thus lack of polymorphisms^24^.

**Table 6:** Summary of a pigeonpea consensus genetic map constructed from five component genetic maps.

Consensus map				Number of markers contributed from component genetic maps and their correlation with consensus map									
				Pop1		Pop2		Pop3		Pop4		Pop5	
LG^†^	N	ML (cM)	AID (cM)	N	“*r*”	n	“*r*”	n	“*r*”	n	“*r*”	n	“*r*”
CcLG01	52	136.8	2.6	—	—	11	0.97***	13	0.81***	1	—	20	0.91***
CcLG02	219	224.3	1.0	9	−0.80**	12	0.97***	13	0.99***	172	0.23**	30	0.95***
CcLG03	46	162.0	3.5	25	0.95***	15	0.97***	13	0.97***	—	—	22	0.95**
CcLG04	29	49.6	1.7	11	0.89***	4	0.99***	3	1.00*	—	—	18	0.57*
CcLG05	24	140.1	5.8	7	1.00***	4	0.99**	10	1.00***	—	—	13	−0.87***
CcLG06	76	139.6	1.8	36	0.34*	23	0.98***	27	0.95***	—	—	48	0.94***
CcLG07	26	133.1	5.1	10	−0.84**	8	−0.95***	5	1.00***	5	0.14NS	8	−0.73*
CcLG08	34	119.3	3.5	16	−0.98***	—	—	13	−0.97***			24	0.98***
CcLG09	19	96.0	5.1	12	0.42NS	6	0.99***	9	0.91***	—	—	8	−0.95***
CcLG10	95	205.1	2.2	11	0.99***	3	1.00***	16	0.93***	2	—	8	0.99***
CcLG11	364	203.8	0.6	55	−0.66***	47	0.90***	82	−0.37***	173	0.00NS	102	0.107^NS^
Total	984	1609.5	1.6	192		133		204		352		301	

NS: not significantly different from zero at 0.05 probability level; *, ** and ***: significantly different from zero at 0.05, 0.01 and 0.001 probability levels, respectively. Pop1: ICP 11605 × ICP 14209, Pop2: ICP 8863 × ICP 11605, Pop3: HPL 24 × ICP 11605, Pop4: ICP 8863 × ICPL 87119, Pop5: ICP 5529 × ICP 11605, LG: linkage group, n: number of markers, ML: map length, AID: average inter-marker distance, “*r*”: correlation coefficient.

### Main effect QTLs for SPC and agronomic traits and their colocalization

Phenotyping data together with SNP genotyping data were used for QTL analysis in all five F_2_ populations using CIM and ICIM. Based on the phenotypic variance explained (PVE), identified M-QTLs were classified as major (≥10% PVE) and minor (<10% PVE). For each F_2_ population, details on M-QTLs identified have been explained below.

### Seed protein content

A total of 48 M-QTLs were detected for SPC across the five mapping populations (Table 7, Supplementary Table S2). Six of the M-QTLs were detected by both CIM and ICIM with two in Pop2 (CcLG03 and CcLG11) and Pop4 (CcLG02 and CcLG06), and one M-QTL each in Pop3 (CcLG02) and Pop5 (CcLG02). There were 13 major and 35 minor M-QTLs across the five populations. The PVE by each of the major M-QTLs ranged from 10.0% (Pop1, Pop3) to 23.5% (Pop3) while that of the minor M-QTLs ranged from 0.7% (Pop2) to 9.5% (Pop3). There were three major M-QTLs each in Pop1 and Pop5 and two each in Pop2 and Pop3. Eight of the major M-QTLs (three each in Pop1 and Pop5, and one each in Pop2 and Pop4) and 18 of the minor M-QTLs (two in Pop1, six each in Pop3 and Pop5, and four in Pop4) showed negative additive effects. The remaining five major M-QTLs (one in Pop2, two each in Pop3 and Pop4) and 17 minor M-QTLs (one in Pop1, four in Pop2, five each in Pop3 and Pop4, and two in Pop5) showed positive additive effects.

**Table 7:** Summary of main effect QTLs detected by composite interval mapping (CIM) and inclusive composite interval mapping (ICIM) for seed protein content (SPC), 100-seed weight (SW), seed yield (SY), days to first flowering (DFF) and growth habit (GH) in five F_2_ mapping populations of pigeonpea. Pop1: ICP 11605 × ICP 14209, Pop2: ICP 8863 × ICP 11605, Pop3: HPL 24 × ICP 11605, Pop4: ICP 8863 × ICPL 87119, Pop5: ICP 5529 × ICP 11605, PVE: phenotypic variation explained by a QTL. Number in parenthesis represents numbers of major M-QTLs (PVE% ≥ 10.0%). ^†^Number of QTLs detected by either CIM or ICIM. ^‡^Number of unique QTLs detected by either or both CIM and ICIM. PVE: phenotypic variation explained by a QTL. LOD: Logarithm of odds.

Population	QTL features	SPC		SW		SY		DFF		GH	
		CIM	ICIM	CIM	ICIM	CIM	ICIM	CIM	ICIM	CIM	ICIM
Pop1	No. QTLs^†^	5 (3)	1 (0)	4 (2)	4 (1)	6 (1)	1 (1)	6 (2)	1 (1)	3 (1)	3 (1)
	LOD	2.6–3.8	2.7	2.6–4.3	2.9–6.8	2.6–3.0	4.5	2.5–9.5	11.1	3.2–13.9	3.2–17.3
	PVE (%)	7.8–16.6	8.6	3.6–12.3	6.4–15.0	5.2–15.4	10.2	5.7–20.3	25.4	10.9–91.3	6.1–41.3
	Total No. QTLs^‡^	6		8		6		6		4	
Pop2	No. QTLs	4 (1)	4 (1)	2 (0)	2 (1)	4 (2)	6 (2)	6 (4)	3 (1)	5 (4)	2 (1)
	LOD	2.6–3.8	2.7–2.9	3.4–4.6	2.7–9.9	3.0–5.3	3.5–8.0	2.9–9.9	2.6–11.7	2.7–16.1	12.4–15.0
	PVE (%)	0.7–12.8	6.9–12.3	6.1–7.5	8.4–29.1	1.7–11.8	5.9–16.0	4.0–36.3	4.5–26.6	4.0–64.7	23.9–25.4
	Total No. QTLs	6		3		8		6		5	
Pop3	No. QTLs	6 (1)	8 (1)	5 (3)	2 (2)	8 (4)	6 (1)	3 (3)	6 (2)	5 (1)	4 (2)
	LOD	3.0–4.6	2.5–4.2	2.5–13.6	7.7–13.7	2.5–5.4	2.5–6.5	4.4–16.0	2.6–20.0	3.0–25.3	3.0–31.4
	PVE (%)	3.8–23.5	5.1–10.0	5.4–46.6	16.3–5.7	4.8–40.2	4.5–20.3	13.2–40.3	3.4–31.9	5.3–13.3	4.0–54.4
	Total No. QTLs	13		5		12		9		8	
Pop4	No. QTLs	10 (2)	4 (2)	6 (5)	4 (2)	5 (4)	6 (3)	19 (9)	7 (5)	—	—
	LOD	2.5–4.2	2.7–7.5	2.6–4.8	2.5–4.4	3.0–4.0	2.6–3.7	2.6–4.5	2.9–6.8	—	—
	PVE (%)	1.7–16.3	8.2–18.9	8.7–26.7	4.9–13.1	6.7–53.0	5.8–10.7	2.1–43.8	6.3–15.2	—	—
	Total No. QTLs	12		9		10		21			
Pop5	No. QTLs	7 (2)	5 (2)	2 (1)	4 (1)	1 (0)	4 (1)	4 (3)	3 (1)	8 (5)	5 (4)
	LOD	2.6–5.1	3.5–7.2	3.4–14.7	2.7–15.0	2.9	3.2–4.2	4.0–7.8	2.9–6.6	2.8–22.1	2.7–29.1
	PVE (%)	3.3–17.5	7.7–16.5	8.3–10.4	6.3–31.5	8.2	6.6–14.8	4.6–47.6	6.1–12.6	3.4–47.0	3.9–61.6
	Total No. QTLs	11		5		5		5		11	

### 100-seed weight

Thirty M-QTLs were detected for SW across the five mapping populations (Table 7, Supplementary Table S2). Five of the M-QTLs were detected by both CIM and ICIM with one each in Pop2 (CcLG01), Pop4 (CcLG03), and Pop5 (CcLG01), and two in Pop3 (CcLG01 and CcLG08. There were 16 major and 14 minor M-QTLs across the five populations. The PVE by each of the major M-QTLs ranged from 10.1% (Pop4) to 46.6% (Pop3) while that of the minor M-QTLs ranged from 3.6% (Pop1) to 9.4% (Pop4). There were three major M-QTLs in Pop1, one each in Pop2 and Pop5, four in Pop3 and seven in Pop4. Six of the major M-QTLs (four in Pop3 and two in Pop4), and six of the minor M-QTLs (four in Pop1, and one each in Pop3 and Pop5) showed negative additive effects. The remaining 10 major M-QTLs (three in Pop2, one each in Pop1 and Pop5, and five in Pop4) and eight minor M-QTLs (one in Pop1, two each in Pop2 and Pop4, and three in Pop5) showed positive additive effects.

### Seed yield

A total of 40 M-QTLs were detected for SY across the five mapping populations (Table 7, Supplementary Table S2). Seven of the M-QTLs were detected by both CIM and ICIM with one in Pop1 on CcLG03, two in Pop4 (CcLG03, CcLG011), three in Pop3 (CcLG02, CcLG04, CcLG11) and one in Pop4 (CcLG11). There were 17 major and 23 minor M-QTLs across the five populations. The PVE by each of the major M-QTLs ranged from 10.2% (Pop1) to 53.0% (Pop4) while that of the minor M-QTLs ranged from 1.7% (Pop2) to 9.8% (Pop4). There were two major M-QTLs each in Pop1 and Pop2, five in Pop3, seven in Pop4 and one in Pop5. Six of the major M-QTLs (one each in Pop1, Pop2, Pop3 and Pop5, and two in Pop4) and 15 of the minor M-QTLs (three each in Pop1, Pop3 and Pop4, four in Pop2, and two in Pop5) showed negative additive effects. The remaining 11 major M-QTLs (one each in Pop1 and Pop2, four in Pop3 and five in Pop4) and eight minor M-QTLs (one in Pop1, two each in Pop2 and Pop5, and three in Pop3 showed positive additive effects.

### Growth habit

Twenty eight M-QTLs were detected for GH across the four populations in which there was segregation for the trait (Table 7, Supplementary Table S2). Six of the M-QTLs were detected by both CIM and ICIM all on CcLG03 with two in Pop1, and one each in Pop2, Pop3 and Pop5. There were 19 major and nine minor M-QTLs across the populations. The PVE by each of the major M-QTLs ranged from 10.9% (Pop1) to 91.3% (Pop1) while that of the minor M-QTLs ranged from 3.4% (Pop5) to 6.5% (Pop3). There were three major M-QTLs in Pop1, four in Pop2, three in Pop3, and nine in Pop5. Four of the major M-QTLs (three in Pop3 and one in Pop5) and five of the minor M-QTLs (one each in Pop1, Pop2 and Pop5, and two in Pop3) showed negative additive effects to indeterminate GH. The remaining 15 major M-QTLs (three in Pop1, four in Pop2 and eight in Pop5) and four minor M-QTLs (three in Pop3 and one in Pop5) showed positive additive effects to indeterminate GH.

### Days to first flowering

In total, 47 M-QTLs were detected for DFF across the five populations (Table 7, Supplementary Table S2). Eleven of the M-QTLs were detected by both CIM and ICIM with one in Pop1 on CcLG03, two in Pop2 (CcLG03, CcLG11), five in Pop4 (CcLG01, CcLG06, CcLG08, CcLG11) and two in Pop5 (CcLG03). There were 24 major and 23 minor M-QTLs across the populations. The PVE by each of the major M-QTLs ranged from 10.9% (Pop4) to 47.6% (Pop5) while that of the minor M-QTLs ranged from 2.1% (Pop4) to 9.8% (Pop4). There were two major M-QTLs in Pop1, four in Pop2, five in Pop3, and 11 in Pop4. Seventeen of the major M-QTLs (two each in Pop1 and Pop5, three in Pop2, one in Pop3, and nine in Pop4), and eight of the minor M-QTLs (two in Pop1, one each in Pop2, Pop3 and Pop5; and three in Pop4) showed negative additive effects to delayed DFF. The remaining seven major M-QTLs (one in Pop1, four in Pop3 and two in Pop5) and 15 minor M-QTLs (two each in Pop1 and Pop2, three in Pop3, seven in Pop4 and one in Pop5) showed positive additive effects to delayed DFF.

### QTL colocalization and correlations among traits

One M-QTL region in Pop1 for which SPC and DFF shared one of the flanking SNPs (S3_18226407) on CcLG03 showed negative and positive additive effects on SPC and DFF, respectively (Supplementary Table S2; Fig. S1), indicating it contributed to the observed negative correlation between the two traits (Table 2). Another M-QTL region region flanked by SNPs S3_14813065 and S3_14778845 also in Pop1 on CcLG03 had positive and negative additive effects on GH and DFF, respectively, and likely contributed to the observed negative correlation between the two traits.

Similarly, M-QTL flanked by SNPs S3_22234078 and S3_19578263 on CcLG03 in Pop2 showed pleiotropic effect on SPC, SY, GH and DFF (Supplementary Table S2, Supplementary Fig. S2). The M-QTL region displayed positive additive effects on SPC and GH, but negative additive effects on DFF and SY, possibly explaining the high positive correlation between SPC and GH, and negative correlation of SPC with DFF and SY, respectively. In the same Pop2, SPC shared an M-QTL with SW on CcLG01 in a region fanked by SNPs S1_15372966 and S1_9033631 with positive additive effect on both traits thus possibly contributing to the positive correlation between the two traits (Table 2). Another region in the same Pop2 on CcLG11 flanked by SNPs S11_21940736 and S11_18137395 conditioned SPC and SY having positive and negative additive effects, respectively, and likely resulted to the negative correlation between the two traits (Table 2).

In Pop3, three M-QTL regions flanked by SNPs S3_28538775 and S3_22913898, S3_18154848 and S3_17193829, and S3_18154875 and S3_14813065 all on CcLG03 affected SPC, GH and DFF with additive effects being negative for SPC and GH, and positive in two and negative in one of the M-QTLs for DFF (Supplementary Tables S2, Supplementary Fig. S3) possibly contributing to the positive correlation between SPC and GH, and negative correlation between SPC and DFF, and between GH and DFF (Table 2). Another M-QTL region in Pop3 on CcLG04 flanked by SNPs S4_3592410 and S4_2761907 had negative and positive additive effects on SY and GH, respectively, and likely contributed to the negative correlations between the two traits. A third M-QTL region in the same Pop3 flanked by SNPs S2_2989918 and S2_2144739 on CcLG02 conditioned both SPC and SY having positive and negative additive effects, respectively, and likely contributed to the negative though none significant correlation between the traits.

Similarly, a QTL region flanked by SNPs S1_1145802 and S1_11242012 on CcLG01 in Pop4 conditioned both SY and DFF with positive and negative additive effects, respectively (Supplementary Table S2, Fig. S4), and likely contributed to the negative though non-significant correlation between the traits (Table 2). Additionally, a QTL on CcLG02, flanked by SNPs S2_11771536 and S2_10960200 conditioned SW and DFF with positive and negative additive effects, respectively, and likely contributed to the negative correlation between the two traits. A major M-QTL on CcLG10 flanked by SNPs S10_15140940 and S10_632618 influenced both SW and SY with positive additive effects on both traits, indicating it contributed to the positive correlation between the traits. There were two tight linkages (0.1 cM distance), one between M-QTLs for SPC and DFF, and another between SPC and SY both on CcLG11.

Neither CIM nor ICIM detected any overlap or tight linkage of M-QTLs in Pop5 between any of the measured traits (Supplementary Table S2, Fig. S5) although significant correlations were detected between SPC and SY, GH and DFF, SW and DFF, SY and DFF, and SY and GH (Table 2).

### Consensus genetic and main effect QTLs across populations

Forty-one, 26, 27, 28 and 31 out of a total of 48, 30 40, 28 and 47 M-QTLs for SPC, SW, SY, GH and DFF, respectively, from the five mapping populations could be projected onto the consensus genetic map. Twenty-four (60%) of the projected SPC M-QTLs could be placed into six consensus QTL regions (Supplementary Fig. S7). The consensus SPC QTLs contained M-QTLs from two populations (*Consensus-PROT-QTL 1*, *Consensus-PROT-QTL 2* and *Consensus-PROT-QTL 5*), three populations (*Consensus-PROT-QTL 3*) and four populations (*Consensus-PROT-QTL* 4 and *Consensus-PROT-QTL* 6). Out of the 26 M-QTLs for SW projected onto the consensus genetic map, only 13 could be collapsed into four consensus QTL regions, namely *Consensus-SW-QTL 1* with QTLs from two populations on CcLG01 and *Consensus-SW-QTL 2* with QTLs from three populations on CcLG01, *Consensus-SW-QTL 3* on CcLG06 with QTLs from 2 populations and *Consensus-SW-QTL 4* on CcLG08 with QTLs from 3 populations. For SY, only four out of 27 M-QTLs projected onto the consensus genetic map could be put into two consensus regions. *Consensus-SY-QTL 1* and *Consensus-SY-QTL 2* on CcLG03 and CcLG11 consisted of M-QTLs from two populations  each. For DFF 26 M-QTLs could be put in to four consensus QTL regions with QTLs from two populations in *Consensus-DFF- QTL 1* on CcLG02, four populations (*Consensus-DFF-QTL 2)* on CcLG03 and three populations (*Consensus-DFF-QTL 3* and *Consensus-DFF-QTL 4*) on CcLG11. In the case of GH, 20 M-QTLs from four populations could be put in to one consensus QTL region (*Consensus-GH-QTL 1*) on CcLG03.

Ten QTL clusters could be recognised (Supplementary Fig. S7). *QTL-Cluster 1* on CcLG01 and *QTL-Cluster 6* on CcLG04 each harboured one minor M-QTL for each of SPC and SW. *QTL-Cluster 2* on CcLG02, *QTL-Cluster 9* on CcLG11 and *QTL-Cluster 10* on CcLG11 each haboured M-QTLs for all measured traits. Two M-QTLs each for SPC, SW and SY in *QTL-Cluster 3* and *QTL-Cluster 5* both on CcLG02 contained M-QTLs for SPC, SY and DFF. *QTL-Cluster 4* on CcLG03 harboured M-QTLs for SPC, SY, GH and DFF, while *QTL-Cluster* 7 on CcLG06 contained M-QTLs for SW and SY only. *QTL-Cluster* 8 on CcLG09 contained M-QTLs for SPC and GH only. There were two M-QTLs for SPC, one each for SW, GH and DFF, and three for SY in *QTL-Cluster 2*. Of the M-QTLs in *QTL-Cluster 2*, there were two for each of SPC, SW and SY, and one for DFF with PVE ≥ 10.0%. In the case of *QTL-Cluster 3*, two M-QTLs for SPC and one for SY were major, while in *QTL-Cluster 4* two, 15, 12 and one M-QTLs for SPC, GH, DFF, and SY, respectively, showed large effects (>10.0%). In contrast, *QTL-Cluster 5* harboured only one major M-QTL for SY colocalising with minor M-QTLs (<10.0%) for other traits. *QTL-clusters 6*, *7* and *8* harboured only minor M-QTLs, while *QTL-Cluster 9* contained two major M-QTLs for each of SPC and SY, three for each of DFF and SW, and one for GH. *QTL-Cluster* 10 was made up of one major M-QTL for each of SPC, GH, SW and SY, and two for DFF.

### Epistatic QTLs

To gain more insight into the complexity of the genetic control of SPC and its relationship with other traits, epistatic QTLs (E-QTLs) were mapped in each of the five F_2_ populations using QTL Icimapping software v4.0 (http://www.isbreeding.net/software/?type=detail&id=14) (Table 8; Supplementary Table S3). Pop2 had the highest number of E-QTLs (173) while Pop4 had the lowest number (52) across traits. Among traits, SPC had the lowest number of E-QTLs ranging from two in Pop3 to 11 in Pop1 while GH had the highest number ranging from 40 in Pop1 to 56 in Pop2 (Table 8). The E-QTLs were detected on all CcLGs in each population. Overall, E-QTLs made large contributions to the phenotypic variations of the measured traits ranging from 6.3% for DFF in Pop1 to 99.4% for GH in Pop2 (Table 8). In the case of SPC as the core trait in this study, E-QTLs accounted for 12.8 to 31.2% (Pop1), 55.0 to 69.8% (Pop2), 19.3 to 21.2% (Pop3), 9.8 to 30.5% (Pop4) and 9.5 to 21.2% (Pop5) of the within-population SPC variations (Table 8; Supplementary Table S3). For the agronomic traits, there were five to 26 E-QTLs with PVE of 6.3 to 38.4% for DFF, 40 to 56 E-QTLs (PVE = 10.4 to 99.4%) for GH, eight to 63 (PVE = 11.5 to 41.8%) for SW, 12 to 39 (PVE = 10.6 to 38.5%) for SY. No common within-trait E-QTL pairs were detected in all of the five populations, however, eight SNPs were each found to flank at least one member of an E-QTL pair for GH and SW in two to three populations, while four SNPs each flanking at least one member of E-QTL pair for SY were detected in two populations. E-QTLs for SPC and DFF were highly population-specific without any commonly shared markers among populations.

**Table 8:** Summary of epistatic QTLs detected for seed protein content (SPC), 100-seed weight (SW), seed yield (SY), days to first flowering (DFF) and growth habit (GH) in five F_2_ mapping populations of pigeonpea. Pop1: ICP 11605 × ICP 14209, Pop2: ICP 8863 × ICP 11605, Pop3: HPL 24 × ICP 11605, Pop4: ICP 8863 × ICPL 87119, Pop5: ICP 5529 × ICP 11605. E-QTLs: epistatic QTLs, PVE: phenotypic variation explained. Number in parenthesis represents numbers of major E-QTLs (PVE% ≥ 10.0%), PVE: phenotypic variation explained by a QTL, LOD: Logarithm of odds.

Population	E-QTL features	SPC	SW	SY	DFF	GH
Pop1	Number of E-QTLs	11 (11)	8 (8)	29 (29)	5 (5)	40 (1)
	LOD	5.1–6.6	5.0–5.6	5.1–8.2	5.0–5.5	9.4–79.6
	PVE (%)	12.8–31.2	14.6–25.3	12.9–38.5	10.4–33.4	10.9–91 0.3
Pop2	Number of E-QTLs	9 (9)	63 (63)	19 (19)	26 (26)	56 (56)
	LOD	5.2–7.5	6.6–17.1	5.0–7.3	5.0–8.9	5.0–1132.5
	PVE (%)	55.0–69.8	29.8–41.8	10.6–36.4	14.8–44.3	10.4–99.4
Pop3	Number of E-QTLs	2 (2)	53 (53)	30 (30)	10 (6)	50 (50)
	LOD	5.2–5.3	5.0–9.9	5.0–8.5	5.0–6.2	5.1–41.8
	PVE (%)	19.3–21.2	14.6–39.8	14.6–37.1	6.3–14.6	14.1–96.0
Pop4	Number of E-QTLs	8 (7)	20 (20)	12 (12)	12 (12)	—
	LOD	5.0–6.3	5.1–7.0	5.1–7.2	5.1–6.2	—
	PVE (%)	9.8–30.5	14.2–25.6	12.0–23.8	12.0–30.1	—
Pop5	Number of E-QTLs	4 (3)	20 (20)	39 (39)	5 (5)	42 (42)
	LOD	5.2–6.9	5.1–7.3	5.0–8.7	5.1–7.0	5.1–16.6
	PVE (%)	9.5–21.2	11.5–30.0	15.9–35.1	14.7–23.6	10.6–74.8

### E-QTLs shared among traits within and across populations

**Figure 3:**
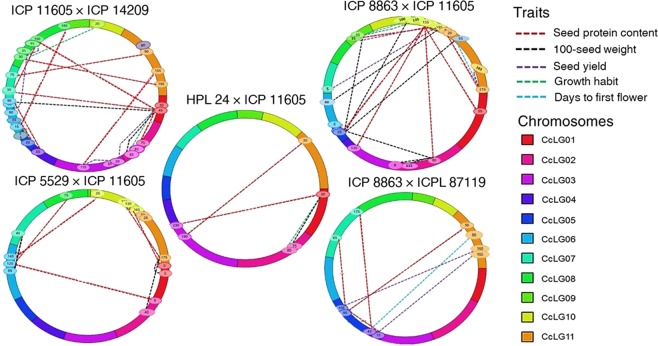
Epistatic QTLs conditioning seed protein content and agronomic traits in five F_2_ mapping populations of pigeonpea as revealed by QTL IciMapping Software v4.0 (http://www.isbreeding.net/software/?t™ype=detail&id=14).

The number of E-QTL pairs shared between SPC and the agronomic traits were variable depending on the population (Fig. 3). In Pop1, SPC shared E-QTLs with SW, SY and GH. In Pop2, SPC shared E-QTLs with SW, SY, DFF and GH, while in Pop3, SPC shared E-QTL markers with SW and GH. In Pop4, SPC shared two E-QTLs with SY, and one E-QTL with DFF. In Pop5, two E-QTLs for SPC were shared with SW, and one with SY. The five populations also had some E-QTL pairs in common but with varying effects on measured traits. For example, SNP S5_4199522 on CcLG05 flanked several members of E-QTL pairs affecting SY in Pop3 and Pop5, GH in Pop2, Pop3 and Pop5, DFF in Pop5, and SW and SPC in Pop2. The E-QTL pairs with at least one member flanked by this SNP explained 15.1% (GH in Pop2) to 64.8% (SPC in Pop2) of the observed phenotypic variation. Another SNP, S7_14683829, flanked a member of E-QTL pairs to influence GH in Pop1, Pop2, Pop3 and Pop5, SW in Pop3, SPC and SY. Similarly, a number of E-QTLs having SNP S7_14683829 as one of the flanking markers on CcLG07 influenced GH in Pop1 and Pop2, SW in Pop3 and all five measured traits in Pop5. Interestingly, an Indel marker (s3-20698771) derived from *CcTFL1* on CcLG03 which co-segregates with the *Dt1* locus^12^ flanked three epistatically acting QTLs to influence GH with PVEs of 73.8, 74.3 and 69.4% in Pop5. A 2-phosphoglycerate kinase (2PGK) gene-derived non-synonymous SNP (nsSNP, s4-496463^25^) together with SNP S4_1710877 flanked a QTL on CcLG04 which interacted with other QTLs on CcLG07, CcLG08 and CcLG11 to influence GH (17.3 and 18.2%) and SW (19.5 and 19.5%) in Pop5. The Indel marker and the nsSNP also separately flanked a member of a pair of two other E-QTLs to influence GH with a PVE as high as 73.8% in the same population.

## Discussion

To detect the QTLs conditioning SPC and its relationship with agronomic traits, we used parental lines with only moderate contrast in SPC (0.8 to 3.5%) between any pair parents of a cross. Wide segregation among the F_2_ progenies of a cross beyond what is expected from parental values was observed in the F_2_ populations, indicating transgressive segregation, a phenomenon commonly observed for SPC in other legumes such as soybean^26,27^ and pea^28,29^. Strong quantitative variations with transgression for days to flowering, SY and SW were also observed in the present study, consistent with reports of earlier studies in segregating populations of pigeonpea^10^.

Given that a 5-cM SNP spacing is considered sufficiently dense for optimized QTL detection power^30^, the SNP marker spacing in each of the five populations in the present study provides adequate power to detect a QTL. Marker segregation distortion was observed in all the five populations with similar proportion of markers showing deviation from expectation. Segregation distortion could have resulted from various factors such as residual heterozygosity, gametic or zygotic selections and genotyping errors^31^. It is a common phenomenon observed in both intra- and inter-specific crosses and has been reported in several crops including pigeonpea^9,12,13^ and chickpea^32^. Although distorted markers have generally been discarded in earlier studies, evidence indicates that distorted markers can be potentially helpful in the detection of QTLs^33^. It has also been noted that discarding distorted markers could possibly remove substantial amounts of information and reduce genome coverage^34^. Thus, in the present study distorted markers segregating in 1:2:1 Mendelian ratio with χ^2^ cutoff P ≥ 10^−9^ were retained for genetic map construction. By integrating the five component genetic maps into a consensus genetic map, conserved marker orders were observed among the five genetic maps that could be attributed to use of relatively similar population size (137 to 179), same type of mapping populations (all F_2_s) and same type of marker system (GBS-derived SNPs)^9^. The constructed genetic maps were then used for QTL analysis to map genomic regions associated with SPC and four agronomic traits.

In pigeonpea, QTLs have been mapped for plant type and earliness including days to flowering and growth habit^10,12,35^ and disease resistance^13^. However, such studies have lacked for SPC and it is only till recently that we developed gene-derived sequence-based markers using whole genome resequencing of pigeonpea parental lines^25^. Genomic regions associated with SPC and correlated traits offer opportunity to develop varieties with enhanced SPC and stable yield using genomics-assisted breeding approaches. In this context, analyses of QTLs for SPC and agronomic characters (SW, SY, DFF and GH) were conducted based on five populations. To ensure reliability of detected QTLs the present investigation used two methods, CIM and ICIM. ICIM also facilitated the detection of E-QTLs. Although both methods detected comparable number of M-QTLs across traits in the studied populations, CIM detected slightly more M-QTLs than ICIM, which agrees with results of an earlier study^36^. Three or more M-QTLs were detected by both programs while ICIM detected two or more E-QTLs for each trait in each of the studied populations. The involvement of several M- and E-QTLs for each of the measured traits explained observed variations in the traits and indicate quantitative inheritance. Colocalized M-QTLs as well as E-QTLs explained trait correlations in each of the populations studied.

The detection of two to three major and several modifier/minor effect M-QTLs for SPC spread on nearly all linkage groups of pigeonpea in each of the five studied populations is in agreement with results obtained in soybean^26,27^. The M-QTLs for SPC were highly population-specific although three CcLGs contained at least one major M-QTL in two to three of the five populations. The three CcLGs (CcLG02, CcLG03 and CcLG11) also contained M-QTLs with the highest PVE than M-QTLs on the other CcLGs suggesting their relative importance in harbouring genomic regions governing SPC in the pigeonpea. The localisation of the major M-QTLs on CcLG02 in three of the five populations in the present study could be supported by the detection on the same chromosome of some genes known for their functional role in seed storage protein accumulation such as NADH-GOGAT^25^, or for their location in the vicinity of QTL regions associated with variability of SPC in plants^37^. By projecting the population specific M-QTLs to the consensus genetic map, six consensus genomic regions, each comprising M-QTLs for SPC from two to four populations were generated. Such consensus regions may be targeted for further investigation in future studies. Across the five mapping populations, SPC increasing alleles were contributed by both the low and high trait parents. The majority of the trait increasing alleles from the low trait parent were minor except in Pop2 and Pop4 for which the respective low SPC parent contributed one major M-QTL each. Whereas it has been concluded that SPC in pigeonpea is conditioned by recessive oligo-genes^38^, it is apparent from our results that the trait is polygenic with a combination of gene actions conditioning its variation in the crop. This observation is in agreement with earlier conclusions that SPC is conditioned by both additive and non-additive genes in pigeonpea^39^. Predominance of non-additive types of gene action in the present study is also in agreement with earlier observations in pigeonpea^40^ and other legumes^41–43^.

The detection of at least two genomic regions for DFF in each population is in agreement with reports of previous studies in pigeonpea^44^, but contrasts with the results of Kumawat *et al*.^10^ who reported only one major M-QTL for the trait. The detection of the majority of M-QTLs for DFF on CcLG03 in four of the five mapping populations is consistent with an earlier detection of a well-known flowering time gene *CcTFL1* on the same CcLG03^12,45^. Most of the M-QTLs for DFF and GH tended to colocalize to the same genomic regions, flanked by the same SNP loci, which is in agreement with observations that genes conditioning flowering time have pleiotropic effect on GH^35,45,46^. The pleiotropic M-QTLs largely acted recessively in conditioning GH, consistent with observations in other crops^47^, but the same loci acted either additively, dominantly, partially dominantly or overdominantly on DFF which agrees with earlier reports on genetics of early flowering^39,48–50^.

The detection of at least two and up to seven major and several minor M-QTLs to condition SW in the crop indicates quantitative inheritance for the trait, consistent with findings in other legume crops^28,51–54^. A genomic region on CcLG01 consistently showed highest PVE in three of the five mapping populations suggesting a common major genomic loci segregating in a wide range of genetic backgrounds, similar to reports in soybean^51^. With the exception of one major M-QTL on CcLG01, all other M-QTLs for SW showed population specificity. The diversity of QTL gene action observed for SW in this study mirrors earlier reports where recessiveness, dominance and overdominance have been reported to condition SW in plants^55–57^.

QTLs for SY (on plant basis) have been mapped in other crops^58,59^, but the present study is the first in pigeonpea. The detection of highly population-specific minor and major effect M-QTLs for SY on nearly all CcLGs points to a complex genetic architecture of the trait. Detection of large effect population-specific M-QTLs on CcLG10 in three of the five populations, and minor and major M-QTLs on CcLG01, CcLG02, CcLG03 and CcLG11 in three to four populations suggests the relative importance of the chromosomes in hosting genomic regions associated with the trait.

The pervasiveness of population-specific M-QTLs for SPC, SY, SW and to a lesser extent for DFF could be attributed to effects of population size or marker coverage^20^. However, this is unlikely because population-specific M-QTLs of relatively minor effects ranging from 0.7% to 8.6% across traits were mapped in the five populations. Rather, it is possible that a QTL detected in a certain cross may not be detected in another cross because the parents of the second cross carry identical alleles at the same locus^17,20^.

E-QTLs were detected that explained additional phenotypic variation for SPC and the other traits. Effects of E-QTLs have been reported in other legume crops such as soybean for SPC^26,27,60^ and SW^60,61^. Similarly, E-QTLs for SW, SY, flowering time and GH have been reported in common bean^62^. The large number of E-QTLs for SPC and for the agronomic traits identified in present study indicates that QTLs with minor effects or no effect interact with each other to influence expression of the traits. Such scenarios have been reported in other crop plants^60,62^. Uniquely, Pop2 displayed the highest contribution of E-QTL effects on phenotypic variation of all measured traits in the present study. It is likely that the relatively low marker density in Pop2 contributed to the high PVE of the E-QTLs in this population. Across populations, the number of E-QTLs detected also varied by trait. The pattern of contributions of M-QTLs vs E-QTLs to phenotypic variation for the studied traits seemed to be highly genetic background-dependent as has been frequently reported in other crops^62–65^^.^

In an earlier study, we reported a number of putative candidate gene-based nsSNP for SPC, some of which significantly cosegregated with the trait in an F_2_ validation population, ICP 5529 × ICP 11605^25^. Similarly, Saxena *et al*.^12^ developed a *CcFTL1* gene-based Indel marker whose cosegregation with the determinate GH locus *Dt1* was validated in the same F_2_ population. The same F_2_ ICP 5529 × ICP 11605 population is one of the populations used in the present study. Interestingly, a number of the gene-derived markers also flanked M-QTLs and/or E-QTLs with significant effect on the agronomic traits. For example, the detection of a major M-QTL for GH (qGH-icim-4.1; PVE = 13.1%) on CcLG04 with one of the flanking markers being a 2PGK gene-derived nsSNP likely indicates the role of the gene on GH in the crop. A further evidence for the influence of the 2PGK gene on GH is that qGH-icim-4.1 epistatically interacted with another major M-QTL for GH (qGH-icim-3.2; PVE = 61.6%) on CcLG03 resulting in an E-QTL with a much higher PVE of 73.8% than that of the individual M-QTLs. One of the flanking markers to qGH-icim-3.2 is the CcTFL1 gene-derived Indel marker^12^. The involvement of the 2PGK gene-derived nsSNP in another epistatically acting QTL to influence SW also agrees with colocalization of 2PGK gene with a QTL for SW in pea^28^. One more gene of interest from our results is Sucrose synthase (Sus6) from which a derived nsSNP flanked an epistatically acting QTL on CcLG01 to influence SW with the resultant E-QTL having a major effect (11.5%). Sucrose synthase has for long been known to play a major role in SW in several crop plants as mentioned in Turner *et al*.^66^. The role of Sus6 as one of the possible determinants of SW in our study is further indicated by location of the derived nsSNP in the vicinity of *Consensus-SW-QTL 1* comprising a major SW M-QTL from Pop2 and a minor SW M-QTL from Pop3 on CcLG01 of the consensus genetic map. The same nsSNP was only 3.1 cM away from a major M-QTL on CcLG01 in Pop5.

In this study, two lines of evidence revealed the associations between SPC and the other plant traits, and that the nature of the associations is genetic background-dependent. First, the phenotypic correlation analysis showed that SPC associates positively with GH and SW and negatively with DFF and SY. The pattern of correlation of SPC with SW is consistent with results of earlier studies which showed that the two traits associate either positively or negatively and sometimes non-significantly depending on genetic material used^6^. In the case of SPC with DFF, negative though small and none significant relationships have been reported in pigeonpea^8,67^. The negative and relatively weak correlation between SPC and SY in the present study is consistent and within the range previously reported in pigeonpea^8,67,68^, and soybean^69,70^. No relationship between SPC and GH has been reported in pigeonpea before. However, the indeterminate and determinate GH in soybean have been reported to be associated with high and low SPC, respectively^71^. Significant correlation of SPC with morphological and growth-related traits have also been reported in pea^28^.

Second, colocalization of M-QTLs and shared E-QTLs for SPC with that of the other traits were found that possibly explains trait correlations. For instance, the colocalization of M-QTLs for SPC with M-QTLs for DFF with opposite allelic effects could explain the negative correlations between SPC and DFF in Pop1, Pop2, Pop3 and Pop4 though the correlations were non-significant in Pop2 and Pop4. Similarly, the colocalization of M-QTLs for SPC and M-QTLs for GH with allelic effects in the same direction in Pop1, Pop2 and Pop3 explains positive correlation between the two traits. Likewise, correlation of SPC with SW in Pop2 could be explained by the overlapping M-QTLs on CcLG02 with allelic effects in the same direction. While the negative correlation of SPC with SY could be attributed to opposing effect of colocalized M-QTLs for the two traits such as in Pop2.

However, not all correlations of SPC with agronomic traits could be explained by colocalization of M-QTLs, for instance, GH and SY showed relatively strong correlation with SPC in Pop4 but no M-QTL overlaps were present. Therefore, presence of E-QTLs shared between SPC and the agronomic traits were searched that could explain correlations that are not explained by the M-QTLs. The phenomenon where one E-QTL affects expression of more than one trait have been termed ‘epistatic pleiotropy’^72^. In this regard, the majority of epistatic pleiotropy involving SPC and other traits in the present study are the type in which the effects of a given pleiotropic locus are dependent upon the alleles present at the other loci^73^. For example, in Pop1 a QTL on CcLG01 flanked by markers S1_4757043 and S1_1575466, affected (i) SPC when it interacted with other QTLs on CcLG07 and CcLG08, (ii) SW when it interacted with QTLs on CcLG02 and CcLG06, and (iii) SY when it interacted with a QTL on CcLG03.

Similarly, a single epistatically pleiotropic QTL (EP-QTL) on CcLG01 (*S1_887236* and *S1_33*9*9209*) in Pop3 influenced the expression of SPC, SW and GH when it interacted with other QTLs on CcLG02 and CcLG03 and possibly contributed to the significant covariation between SPC and SW, and SPC and GH. Such EP-QTLs involving SPC were widespread among populations, and in some cases provided the only explanation to phenotypic correlation between SPC and the other traits. For instance, the significant correlation between SPC and SY in Pop4 in the absence of overlaps in their M-QTLs could be explained by EP-QTL on CcLG07 flanked by markers S7_14683829 and S7_14588865. The same EP-QTL also influenced expression of SW and DFF although the two traits show weak and non-significant correlation with SPC. In Pop5, three EP-QTLs were detected, two of which influenced SPC and SY, and one influenced SPC and SW even though no significant relationships of SPC with SW and SY were found. Tuberosa *et al*.^74^ noted that the occurrence of QTL colocalization for multiple traits that possibly share a common morpho-physiological basis, or that are reasonably associated on a cause-effect basis, should lower the chance of declaring false positives in the regions where QTLs overlap.

In conclusion, two to three major M-QTLs in the presence of several modifier/minor effect QTLs, and with additive and non-additive QTL gene action types including epistasis, control the expression of SPC in the present study. Overlaps of main effect and E-QTLs explain the correlations between SPC and agronomic traits. Projection of M-QTLs for SPC and agronomic traits onto the consensus map revealed common genomic regions governing SPC and its relationship with agronomic traits across different genetic backgrounds. Among the genomic regions, *QTL Cluster 5* (CcLG03), *QTL Cluster 10* (CcLG11) and *QTL Cluster 9* (CcLG11), in order of increasing importance, harboured M-QTLs for two or more traits and therefore may be targeted for the simultaneous improvement of the associated characters. More trait-specific regions such as *Consensus-PROT-QTL 1* and *Consensus-PROT-QTL 2* (CcLG02), *Consensus-PROT-QTL 4* (CcLG04), *Consensus-PROT-QTL 6* (CcLG11), *Consensus-SW-QTL 1* and *Consensus-SW-QTL 2* (CcLG01), *Consensus-DFF-QTL 1* (CcLG03) and *Consensus-GH-QTL 1* (CcLG03) as well as the more population-specific SY M-QTLs with large PVEs on CcLG03 (Pop1 and Pop2), CcLG04 (Pop3), CcLG05 (Pop4), CcLG10 (Pop3 and Pop4) and CcLG11 (Pop2) could also be targeted for the improvement of the traits. The genomic regions identified in the present study would pave the path for early generation screening of large segregating populations or screening of germplasm resources and haplotype based breeding for identification of plants/genotypes carrying favourable alleles/haplotypes and minimizing the negative correlation effect of other traits on SPC. By this way high yielding lines with higher SPC could be developed with less resources and time. However, the large contribution of epistasis to the variation and correlation among the traits and the presence of a large number of population-specific M-QTLs for each of the traits, suggests that breeding approaches that target genome wide variations such as genomic selection^75^ would be an alternative in achieving larger genetic gains for both SPC and yield in a shorter period. Further, the validation of the results in additional germplasm and under diverse environmental conditions may be necessary to determine the stability of the QTLs identified as well as facilitate detection of other loci.

## Methods

### Crossing parents and seed protein content

Six pigeonpea genotypes that included ICP 11605, ICP 8863, ICP 14209, HPL 24, ICP 5529 and ICPL 87119 were used in the present study. ICP 8863 was selected from landrace ICP 7626 (P-15-3-3) and it is widely cultivated in India. It is high yielding with 100-seed weight of ~9.5 g and matures in 150–160 days. It is resistant to fusarium wilt (FW) but susceptible to sterility mosaic (SM) virus^76^. ICP 8863 has moderate SPC of ~22.0%. ICP 11605 (ICPL 151) was selected from the cross ICP 6997 × Prabhat. It is a determinate cultivar, yielding ~1.03 t/ha with 100-seed weight of 10 g and matures in 120–130 days^77^ and has a low SPC of ~20.9%. ICP 14209 is a landrace variety with moderate SPC (23.0%). ICPL 87119 was developed from the cross ICP 1-6-W3–Wl × C 11 and it is widely adapted and cultivated in India. It matures in 160–180 days, is high yielding and has resistance to FW and SM^78^. It is low in SPC (~19.3%). HPL 24 is an advanced breeding line derived from the cross of cultivar *C*. *cajan* cv Baigani × *C*. *scarabaeoides* previously reported to have ~30% SPC^6^. It is indeterminate and of medium maturity duration. ICP 5529 with pedigree P-4864-1, originated from India. It is indeterminate with medium maturity duration and with SPC indicated to be 27%.

### Mapping populations, field experiments and phenotyping

In order to develop the mapping populations (F_2_), five crosses were made: ICP 11605 × ICP 14209, ICP 8863 × ICP 11605, HPL 24 × ICP 11605, ICP 8863 × ICPL 87119 and ICP 5529 × ICP 11605. For brevity, the populations are hereafter referred to as Pop1, Pop2, Pop3, Pop4 and Pop5, respectively. One F_1_ plant was selfed to generate F_2_ seeds in each of the five populations. For trait evaluation, the parents and 350 to 400 F_2_ seeds were sown under field conditions to ensure an adequate number of plants. Sowing was done in 4 m long rows spaced 75 cm apart and 30 cm plant to plant distance within a row. Plot sizes were two rows for each of the two parents and 25 to 28 rows in the F_2_. All cultural practices were carried out. At maturity individual pods from individual plants were carefully hand-harvested leaving out plants at the beginning and at the end of each row and those at the field borders to avoid border effects. Sun drying was done for one week before threshing and another one week after threshing to ensure uniform reduction in seed moisture content. Seed protein content was measured as described in Obala *et al*.^25,67^. Besides SPC, data were also recorded for SW in grams, SY in grams per plant, DFF, and GH scored as determinate or indeterminate.

### DNA isolation and genotyping

Total genomic DNA (gDNA) from 188 F_2_ plants and the parents from each of the five mapping populations were isolated and genotyped-by-sequencing as described in Saxena *et al*.^12,13^. Briefly, the sequence reads obtained from the Illumina HiSeq. 2500 platform were used for SNP identification and genotyping using GBS analysis pipeline implemented in TASSEL v4.020 (TASSEL-GBS)^79^. Firstly, the reads were sorted, separated according to the sample barcodes and trimmed to first 64 bases starting from the enzyme cut site. Reads containing ‘N’ within the first 64 bases and reads with >50% of low-quality base pairs (Phred <5%) were discarded. The filtered, high-quality reads from each sample were aligned to the pigeonpea draft genome sequence (*C*. *cajan* v1.0)^2^ using Bowtie 2 sequence alignment software. The alignment file was processed through TASSEL-GBS pipeline for SNP calling and genotyping. The quality of SNPs called in each F_2_ individual was compared with the SNPs identified in parental lines. The parental line SNPs were obtained from whole-genome resequencing (WGRS) data^80^. SNPs having confident parental calls were considered for further analysis. SNPs and F_2_ individuals having more than 30% and 70% missing data, respectively, were filtered out. The quality SNP data was used for construction of genetic maps and QTL analysis.

### Construction of population-specific genetic maps

Four of the five population-specific genetic maps were constructed in the present study while the remaining one population-specific map was constructed under a separate project^12^. The construction of all five population-specific genetic maps followed the same procedure as described in Saxena *et al*.^12,13^.

### Construction of consensus genetic map

Genotyping data from the five F_2_ genetic maps were used to develop a consensus genetic map using JoinMap v4.1 following the procedure described by Bohra *et al*.^9^. To assess the level of correspondence in the order of markers between consensus and component genetic maps, correlation coefficients (*r*) were calculated from marker positions in consensus and individual genetic maps and their significance were tested. To further visualize the extent of correlation between consensus and component maps, scatter plots were generated between each of the consensus linkage group and corresponding component linkage group from all the populations. A comparative mapping programme CMap v1.01^81^ was used to align all developed genetic maps together to visually assess the congruency of marker orders.

### QTL mapping

Composite interval mapping (CIM) implemented in Windows QTL Cartographer v2.5^82^ and inclusive composite interval mapping (ICIM) implemented in QTL Icimapping v4.0^83^ were used to detect main effect QTLs (M-QTLs) while epistatic QTLs (E-QTLs) were detected using ICIM. The advantage of both CIM and ICIM is that they are regression-based and are therefore robust against non-Gaussian trait distribution^84^. For CIM, the Standard Model 6, walk speed of 1.0 cM, and forward-backward stepwise regression for setting number of marker cofactors for background control were used to identify M-QTLs. To leave out signals within 10.0 cM distance on either side of the flanking markers or QTL test site, a window size of 10 cM was used. Thresholds for declaring QTLs were determined by 1000 permutations at significance of 0.05.

In using ICIM to detect M-QTLs, marker selection was performed just once using stepwise regression and considering all marker information simultaneously^85^. Phenotypic values were then adjusted by all markers retained in the regression equation, except the two markers flanking the current mapping interval. Permutation tests were conducted using SPC in the five F_2_ mapping populations to determine the criteria for model selection in the first step of ICIM. For all five F_2_ populations, the probability of a marker moving into the model corresponding to the overall type I error α = 0.05 was approximately 10^−5^. The probability of a marker moving out of the model was set at twice the probability of a marker moving into the model. The LOD threshold to declare the existence of a QTL was calculated by permutation tests as well. However, because of the always conservative nature of thresholds retained from permutation tests^86^, a default LOD threshold of 2.5 was used to report QTLs and determine common (consensus) QTLs across populations.

Furthermore, where M-QTL identified by CIM was also detected by ICIM, the region was considered as one QTL. Similarly, where an M-QTL for a given trait identified by either CIM or ICIM colocalize with M-QTL(s) of other traits detected by either of the two methods, the region was treated as a region of co-localisation. Type of gene action for each M-QTL was derived from the dominance coefficient (*h*) defined as the ratio between the observed QTL dominance effect (*d*) and absolute value of QTL additive effects (|*a*|)^87^. We used the absolute value of additive effects because the sign of a QTL effect only shows which parent contributed the favorable allele but not the true direction of the specific additive effect^87^. The *h* was then arbitrarily categorized as under-dominant or recessive (*h* < 0), additive (*h* = 0–0.20), partially dominant (*h* = 0.21–0.80), dominant (*h* = 0.81–1.20) and over-dominant (*h* > 1.20)^88^.

For E-QTL mapping, all possible pairs of scanning positions were tested by ICIM, since digenic interactions may be detected regardless of whether the two interacting QTLs have significant additive effects or not^85^. The probability of a marker moving into the model was set at 10^−6^ while the probability of a marker moving out of the model was set at twice the probability of a marker moving into the model^85^. The default QTL-Icimapping LOD threshold of 5.0 was used to declare the existence of E-QTLs.

### Common or consensus QTLs across five F_2_ populations

Due to differences in the individual genetic maps, it was difficult to directly find common QTLs across the five populations on the basis of the QTL or marker position in each genetic map. Therefore, QTLs obtained in each of the five individual populations were projected onto the consensus map by using either QTL peak- or flanking-marker positions indicated in the individual population genetic map using a procedure adopted from Schweizer and Stein^89^ as follows. If only peak-marker positions from the individual map were available, the QTL region was assumed by default to extend 5 cM left and right of the peak-marker position, resulting in a confidence interval of 10 cM. If only one flanking marker could be projected onto the consensus map, a QTL interval of 10 cM extension left or right from the left or right flanking marker, respectively, was assumed by default. If neither peak nor flanking markers were included in the consensus map, nearby tightly linked markers (maximum of 5 cM from the peak or flanking markers) were searched on the consensus map. If no replacement markers could be identified within this distance, the QTL was excluded from the analysis. Based on these projections, two types of common QTLs were defined. Firstly, a ‘Consensus QTL’ was defined as any region of the consensus genetic map with overlapping M-QTL intervals for a particular trait from more than one population. Secondly, a region of consensus genetic map at which M-QTL interval for one trait overlaps with that of one or more of the other traits was considered a ‘QTL Cluster’

### QTL nomenclature

For individual populations, a specific identifier was assigned to each QTL, whereby “*q*” stands for QTL, followed by a set of upper case letters indicating the trait, followed by linkage group (CcLG) name, then a hyphen, method of QTL detection, and lastly, the QTL number on that CcLG in ascending order. For example, the designation “*qPROT-cim-3*.*1*” stands for “QTL for SPC” detected using CIM on LG “CcLG03” and it is the first QTL for SPC on that CcLG. For QTLs projected onto the consensus genetic map, a prefix is added to the QTL name indicating the source population. For example “*Pop1qPROT-cim-3*.*1*” indicates a QTL for SPC from Pop1.

## Supplementary information

Supplementary Information.
