# Supplementary material for: Seed protein content and its relationships with agronomic traits in pigeonpea is controlled by both main and epistatic effects QTLs
Source: Sci Rep. 2020 Jan 14;10:214. doi: 10.1038/s41598-019-56903-z (PMC6959250; doi:10.1038/s41598-019-56903-z)

Supplementary Information

Seed protein content and its relationships with agronomic traits in pigeonpea is controlled by both main and epistatic effects QTLs

Jimmy Obala[1,2], Rachit K. Saxena[1], Vikas K. Singh[1], Sandip M. Kale[1], Vanika Garg[1], C. V. Sameer Kumar[3], K. B. Saxena[4], Pangirayi Tongoona[2], Julia Sibiya[2], Rajeev K. Varshney[1,*]

[1]International Crops Research Institute for the Semi-Arid Tropics (ICRISAT), Hyderabad, 502324, India
[2]University of KwaZulu-Natal, African Center for Crop Improvement, Scottsville, 3209 Pietermaritzburg, South Africa
[3]Professor Jayashankar Telangana State Agricultural University, Rajendranagar, Hyderabad-500030, Telangana, India
[4]Former Principal Scientist, ICRISAT, Present address: 17, NMC Housing, Al Ain, Abu Dhabi

[*]Email address for correspondence: r.k.varshney@cgiar.org

Supplementary Table S1. Sequence data generated using genotyping-by-sequencing of parental lines and F_2 individuals of five pigeonpea mapping population

Pop1 (ICP 11605 × ICP 14209)					
Sample ID	No. reads (Million)	Data (Gb)	Sample ID	No. reads (Million)	Data (Gb)
Parents			18	5.17	0.52
ICP 11605 (P_1)	1.13	0.11	19	2.95	0.30
ICP 14209 (P_2)	2.60	0.26	20	3.95	0.40
F_2			21	2.22	0.22
1	3.57	0.36	22	2.30	0.23
2	3.36	0.34	23	3.10	0.31
3	5.82	0.59	24	2.91	0.29
4	4.03	0.41	25	3.30	0.33
5	2.57	0.26	26	1.82	0.18
6	2.98	0.30	27	1.93	0.19
7	3.76	0.38	28	3.01	0.30
8	3.42	0.35	29	1.94	0.20
9	1.99	0.20	30	3.67	0.37
10	2.28	0.23	31	2.16	0.22
11	3.74	0.38	33	2.93	0.30
12	2.26	0.23	34	2.28	0.23
13	2.96	0.30	35	1.53	0.15
14	2.34	0.24	36	1.76	0.18
15	1.92	0.19	37	3.03	0.31
16	3.04	0.31	38	1.73	0.17
17	2.30	0.23	39	3.00	0.30

Supplementary Table S1 (continued)

Pop1 (ICP 11605 × ICP 14209)					
Sample ID	No. reads (Million)	Data (Gb)	Sample ID	No. reads (Million)	Data (Gb)
40	2.14	0.22	78	1.54	0.16
41	2.53	0.26	79	1.68	0.17
42	1.80	0.18	80	1.62	0.16
43	2.74	0.28	81	3.00	0.30
44	1.69	0.17	82	3.31	0.33
45	2.76	0.28	83	2.44	0.25
46	2.96	0.30	84	2.29	0.23
47	2.49	0.25	85	0.79	0.08
48	2.78	0.28	86	1.21	0.12
49	2.22	0.22	87	1.01	0.10
50	4.17	0.42	88	1.52	0.15
51	2.31	0.23	89	1.11	0.11
52	2.69	0.27	95	2.32	0.23
53	3.28	0.33	96	1.68	0.17
54	2.50	0.25	97	3.13	0.32
55	2.43	0.25	98	2.90	0.29
56	1.97	0.20	99	1.90	0.19
57	2.40	0.24	100	2.55	0.26
58	1.64	0.17	101	2.38	0.24
59	1.45	0.15	102	3.18	0.32
60	2.27	0.23	103	1.51	0.15
61	4.74	0.48	104	2.34	0.24
62	1.82	0.18	105	1.59	0.16
63	2.39	0.24	106	1.43	0.14
64	2.11	0.21	107	1.92	0.19
65	2.12	0.21	108	1.74	0.18
66	2.14	0.22	109	1.15	0.12
67	1.89	0.19	110	2.24	0.23
68	2.47	0.25	111	2.53	0.26
69	3.17	0.32	112	2.01	0.20
70	1.92	0.19	113	1.77	0.18
71	2.45	0.25	114	2.85	0.29
72	2.28	0.23	115	2.41	0.24
73	2.13	0.22	116	1.90	0.19
74	1.20	0.12	117	2.46	0.25
75	1.57	0.16	118	1.76	0.18
76	2.11	0.21	119	2.02	0.20
77	1.94	0.20	120	1.27	0.13

Supplementary Table S1 (continued)

Pop1 (ICP 11605 × ICP 14209)					
Sample ID	No. reads (Million)	Data (Gb)	Sample ID	No. reads (Million)	Data (Gb)
121	1.30	0.13	159	1.90	0.19
122	1.65	0.17	160	1.13	0.11
123	1.11	0.11	161	1.68	0.17
124	2.28	0.23	162	1.72	0.17
125	1.06	0.11	163	2.52	0.25
126	1.03	0.10	164	1.14	0.11
127	1.82	0.18	165	2.17	0.22
128	1.82	0.18	166	2.29	0.23
129	1.11	0.11	167	1.27	0.13
130	1.26	0.13	168	1.11	0.11
131	2.25	0.23	169	1.48	0.15
132	1.89	0.19	170	2.88	0.29
133	2.32	0.23	172	1.40	0.14
134	2.54	0.26	175	1.46	0.15
135	3.58	0.36	176	1.70	0.17
136	2.13	0.21	177	1.35	0.14
137	2.59	0.26	178	1.57	0.16
138	3.29	0.33	180	1.23	0.12
139	2.96	0.30	181	1.48	0.15
140	3.09	0.31	182	2.82	0.29
141	1.66	0.17	183	1.33	0.13
142	2.89	0.29	184	0.83	0.08
143	1.66	0.17	185	1.41	0.14
144	3.28	0.33	186	2.18	0.22
145	2.18	0.22	187	0.92	0.09
146	4.01	0.40	188	1.58	0.16
147	2.93	0.30	F_2 total	399.93	40.40
148	2.10	0.21	F_2 average	2.25	0.23
149	2.41	0.24			
150	2.17	0.22			
151	1.64	0.17			
152	1.19	0.12			
153	1.10	0.11			
154	2.19	0.22			
155	1.78	0.18			
156	1.57	0.16			
157	2.39	0.24			
158	3.96	0.40			

Supplementary Table S1 (continued)

Pop2 (ICP 8863 × ICP 11605)					
Sample ID	No. reads (Million)	Data (Gb)	Sample ID	No. reads (Million)	Data (Gb)
Parents			36	1.82	0.18
ICP 8863 (P_1)	3.00	0.30	37	2.32	0.23
ICP 11605 (P_2)	7.59	0.77	39	0.69	0.07
F_2			40	1.33	0.13
1	3.68	0.37	42	0.98	0.10
2	1.53	0.15	44	1.24	0.13
3	3.75	0.38	45	0.79	0.08
4	3.33	0.34	46	0.81	0.08
5	1.80	0.18	47	0.74	0.07
6	3.12	0.31	49	0.76	0.08
7	3.57	0.36	52	0.80	0.08
8	5.44	0.55	53	1.40	0.14
9	1.44	0.15	54	1.50	0.15
10	1.36	0.14	55	1.48	0.15
11	3.66	0.37	56	1.62	0.16
12	1.74	0.18	57	2.76	0.28
13	6.71	0.68	58	1.52	0.15
14	5.22	0.53	59	1.14	0.11
15	0.65	0.07	60	1.62	0.16
16	1.84	0.19	61	2.47	0.25
17	2.54	0.26	62	1.69	0.17
18	3.44	0.35	63	1.29	0.13
19	2.04	0.21	64	0.75	0.08
20	9.52	0.96	65	1.95	0.20
21	1.25	0.13	66	2.24	0.23
22	2.41	0.24	67	1.91	0.19
23	2.17	0.22	68	0.90	0.09
24	1.98	0.20	69	2.47	0.25
25	1.43	0.14	70	4.31	0.43
26	1.40	0.14	71	1.44	0.14
27	2.38	0.24	72	1.74	0.18
28	3.04	0.31	73	0.86	0.09
29	0.96	0.10	74	4.69	0.47
30	1.34	0.14	75	0.78	0.08
31	1.09	0.11	76	3.37	0.34
33	1.95	0.20	77	1.58	0.16
34	2.13	0.21	78	2.97	0.30
35	1.85	0.19	79	0.86	0.09

Supplementary Table S1 (continued)

Pop2 (ICP 8863 × ICP 11605)					
Sample ID	No. reads (Million)	Data (Gb)	Sample ID	No. reads (Million)	Data (Gb)
80	1.15	0.12	119	3.80	0.38
81	4.03	0.41	121	1.53	0.15
82	2.58	0.26	122	3.23	0.33
83	1.04	0.11	123	1.98	0.20
84	0.70	0.07	124	2.98	0.30
85	0.56	0.06	125	2.74	0.28
86	0.76	0.08	126	2.19	0.22
87	0.68	0.07	127	3.94	0.40
88	1.57	0.16	128	3.99	0.40
89	0.86	0.09	129	3.93	0.40
90	0.63	0.06	130	3.93	0.40
91	1.44	0.15	131	4.75	0.48
92	1.21	0.12	132	3.65	0.37
93	0.79	0.08	133	1.63	0.16
94	1.22	0.12	135	1.97	0.20
95	2.38	0.24	136	2.52	0.25
96	2.21	0.22	137	1.94	0.20
97	3.77	0.38	138	2.14	0.22
98	3.42	0.34	139	1.37	0.14
99	0.95	0.10	140	2.30	0.23
100	3.19	0.32	141	1.89	0.19
101	1.74	0.18	142	1.39	0.14
102	4.22	0.43	143	1.55	0.16
103	2.28	0.23	144	1.62	0.16
104	1.46	0.15	145	0.94	0.09
105	2.90	0.29	146	0.85	0.09
106	1.13	0.11	147	2.03	0.20
107	1.77	0.18	148	1.26	0.13
108	1.48	0.15	149	0.92	0.09
109	1.13	0.11	150	0.87	0.09
110	0.75	0.08	152	0.83	0.08
111	1.37	0.14	154	1.14	0.12
112	1.36	0.14	155	0.90	0.09
113	1.01	0.10	156	2.23	0.22
114	1.09	0.11	157	0.74	0.07
115	1.41	0.14	159	1.04	0.11
117	6.78	0.68	160	1.34	0.14
118	0.72	0.07	161	1.90	0.19

Supplementary Table S1 (continued)

Pop2 (ICP 8863 × ICP 11605)		
Sample ID	No. reads (Million)	Data (Gb)
162	1.03	0.10
163	1.48	0.15
164	1.25	0.13
165	2.12	0.21
166	2.25	0.23
167	1.28	0.13
168	0.94	0.09
169	1.26	0.13
170	1.68	0.17
171	0.73	0.07
172	0.94	0.09
173	0.92	0.09
174	1.09	0.11
175	1.89	0.19
176	1.16	0.12
177	0.68	0.07
178	1.43	0.14
179	0.58	0.06
180	0.63	0.06
181	0.61	0.06
182	0.63	0.06
183	0.87	0.09
184	0.49	0.05
185	0.98	0.10
186	1.09	0.11
187	0.83	0.08
188	0.78	0.08
F_2 total	332.68	34.67
F_2 average	1.90	0.20

Supplementary Table S1 (continued)

Pop3 (HPL 24 × ICP 11605)					
Sample ID	No. reads (Million)	Data (Gb)	Sample ID	No. reads (Million)	Data (Gb)
Parents			54	1.32	0.13
HPL 24	2.96	0.30	55	2.62	0.26
ICP 11605	3.31	0.33	56	1.20	0.12
F_2			57	4.88	0.49
2	1.04	0.10	58	1.00	0.10
3	1.29	0.13	59	2.01	0.20
7	4.36	0.44	60	1.64	0.17
8	0.85	0.09	61	1.92	0.19
9	1.96	0.20	62	1.10	0.11
11	1.90	0.19	63	0.80	0.08
13	0.89	0.09	64	0.92	0.09
16	3.65	0.37	65	0.92	0.09
17	1.08	0.11	66	2.66	0.27
18	2.97	0.30	67	1.65	0.17
20	1.08	0.11	69	1.16	0.12
21	1.80	0.18	71	1.45	0.15
22	0.79	0.08	73	2.80	0.28
23	2.29	0.23	74	3.03	0.31
24	0.86	0.09	75	2.57	0.26
25	4.75	0.48	76	1.27	0.13
26	0.81	0.08	77	1.31	0.13
27	1.95	0.20	78	0.97	0.10
31	1.24	0.12	79	3.45	0.35
32	1.03	0.10	80	1.02	0.10
33	3.82	0.39	83	1.13	0.11
34	2.28	0.23	84	1.02	0.10
35	2.07	0.21	85	2.53	0.26
37	0.87	0.09	86	4.84	0.49
39	5.04	0.51	87	2.20	0.22
40	6.84	0.69	88	1.37	0.14
42	1.53	0.15	89	2.58	0.26
43	1.15	0.12	90	3.73	0.38
45	1.10	0.11	91	1.28	0.13
47	3.08	0.31	92	2.57	0.26
49	1.96	0.20	93	3.12	0.32
51	1.36	0.14	94	3.75	0.38
52	1.84	0.19	95	3.45	0.35
53	1.44	0.15	96	2.95	0.30

Supplementary Table S1 (continued)

Pop3 (HPL 24 × ICP 11605)					
Sample ID	No. reads (Million)	Data (Gb)	Sample ID	No. reads (Million)	Data (Gb)
97	4.42	0.45	140	1.52	0.15
98	4.99	0.50	141	0.80	0.08
99	1.13	0.11	142	1.54	0.16
100	1.80	0.18	144	3.40	0.34
101	2.75	0.28	145	1.73	0.18
102	2.62	0.26	146	1.74	0.18
103	2.42	0.24	147	1.17	0.12
104	2.61	0.26	148	1.23	0.12
105	3.13	0.32	149	3.12	0.32
106	1.79	0.18	150	2.04	0.21
107	3.14	0.32	151	1.80	0.18
108	1.79	0.18	152	1.64	0.17
109	1.26	0.13	153	2.17	0.22
110	2.40	0.24	154	2.52	0.25
111	3.17	0.32	155	2.47	0.25
112	3.18	0.32	156	3.05	0.31
113	1.33	0.13	157	2.47	0.25
114	1.87	0.19	158	1.80	0.18
115	1.26	0.13	159	2.64	0.27
117	1.44	0.15	160	1.86	0.19
119	1.83	0.18	161	2.01	0.20
122	2.25	0.23	162	3.07	0.31
124	1.85	0.19	163	3.07	0.31
125	0.89	0.09	164	2.34	0.24
126	1.64	0.17	165	2.79	0.28
127	2.02	0.20	166	2.56	0.26
128	2.21	0.22	167	4.54	0.46
129	1.08	0.11	168	2.01	0.20
130	1.33	0.13	169	2.65	0.27
131	1.26	0.13	170	2.63	0.27
132	2.06	0.21	171	1.91	0.19
133	1.42	0.14	172	1.96	0.20
134	2.72	0.27	173	2.52	0.25
135	1.80	0.18	174	3.71	0.37
136	2.19	0.22	175	2.68	0.27
137	1.63	0.16	176	3.38	0.34
138	2.54	0.26	177	2.20	0.22
139	1.75	0.18	178	2.45	0.25

Supplementary Table S1 (continued)

Pop3 (HPL 24 × ICP 11605)		
Sample ID	No. reads (Million)	Data (Gb)
179	1.01	0.10
180	1.35	0.14
181	2.49	0.25
182	1.70	0.17
183	1.33	0.13
185	0.96	0.10
186	1.29	0.13
187	0.97	0.10
188	3.10	0.31
F_2 total	335.52	33.89
F_2 average	2.14	0.22

Pop4 (ICP 8863 × ICPL 87119)					
Sample ID	No. reads (Million)	Data (Gb)	Sample ID	No. reads (Million)	Data (Gb)
Parents			26	2.21	0.22
ICP 8863	2.56	0.26	27	1.13	0.11
ICPL 87119 (Reference genome sequence)			28	1.34	0.14
F_2			29	0.84	0.08
1	6.45	0.65	30	1.08	0.11
3	2.71	0.27	31	0.84	0.08
4	6.48	0.65	32	1.58	0.16
7	1.17	0.12	33	1.14	0.11
8	1.66	0.17	35	1.83	0.18
9	1.75	0.18	36	1.40	0.14
11	1.07	0.11	37	1.14	0.12
12	1.31	0.13	38	1.47	0.15
13	3.33	0.34	41	2.20	0.22
14	1.10	0.11	45	0.94	0.10
15	0.89	0.09	47	1.69	0.17
16	1.20	0.12	53	1.67	0.17
18	1.00	0.10	54	1.02	0.10
19	1.73	0.18	55	1.01	0.10
20	1.94	0.20	56	2.10	0.21
22	1.52	0.15	57	1.50	0.15
24	1.76	0.18	58	2.14	0.22
25	1.38	0.14	60	0.97	0.10

Supplementary Table S1 (continued)

Pop4 (ICP 8863 × ICPL 87119)					
Sample ID	No. reads (Million)	Data (Gb)	Sample ID	No. reads (Million)	Data (Gb)
61	1.03	0.10	120	1.58	0.16
62	2.04	0.21	122	1.35	0.14
63	1.19	0.12	123	6.32	0.64
64	3.29	0.33	124	1.35	0.14
65	1.14	0.12	125	7.65	0.77
66	1.23	0.12	126	1.53	0.15
68	1.01	0.10	127	0.85	0.09
69	1.42	0.14	128	1.27	0.13
70	0.97	0.10	130	1.35	0.14
71	1.59	0.16	131	1.64	0.17
72	4.24	0.43	132	1.68	0.17
73	2.51	0.25	136	1.04	0.11
74	0.97	0.10	137	1.03	0.10
75	1.31	0.13	141	1.16	0.12
76	1.06	0.11	142	0.89	0.09
77	1.10	0.11	145	0.91	0.09
78	1.54	0.16	146	0.98	0.10
80	1.80	0.18	147	1.22	0.12
81	0.88	0.09	148	1.21	0.12
82	1.10	0.11	149	2.36	0.24
83	0.93	0.09	150	1.71	0.17
84	0.91	0.09	151	1.07	0.11
87	1.73	0.17	152	5.88	0.59
88	2.19	0.22	153	1.33	0.13
92	1.07	0.11	154	4.05	0.41
93	2.36	0.24	155	2.09	0.21
94	1.20	0.12	157	2.74	0.28
101	1.21	0.12	158	2.06	0.21
102	2.58	0.26	159	7.21	0.73
103	2.18	0.22	162	2.14	0.22
105	1.12	0.11	163	0.94	0.10
106	2.60	0.26	165	1.34	0.14
108	1.75	0.18	166	0.92	0.09
109	1.46	0.15	167	1.86	0.19
110	1.66	0.17	168	0.87	0.09
112	0.88	0.09	171	1.59	0.16
117	2.41	0.24	172	1.46	0.15
118	1.57	0.16	173	1.25	0.13

Supplementary Table S1 (continued)

Pop4 (ICP 8863 × ICPL 87119)		
Sample ID	No. reads (Million)	Data (Gb)
174	5.19	0.52
175	6.33	0.64
177	3.70	0.37
178	1.33	0.13
179	3.85	0.39
181	3.23	0.33
182	0.96	0.10
184	0.88	0.09
187	1.03	0.10
218	3.19	0.32
220	8.19	0.83
221	1.59	0.16
222	5.33	0.54
223	2.62	0.26
224	4.22	0.43
225	3.42	0.35
226	2.11	0.21
227	3.82	0.39
229	5.47	0.55
231	2.81	0.28
232	4.18	0.42
F_2 total	284.77	28.76
F_2 average	2.06	0.21

Supplementary Table S1 (continued)

Pop5 (ICP 5529 × ICP 11605)					
Sample ID	No. reads (Million)	Data (Gb)	Sample ID	No. reads (Million)	Data (Gb)
Parents			37	1.29	0.13
ICP 5529	5.37	0.54	38	0.64	0.06
ICP 11605	1.61	0.16	39	1.09	0.11
F_2			40	1.28	0.13
2	2.25	0.23	41	2.43	0.25
3	0.97	0.10	42	0.89	0.09
4	1.82	0.18	43	2.19	0.22
6	3.40	0.34	44	0.91	0.09
7	1.91	0.19	45	0.86	0.09
8	5.26	0.53	46	0.96	0.10
9	2.57	0.26	48	2.22	0.22
10	1.00	0.10	49	2.66	0.27
12	1.43	0.14	50	2.02	0.20
13	1.14	0.12	52	0.71	0.07
14	1.97	0.20	53	0.96	0.10
15	0.62	0.06	54	0.64	0.06
16	1.00	0.10	55	0.80	0.08
17	1.33	0.13	56	0.99	0.10
18	0.77	0.08	57	3.05	0.31
19	2.59	0.26	58	1.30	0.13
20	0.87	0.09	59	2.02	0.20
21	0.68	0.07	60	2.04	0.21
22	1.02	0.10	61	1.92	0.19
23	4.11	0.41	62	1.73	0.17
24	1.82	0.18	63	1.05	0.11
25	1.98	0.20	64	1.26	0.13
26	1.22	0.12	65	3.57	0.36
27	0.96	0.10	66	2.52	0.25
28	2.82	0.28	67	4.01	0.41
29	0.67	0.07	68	1.46	0.15
30	1.15	0.12	69	2.18	0.22
31	1.26	0.13	70	2.20	0.22
32	0.72	0.07	71	1.19	0.12
33	0.89	0.09	72	1.83	0.18
35	1.94	0.20	73	2.04	0.21
36	1.58	0.16	74	2.26	0.23

Supplementary Table S1 (continued)

Pop5 (ICP 5529 × ICP 11605)					
Sample ID	No. reads (Million)	Data (Gb)	Sample ID	No. reads (Million)	Data (Gb)
75	2.70	0.27	114	2.52	0.25
76	1.91	0.19	115	1.17	0.12
77	3.47	0.35	116	0.64	0.07
78	1.89	0.19	117	1.68	0.17
79	2.06	0.21	118	0.51	0.05
80	2.14	0.22	119	0.91	0.09
81	4.08	0.41	120	1.13	0.11
82	1.43	0.14	121	1.56	0.16
83	2.48	0.25	122	1.62	0.16
84	3.11	0.31	123	1.21	0.12
85	0.92	0.09	124	1.91	0.19
86	1.64	0.17	125	0.96	0.10
87	1.89	0.19	126	0.52	0.05
89	1.24	0.12	127	2.93	0.30
90	1.22	0.12	128	1.06	0.11
91	1.37	0.14	129	0.91	0.09
92	1.18	0.12	130	2.32	0.23
93	2.90	0.29	131	1.33	0.13
94	1.92	0.19	132	1.32	0.13
95	1.31	0.13	133	1.00	0.10
96	1.23	0.12	134	0.59	0.06
97	1.91	0.19	135	1.16	0.12
98	1.39	0.14	136	1.43	0.14
99	0.64	0.06	137	2.05	0.21
100	1.06	0.11	138	2.05	0.21
101	0.58	0.06	139	1.53	0.15
102	0.76	0.08	140	1.23	0.12
103	0.74	0.07	142	0.66	0.07
104	0.70	0.07	143	1.29	0.13
105	1.89	0.19	144	0.89	0.09
106	1.04	0.11	145	0.80	0.08
107	0.79	0.08	146	1.65	0.17
108	0.47	0.05	147	1.26	0.13
109	0.41	0.04	148	1.12	0.11
111	0.83	0.08	149	0.74	0.07
112	1.44	0.15	150	1.70	0.17
113	1.61	0.16	151	1.99	0.20

Supplementary Table S1 (continued)

Pop5 (ICP 5529 × ICP 11605)		
Sample ID	No. reads (Million)	Data (Gb)
152	1.56	0.16
153	1.87	0.19
154	2.18	0.22
155	2.81	0.28
156	1.90	0.19
157	2.28	0.23
158	2.73	0.28
159	1.27	0.13
160	2.25	0.23
161	2.04	0.21
162	1.38	0.14
163	3.36	0.34
164	1.86	0.19
165	3.77	0.38
166	2.36	0.24
167	3.08	0.31
168	2.32	0.23
169	1.55	0.16
170	1.98	0.20
171	1.83	0.19
172	2.36	0.24
173	2.96	0.30
174	1.53	0.15
175	3.02	0.31
176	1.95	0.20
177	1.63	0.16
178	2.35	0.24
179	2.11	0.21
180	2.65	0.27
181	2.13	0.22
182	0.69	0.07
183	2.16	0.22
184	0.62	0.06
185	0.92	0.09
186	0.61	0.06
187	1.96	0.20
188	1.94	0.20
F_2 total	298.56	30.15
F_2 average	1.67	0.17

Supplementary Table S2. Main effect QTLs for seed protein content and agronomic traits identified using composite interval mapping (CIM) and inclusive composite interval mapping (ICIM) in five pigeonpea F_2 mapping populations

QTL name	Position	Flanking markers	QTL interval	LOD	PVE (%)	*a*	*d*	*d*/|*a*|	Gene action
**Pop1 (ICP 11605 × ICP 14209)**									
Seed protein content									
qPROT-cim-3.1	81.4	S3_12113347 - S3_21274904	1.7	2.8	7.8	0.4	-0.2	-0.50	recessive
qPROT-cim-3.2	89.7	S3_23699007 - S3_18226407	2.6	2.6	10.3	-0.5	0.4	0.80	dominant
qPROT-icim-3.1	99.0	S3_25565937 - S3_16199983	0.4	2.7	8.6	-0.5	0.4	0.80	dominant
qPROT-cim-4.1	32.5	S4_1586438 - S4_5314034	5.1	2.9	9.0	-0.3	0.5	1.67	over-dominant
qPROT-cim-11.1	43.0	S11_11249294 - S11_9768899	2.5	3.5	16.6	-0.6	0.5	0.83	dominant
qPROT-cim-11.2	170.1	S11_20646423 - S11_24857528	1.4	3.8	13.8	-0.5	0.3	0.60	partially dominant
100-seed weight									
qSW-icim-1.1	15.0	S1_4374250 - S1_4757283	4.8	3.0	6.6	-0.3	0.4	1.57	over dominant
qSW-icim-4.1	74.0	S4_4734626 - S4_9854357	19.4	3.9	7.4	-0.3	-0.4	-1.49	recessive
qSW-cim-6.1	16.7	S6_14311667 - S6_14311546	4.1	3.0	4.7	-0.3	-0.2	-0.47	recessive
qSW-cim-8.1	1.0	S8_5787667 - S8_11399561	15.5	2.6	10.6	0.4	-0.3	-0.92	recessive
qSW-cim-8.2	100.3	S8_13310192 - S8_4675310	2.1	4.3	12.3	0.4	-0.3	-0.82	recessive
qSW-icim-8.1	91.0	S8_7083582 - S8_6388803	1.6	6.8	15.0	0.5	-0.1	-0.25	recessive
qSW-icim-10.1	60.0	S10_1984771 - S10_1397376	2.5	2.9	6.4	0.1	0.6	5.27	over dominant
qSW-cim-11.1	102.3	S11_23359277 - S11_10698003	0.2	2.6	3.6	-0.4	-0.2	-0.40	recessive
Seed yield									
qSY-cim-3.1	130.0	S3_14273504 - S3_24137311	1.4	2.6	6.1	-0.8	22.0	27.21	over dominant
qSY-cim-3.2	135.6	S3_8561649 - S3_8569720	5.2	3.0	5.2	-12.2	-3.7	-0.30	recessive
qSY-icim-3.1	135.0	S3_8561649 - S3_8569720	5.2	4.5	10.2	-14.5	-4.6	-0.32	recessive
qSY-cim-5.1	14.2	S5_4692888 - S5_4692888	0.0	2.5	5.6	1.0	-16.3	-16.64	recessive
qSY-cim-10.1	53.5	S10_4202839 - S10_1984771	8.6	3.0	15.4	12.5	-16.9	-1.36	recessive
qSY-cim-11.1	49.2	S11_46091155 - S11_33138038	1.5	2.6	7.5	-6.4	18.7	2.92	over dominant
qSY-cim-11.2	152.1	S11_38654219 - S11_11722207	0.7	2.8	9.6	-11.2	7.3	0.65	partially dominant
Growth habit									

QTL name	Position	Flanking markers	QTL interval	LOD	PVE (%)	*a*	*d*	*d*/|*a*|	Gene action
qGH-cim-3.1	56.2	S3_28498782 - S3_21310513	2.7	3.2	10.9	0.15	-0.08	-0.53	recessive
qGH-cim-3.2	65.6	S3_17441541 - S3_21244595	1.6	13.9	91.3	0.32	-0.30	-0.95	recessive
qGH-icim-3.1	66.0	S3_17441541 - S3_21244595	1.6	17.3	41.3	0.32	-0.29	-0.91	recessive
qGH-cim-3.3	78.5	S3_14813065 - S3_14778845	4.2	4.1	12.9	0.13	-0.13	-0.97	recessive
qGH-icim-3.2	79.0	S3_14778845 - S3_14778845	1.0	2.9	8.6	0.11	-0.18	-1.65	recessive
qGH-icim-11.1	183.0	S11_24857409 - S11_47336921	1.2	3.2	6.1	-0.11	0.05	0.46	partially dominant
Days to first flowering									
qDFF-cim-3.1	56.2	S3_28498782 - S3_21310513	2.7	5.4	14.5	-6.0	0.6	0.10	additive
qDFF-cim-3.2	66.3	S3_21244595 - S3_28538775	2.4	9.5	20.3	-8.4	0.5	0.06	additive
qDFF-icim-3.1	67.0	S3_21244595 - S3_28538775	2.4	11.1	25.4	-9.4	2.8	0.30	partially dominant
qDFF-cim-3.3	75.5	S3_14813065 - S3_14778845	4.2	3.1	7.4	-4.4	-0.3	-0.06	recessive
qDFF-cim-3.4	91.0	S3_18226407 - S3_5582712	0.8	2.9	5.7	4.3	-0.4	-0.09	recessive
qDFF-cim-11.1	124.2	S11_39257442 - S11_29670540	0.9	2.5	6.5	-2.3	5.7	2.44	over dominant
qDFF-cim-11.2	137.2	S11_28741859 - S11_20235498	0.9	2.9	7.2	4.0	-1.7	-0.43	recessive
**Pop2 (ICP 8863 x ICP 11605)**									
Seed protein content									
qPROT-cim-1.1	20.2	S1_15372966 - S1_5944791	1.1	2.6	7.7	0.4	-0.3	-0.75	recessive
qPROT-icim-3.1	2.0	S3_22234078 - S3_19578263	2.2	2.7	6.9	0.3	-0.5	-1.67	recessive
qPROT-cim-3.1	55.9	S3_17193829 - S3_14758073	11.8	3.8	12.8	0.7	-0.3	-0.43	recessive
qPROT-icim-3.2	56.0	S3_17193829 - S3_14758073	11.8	2.9	7.4	0.6	-0.3	-0.50	recessive
qPROT-cim-9.1	67.4	S9_10003418 - S9_10229309	1.4	3.6	5.7	0.1	-0.8	-8.00	recessive
qPROT-icim-11.1	46.0	S11_2019429 - S11_22353396	2.0	2.7	12.3	-0.7	0.5	0.71	partially dominant
qPROT-icim-11.2	126.0	S11_21940736 - S11_30337876	1.0	2.9	9.4	0.6	0.4	0.67	partially dominant
qPROT-cim-11.1	126.1	S11_30337876 - S11_45761666	1.6	3.4	0.7	0.4	0.5	1.25	over-dominant
100-seed weight									
qSW-cim-1.1	7.4	S1_4759267 - S1_15329865	4.6	4.6	6.1	0.6	0.2	0.36	partially dominant
qSW-cim-1.2	22.2	S1_5944791 - S1_9033631	9.9	3.4	7.5	0.7	0.2	0.26	partially dominant
qSW-icim-1.1	22.0	S1_5944791 - S1_9033631	9.9	9.9	29.1	1.0	0.2	0.20	additive

QTL name	Position	Flanking markers	QTL interval	LOD	PVE (%)	*a*	*d*	*d*/|*a*|	Gene action
qSW-icim-11.1	165.0	S11_41096347 - S11_44938548	1.3	2.7	8.4	0.5	-0.5	-0.91	recessive
Seed yield									
qSY-cim-1.1	9.4	S1_4759267 - S1_15329865	4.6	2.9	7.1	-14.0	-5.0	-0.36	recessive
qSY-icim-1.1	36.0	S1_12899653 - S1_11050274	3.2	4.8	9.8	-11.3	-7.0	-0.62	recessive
qSY-icim-2.1	171.0	S2_13394818 - S2_26969919	2.3	3.5	7.3	2.2	14.8	6.77	over dominant
qSY-icim-3.1	2.0	S3_22234078 - S3_19578263	2.2	8.0	16.0	-1.6	21.1	13.50	over dominant
qSY-cim-3.1	3.2	S3_19578263 - S3_21274904	3.8	5.3	11.8	-2.0	21.0	10.56	over dominant
qSY-icim-6.1	69.0	S6_18322776 - S6_18322873	3.1	5.7	9.4	-10.4	2.1	0.20	additive
qSY-icim-7.1	71.0	S7_6897487 - S7_14683829	11.5	3.6	5.9	9.2	3.3	0.36	partially dominant
qSY-cim-11.1	28.8	S11_9114357 - S11_4453854	5.2	3.0	10.6	9.8	-22.6	-2.30	recessive
qSY-icim-11.1	31.0	S11_9114357 - S11_4453854	5.2	4.6	11.3	4.6	-18.5	-4.00	recessive
qSY-cim-11.2	125.1	S11_21940736 - S11_30337876	1.0	4.1	1.7	-9.2	-10.2	-1.11	recessive
Growth habit									
qGH-cim-2.1	139.1	S2_32727778 - S2_13394806	0.6	2.7	4.0	-0.1	0.0	-0.05	recessive
qGH-cim-3.1	2.0	S3_22234078 - S3_19578263	2.2	4.9	13.1	0.1	-0.2	-1.23	recessive
qGH-cim-3.2	9.0	S3_21274904 - S3_28538775	6.6	8.2	37.0	0.2	-0.4	-1.66	recessive
qGH-icim-3.1	20.0	S3_28933239 - S3_21244595	6.8	12.4	23.9	0.2	-0.3	-1.31	recessive
qGH-cim-3.3	22.7	S3_21244595 - S3_18933167	7.6	14.0	36.6	0.2	-0.3	-1.28	recessive
qGH-icim-3.2	43.0	S3_18929445 - S3_18929378	5.1	15.0	25.4	0.2	-0.2	-0.96	recessive
qGH-cim-3.4	51.4	S3_18929378 - S3_17193829	8.5	9.8	64.7	0.2	-0.4	-1.65	recessive
Days to first flowering									
qDFF-icim-2.1	174.0	S2_6037470 - S2_6037490	0.6	2.6	4.5	4.3	-4.0	-0.91	recessive
qDFF-cim-3.1	1.0	S3_22234078 - S3_19578263	2.2	4.0	16.0	-3.9	5.6	1.44	over dominant
qDFF-cim-3.2	25.7	S3_21244595 - S3_18933167	7.6	6.2	19.1	-5.5	2.4	0.44	partially dominant
qDFF-cim-3.3	41.3	S3_18929445 - S3_18929378	5.1	9.9	28.2	-6.5	3.6	0.56	partially dominant
qDFF-icim-3.1	42.0	S3_18929445 - S3_18929378	5.1	11.7	26.6	-7.8	1.5	0.19	additive
qDFF-cim-11.1	56.3	S11_42065843 - S11_26230931	2.3	2.9	4.0	-4.4	-3.2	-0.74	recessive
qDFF-cim-11.2	128.3	S11_45761666 - S11_18137395	0.6	3.0	8.1	2.9	-3.6	-1.23	recessive

QTL name	Position	Flanking markers	QTL interval	LOD	PVE (%)	*a*	*d*	*d*/|*a*|	Gene action
qDFF-cim-11.3	136.2	S11_26422066 - S11_32080647	2.2	3.4	36.3	5.9	-6.2	-1.06	recessive
qDFF-icim-11.1	137.0	S11_26422066 - S11_32080647	2.2	3.1	7.9	2.9	-5.7	-1.98	recessive
**Pop3 (HPL 24 x ICP 11605)**									
Seed protein content									
qPROT-icim-2.1	20.0	S2_17395609 - S2_17836619	5.7	3.7	7.9	0.6	-0.1	-0.17	recessive
qPROT-cim-2.1	28.6	S2_18621223 - S2_5077845	4.1	4.1	23.5	0.9	-0.6	-0.67	recessive
qPROT-icim-2.2	67.0	S2_17642300 - S2_27324059	1.6	3.4	10.0	0.8	0.0	0.00	additive
qPROT-cim-2.2	67.5	S2_27324059 - S2_27324056	0.1	3.1	6.0	0.6	0.0	0.00	additive
qPROT-icim-3.1	10.0	S3_28538775 - S3_21274904	10.0	3.0	5.8	-0.6	-0.1	-0.17	recessive
qPROT-cim-3.1	40.8	S3_17145449 - S3_18154873	3.4	4.4	4.1	-0.6	-0.5	-0.83	recessive
qPROT-cim-3.2	46.7	S3_18154848 - S3_17193829	3.4	4.6	3.8	-0.6	-0.6	-1.00	recessive
qPROT-cim-3.3	55.1	S3_18154875 - S3_14813065	4.7	3.2	5.6	-0.7	-0.2	-0.29	recessive
qPROT-icim-3.2	102.0	S3_6531705 - S3_24127268	1.8	2.5	6.7	-0.4	-0.6	-1.50	recessive
qPROT-icim-6.1	75.0	S6_14548839 - S6_6094182	7.0	3.8	9.2	0.6	-0.5	-0.83	recessive
qPROT-icim-10.1	21.0	S10_6745618 - S10_18754549	20.7	2.8	5.1	-0.2	-0.7	-3.50	recessive
qPROT-cim-11.1	82.6	S11_17781645 - S11_39391791	0.3	3.0	7.4	0.4	-0.6	-1.50	recessive
qPROT-icim-11.1	119.0	S11_45315652 - S11_32081128	0.7	2.7	9.5	0.7	0.7	1.00	dominant
qPROT-icim-11.2	123.0	S11_7540489 - S11_21960241	0.4	4.2	8.5	0.8	1.0	1.25	over-dominant
100-seed weight									
qSW-cim-1.1	43.9	S1_11314974 - S1_4759267	1.4	9.8	44.4	-0.9	0.6	0.59	partially dominant
qSW-cim-1.2	53.8	S1_9401795 - S1_5944791	1.6	13.6	46.6	-1.0	0.6	0.55	partially dominant
qSW-icim-1.1	54.0	S1_5944791 - S1_5441596	0.8	13.7	35.7	-1.2	0.4	0.36	partially dominant
qSW-cim-1.3	66.9	S1_15394802 - S1_6218143	4.2	4.8	10.3	-0.6	-0.1	-0.16	recessive
qSW-cim-5.1	3.0	S5_3437907 - S5_3437906	3.2	2.5	5.4	-0.4	0.1	0.27	partially dominant
qSW-icim-8.1	6.0	S8_388862 - S8_648217	3.1	7.7	16.3	-0.7	0.1	0.18	additive
qSW-cim-8.1	7.0	S8_388862 - S8_648217	3.1	7.4	16.0	-0.7	0.1	0.11	additive
Seed yield									
qSY-icim-1.1	92.0	S1_12652912 - S1_556023	2.2	2.8	9.1	-7.8	-11.4	-1.46	recessive

QTL name	Position	Flanking markers	QTL interval	LOD	PVE (%)	*a*	*d*	*d*/|*a*|	Gene action
qSY-icim-2.1	106.0	S2_33896199 -S2_36167974	1.1	2.5	4.5	4.1	-9.2	-2.27	recessive
qSY-cim-2.1	106.0	S2_33896199 -S2_36167974	1.1	3.6	16.0	9.2	-9.7	-1.06	recessive
qSY-cim-2.2	152.4	S2_19392910 - S2_6254553	6.1	3.3	14.4	6.4	-13.5	-2.10	recessive
qSY-cim-2.3	210.8	S2_22210488 - S2_3910532	5.1	2.7	8.9	-0.8	-14.7	-17.30	recessive
qSY-icim-3.1	48.0	S3_18154848 - S3_17193829	3.4	3.6	7.1	1.0	11.3	11.64	over dominant
qSY-icim-3.2	117.0	S3_8766429 - S3_18226407	1.0	2.7	7.7	5.7	-10.6	-1.87	recessive
qSY-icim-4.1	14.0	S4_3592410 - S4_2761907	16.1	6.5	20.3	-1.8	20.4	11.27	over dominant
qSY-cim-4.1	16.1	S4_3592410 - S4_2761907	16.1	5.4	5.9	-2.3	16.7	7.18	over dominant
qSY-cim-10.1	63.8	S10_22177883 - S10_12463946	4.0	3.4	40.2	5.2	-22.6	-4.38	recessive
qSY-cim-11.1	68.4	S11_36998432 - S11_36724203	1.2	2.6	8.6	8.2	-4.0	-0.49	recessive
qSY-icim-11.1	82.0	S11_10013709 - S11_6081367	0.5	3.1	9.2	10.2	-10.7	-1.05	recessive
qSY-cim-11.2	82.5	S11_6081367 - S11_45330880	0.1	3.0	11.3	6.6	-7.2	-1.09	recessive
qSY-cim-11.3	128.0	S11_45580349 - S11_24800852	0.6	2.5	4.8	-0.2	-11.3	-53.08	recessive
Growth habit									
qGH-cim-3.1	14.7	S3_21274904 - S3_21244595	4.7	25.3	6.4	-0.4	-0.4	-0.96	recessive
qGH-icim-3.1	16.0	S3_21244595 - S3_22913898	6.9	31.4	54.4	-0.4	-0.4	-1.21	recessive
qGH-cim-3.2	22.1	S3_19132565 - S3_25402258	0.4	20.3	6.5	-0.4	-0.4	-1.10	recessive
qGH-icim-3.2	37.0	S3_17628375 - S3_17145449	6.3	10.8	14.3	-0.2	-0.1	-0.64	recessive
qGH-cim-3.3	46.7	S3_18154848 - S3_17193829	3.4	13.1	13.3	-0.3	-0.4	-1.62	recessive
qGH-cim-3.4	55.1	S3_18154875 - S3_14813065	4.7	7.8	5.8	-0.1	-0.4	-3.03	recessive
qGH-icim-4.1	0.0	S4_3592410 - S4_2761907	16.1	3.6	4.1	0.0	0.2	5.34	over dominant
qGH-icim-8.1	119.0	S8_15841326 - S8_14899494	3.1	3.0	4.0	0.0	-0.2	-6.71	recessive
qGH-cim-9.1	21.2	S9_10003418 - S9_8998000	4.7	3.0	5.2	0.1	-0.2	-3.22	recessive
Days to first flowering									
qDFF-icim-2.1	90.0	S2_23123517 - S2_34204720	1.1	2.9	3.4	-3.8	-0.7	-0.19	recessive
qDFF-icim-2.2	188.0	S2_3012518 - S2_36010450	8.8	2.7	4.1	1.8	-5.9	-3.34	recessive
qDFF-icim-3.1	17.0	S3_21244595 - S3_22913898	6.9	9.1	14.7	9.2	1.7	0.18	additive
qDFF-cim-3.1	24.4	S3_25402258 - S3_18933167	3.0	16.0	13.2	13.9	12.2	0.88	dominant

QTL name	Position	Flanking markers	QTL interval	LOD	PVE (%)	*a*	*d*	*d*/|*a*|	Gene action
qDFF-icim-3.2	36.0	S3_17628375 - S3_17145449	6.3	20.0	31.9	8.9	11.0	1.24	over dominant
qDFF-cim-3.2	46.7	S3_18154848 - S3_17193829	3.4	6.9	40.3	3.8	11.1	2.92	over dominant
qDFF-cim-3.3	54.1	S3_18154875 - S3_14813065	4.7	4.4	39.7	-1.8	11.3	6.28	over dominant
qDFF-icim-3.3	123.0	S3_8040710 - S3_23698867	2.5	6.7	8.4	6.7	-2.2	-0.32	recessive
qDFF-icim-11.1	22.0	S11_27958079 - S11_24152343	0.2	2.6	4.8	0.6	6.7	11.71	over dominant
**Pop4 (ICP 8863 x ICPL 87119)**									
Seed protein content									
qPROT-cim-2.1	41.6	S2_16460899 - S2_2144739	1.3	3.0	1.7	0.3	0.5	1.67	overdominant
qPROT-icim-2.1	42.0	S2_16460899 - S2_2144739	1.3	2.7	8.5	0.4	0.5	1.25	overdominant
qPROT-icim-2.2	178.0	S2_9426717 - S2_24073225	2.2	7.5	18.9	0.2	1.3	6.50	overdominant
qPROT-icim-6.1	83.0	S6_20608121 - S6_12302413	5.3	4.3	10.6	0.1	-0.8	-8.00	recessive
qPROT-cim-6.1	83.8	S6_12302413 - S6_1292942	4.4	4.2	16.3	0.5	-0.6	-1.20	recessive
qPROT-cim-6.2	154.4	S6_11344426 - S6_1641606	7.3	3.3	8.3	-0.1	0.8	8.00	overdominant
qPROT-cim-7.1	122.7	S7_462935 - S7_1601723	1.6	3.3	11.3	-0.2	0.9	4.50	overdominant
qPROT-cim-8.1	61.6	S8_1988786 - S8_19001660	1.9	2.5	6.7	0.2	-0.7	-3.50	recessive
qPROT-icim-8.1	70.0	S8_9578163 -S8_4817489	0.9	3.8	8.2	-0.1	0.7	7.00	overdominant
qPROT-cim-10.1	95.8	S10_17537652 - S10_632595	3.2	2.6	1.7	0.4	1.1	2.75	overdominant
qPROT-cim-11.1	2.0	S11_38211354- S11_36725317	5.4	3.0	8.1	-0.2	0.9	4.50	overdominant
qPROT-cim-11.2	84.4	S11_20139207 - S11_25774244	0.2	4.1	9.1	-0.1	0.9	9.00	overdominant
qPROT-cim-11.3	100.4	S11_24067221 - S11_26654392	0.2	2.6	1.8	0.2	0.7	3.50	overdominant
qPROT-cim-11.4	137.2	S11_18198760 - S11_11799702	1.0	2.7	2.0	0.5	0.3	0.60	partially dominant
100-seed weight									
qSW-cim-2.1	37.7	S2_28067626 - S2_2334639	1.8	3.5	26.7	0.5	-0.5	-1.05	recessive
qSW-cim-2.2	90.7	S2_11771536 - S2_10960200	0.1	2.7	10.1	0.2	-0.5	-2.15	recessive
qSW-cim-2.3	118.2	S2_6037523 - S2_5078598	2.4	2.8	14.3	0.3	-0.6	-1.90	recessive
qSW-icim-3.1	60.0	S3_7169453 - S3_6422339	2.8	3.7	9.4	-0.4	0.2	0.41	partially dominant
qSW-cim-3.1	60.8	S3_6422339 - S3_16299670	0.1	4.8	16.9	-0.6	0.1	0.21	partially dominant

QTL name	Position	Flanking markers	QTL interval	LOD	PVE (%)	*a*	*d*	*d*/|*a*|	Gene action
qSW-icim-8.1	14.0	S8_18946297 - S8_11986619	2.0	4.4	13.1	-0.2	0.7	3.99	over dominant
qSW-icim-10.1	153.0	S10_15140940 - S10_632618	5.8	2.5	4.9	0.5	-0.5	-1.05	recessive
qSW-cim-11.1	12.3	S11_27825757 - S11_9769716	1.7	4.3	13.0	0.2	-0.6	-2.37	recessive
qSW-icim-11.1	104.0	S11_14467653 - S11_11799692	1.2	3.1	12.3	0.0	-0.7	-67.75	recessive
qSW-cim-11.2	121.7	S11_10013768 - S11_47645855	0.1	2.6	8.7	0.5	0.0	0.05	additive
Seed yield									
qSY-icim-1.1	9.0	S1_1145802 - S1_14036679	0.8	3.7	10.3	3.9	-18.8	-4.76	recessive
qSY-icim-2.1	93.0	S2_24889905 - S2_3670647	1.2	3.0	7.8	-26.7	-29.0	-1.09	recessive
qSY-cim-4.1	31.3	S4_11052197 - S4_10704406	0.7	3.3	11.3	-14.6	2.3	0.16	additive
qSY-cim-5.1	20.6	S5_2134830 - S5_2134832	6.1	3.9	39.0	55.1	-41.4	-0.75	partially dominant
qSY-icim-7.1	17.0	S7_8660317 - S7_1644160	13.8	3.3	10.7	-11.4	-13.7	-1.20	recessive
qSY-icim-7.2	111.0	S7_908957 - S7_17105607	3.0	3.7	6.4	-16.9	-16.8	-0.99	recessive
qSY-icim-8.1	46.0	S8_13861942 - S8_19001717	4.8	2.6	5.8	-12.3	-7.7	-0.63	recessive
qSY-cim-10.1	155.5	S10_15140940 - S10_632618	5.8	3.0	53.0	19.1	-41.0	-2.15	recessive
qSY-cim-11.1	84.6	S11_39685164 - S11_2994853	0.7	3.9	6.7	-2.4	-22.9	-9.49	recessive
qSY-icim-11.1	85.0	S11_39685164 - S11_2994853	0.7	3.1	10.7	0.3	-23.0	-69.84	recessive
qSY-cim-11.2	197.6	S11_10379800 - S11_39387203	14.8	4.0	10.3	51.3	-51.6	-1.00	recessive
Days to first flowering									
qDFF-icim-1.1	14.0	S1_14036679 - S1_11242012	4.6	6.8	15.2	-5.3	1.7	0.32	partially dominant
qDFF-cim-1.1	14.3	S1_11242012 - S1_15951980	0.1	3.7	14.0	-3.8	3.1	0.82	dominant
qDFF-cim-1.2	29.1	S1_16743053 - S1_11236611	0.3	2.8	11.1	-2.4	4.3	1.77	over dominant
qDFF-cim-1.3	75.1	S1_1158266 - S1_12641760	9.1	2.8	2.3	1.4	5.9	4.29	over dominant
qDFF-cim-2.1	41.0	S2_2989918 - S2_16460899	0.6	4.4	4.2	-0.1	6.3	76.31	over dominant
qDFF-cim-2.2	90.7	S2_11771536 - S2_10960200	0.1	3.0	11.2	-1.7	5.2	3.04	over dominant
qDFF-cim-2.3	133.3	S2_2989899 - S2_1760098	0.1	4.2	4.5	0.6	6.5	11.68	over dominant
qDFF-cim-3.1	2.9	S3_6422190 - S3_10282824	6.7	2.8	7.8	-0.9	4.3	4.97	over dominant
qDFF-cim-3.2	143.4	S3_4923672 - S3_4949379	1.9	2.9	2.1	3.1	4.5	1.44	over dominant
qDFF-icim-6.1	72.0	S6_18172388 - S6_12492736	1.4	5.1	14.9	1.3	7.0	5.36	over dominant

QTL name	Position	Flanking markers	QTL interval	LOD	PVE (%)	*a*	*d*	*d*/|*a*|	Gene action
qDFF-cim-6.1	72.5	S6_18172388 - S6_12492736	1.4	3.2	2.5	1.1	6.6	6.25	over dominant
qDFF-icim-6.2	95.0	S6_21912913 - S6_11368997	2.0	3.0	10.9	-3.0	5.1	1.72	over dominant
qDFF-cim-6.2	100.8	S6_11368993 - S6_16630543	1.0	3.2	14.2	3.9	-2.6	-0.67	recessive
qDFF-icim-6.3	108.0	S6_18172301 - S6_6790172	1.4	3.7	8.3	3.3	5.6	1.67	over dominant
qDFF-icim-6.4	112.0	S6_6790172 - S6_9119183	4.3	2.9	9.8	3.0	3.5	1.16	over dominant
qDFF-icim-8.1	53.0	S8_1870690 - S8_14893200	11.0	3.5	12.0	-3.8	4.2	1.10	over dominant
qDFF-cim-8.1	54.7	S8_1870690 - S8_14893200	11.0	3.9	32.4	-3.4	7.8	2.27	over dominant
qDFF-cim-8.2	76.8	S8_2216092 - S8_19416095	2.1	4.5	2.1	2.0	6.4	3.18	over dominant
qDFF-cim-10.1	2.9	S10_8436572 - S10_8682299	1.6	2.6	10.9	-1.0	6.9	6.64	over dominant
qDFF-cim-10.2	85.8	S10_15140871 - S10_12011468	0.8	2.7	5.9	-0.5	4.9	9.97	over dominant
qDFF-icim-11.1	30.0	S11_6866243 - S11_22800082	0.5	3.0	11.2	-1.9	5.1	2.69	over dominant
qDFF-cim-11.1	30.3	S11_22800082 - S11_27825774	0.0	3.2	11.1	-1.6	7.8	4.86	over dominant
qDFF-cim-11.2	60.3	S11_25088688 - S11_12824405	0.0	3.1	43.8	-4.4	7.9	1.82	over dominant
qDFF-cim-11.3	112.5	S11_38856677 - S11_1583891	0.4	3.4	28.5	-3.4	8.2	2.42	over dominant
qDFF-cim-11.4	137.4	S11_22689703 - S11_19044341	0.9	3.3	5.0	0.2	5.4	25.63	over dominant
qDFF-cim-11.5	143.2	S11_24070770 - S11_13882393	1.4	3.2	6.9	0.1	7.4	107.58	over dominant
**Pop5 (ICP 5529 x ICP 11605)**									
Seed protein content									
qPROT-cim-1.1	46.6	S1_1798648 - S1_1798766	2.6	2.6	4.4	0.3	0.1	0.33	partially dominant
qPROT-cim-2.1	27.8	S2_6930418 - S2_16133939	0.3	3.0	9.3	-0.5	0.2	0.40	partially dominant
qPROT-icim-2.1	34.0	S2_10279728 - S2_32698493	0.2	6.6	16.5	-0.4	0.4	1.00	dominant
qPROT-cim-2.2	34.0	S2_10279728 - S2_32698493	0.2	5.1	17.5	-0.7	0.3	0.43	partially dominant
qPROT-cim-2.3	38.3	S2_28049603 - S2_9984747	0.1	3.1	9.0	-0.5	0.1	0.20	additive
qPROT-cim-2.4	102.9	S2_4297468 - S2_13394656	0.4	4.0	11.8	-0.5	0.4	0.80	partially dominant
qPROT-icim-2.2	121.0	S2_16519107 - S2_16348673	4.9	7.2	11.5	-0.2	-0.5	-2.50	recessive
qPROT-icim-3.1	77.0	S3_23614170 - S3_8195933	2.3	4.9	9.0	0.6	0.1	0.17	additive
qPROT-icim-6.1	85.0	S6_3630897 - S6_11140261	0.5	3.5	7.7	-0.6	0.2	0.33	partially dominant
qPROT-cim-11.1	82.2	S11_21017392 - S11_9883313	0.2	3.2	7.2	-0.5	0.6	1.20	dominant

QTL name	Position	Flanking markers	QTL interval	LOD	PVE (%)	*a*	*d*	*d*/|*a*|	Gene action
qPROT-icim-11.1	91.0	S11_38887609 - S11_29943293	0.7	3.8	7.9	-0.5	0.2	0.40	partially dominant
qPROT-cim-11.2	95.4	S11_31519207 - S11_30807675	0.5	3.0	3.3	-0.4	0.5	1.25	over-dominant
100-seed weight									
qSW-cim-1.1	16.4	S1_7127752 - S1_5944791	14.7	7.4	10.4	0.9	0.4	0.42	partially dominant
qSW-icim-1.1	20.0	S1_5944791 - S1_5173345	11.2	15.0	31.5	1.1	0.4	0.38	partially dominant
qSW-cim-6.1	26.4	S6_4443721 - S6_4396204	3.1	3.4	8.3	0.5	-0.2	-0.32	recessivie
qSW-icim-6.1	95.0	S6_14282225 - S6_14311546	0.8	2.7	4.8	-0.2	0.5	2.21	over dominant
qSW-icim-8.1	52.0	S8_1664745 - S8_8700222	7.3	2.7	6.3	0.4	-0.5	-1.21	recessivie
qSW-icim-11.1	24.0	S11_6210775 - S11_39507811	2.7	3.3	6.0	0.1	0.6	5.93	over dominant
Seed yield									
qSY-icim-1.1	29.0	S1_5944791 - S1_5173345	11.2	3.2	6.6	0.7	19.8	26.57	over dominant
qSY-icim-1.2	74.0	S1_3905217 - S1_17462230	7.3	3.8	14.8	-13.6	-26.7	-1.97	recessive
qSY-icim-2.1	46.0	S2_28723848 - S2_11947232	1.7	4.2	8.9	-1.1	23.3	22.04	over dominant
qSY-cim-2.1	126.3	S2_8504401 - S2_4442599	0.4	2.9	8.2	-7.0	19.0	2.71	over dominant
qSY-icim-6.1	101.0	S6_14311667 - S6_14388845	1.1	4.1	8.8	15.9	-0.9	-0.06	recessive
Growth habit									
qGH-cim-2.1	54.4	S2_206675 - s2_1204754	0.5	2.8	20.7	0.2	-0.2	-1.04	recessive
qGH-icim-2.1	64.0	S2_9105026 - S2_22664490	0.2	2.7	3.9	-0.1	-0.2	-1.27	recessive
qGH-cim-3.1	5.0	S3_21310513 - S3_28538775	7.5	2.9	9.3	0.1	-0.2	-2.02	recessive
qGH-cim-3.2	14.5	S3_28538775 -S3_21244595	10.4	4.9	19.6	0.1	-0.3	-3.45	recessive
qGH-icim-3.1	20.0	S3_24127385 - S3_21274904	1.4	4.4	12.0	0.1	-0.2	-1.62	recessive
qGH-cim-3.3	23.7	S3_20631155 - S3_22234078	2.4	15.2	47.0	0.3	-0.3	-0.94	recessive
qGH-cim-3.4	34.3	s3_20698771 - S3_18430894	2.3	22.1	5.0	-0.3	-0.3	-0.98	recessive
qGH-icim-3.2	35.0	s3_20698771 - S3_18430894	2.3	29.1	61.6	-0.4	-0.3	-0.80	recessive
qGH-cim-3.5	46.6	S3_18154848 - S3_17193829	6.0	11.3	42.1	0.3	-0.2	-0.72	recessive
qGH-icim-4.1	27.0	s4_496463 - S4_487510	2.1	2.6	13.1	0.3	-0.2	-0.70	recessive
qGH-cim-11.1	24.9	S11_6210775 - S11_39507811	2.7	4.6	27.2	0.2	-0.3	-1.98	recessive
qGH-cim-11.2	54.6	S11_4453854 - S11_4725362	0.8	3.0	3.4	0.1	0.0	0.35	partially dominant

QTL name	Position	Flanking markers	QTL interval	LOD	PVE (%)	*a*	*d*	*d*/|*a*|	Gene action
qGH-icim-11.1	64.0	S11_8456082 - S11_44938548	0.6	4.1	14.0	0.2	-0.4	-1.73	recessive
Days to first flowering									
qDFF-icim-3.1	24.0	S3_22234078 - S3_16681929	5.7	6.6	12.6	-4.7	2.2	0.48	partially dominant
qDFF-cim-3.1	24.7	S3_22234078 - S3_16681929	5.7	7.8	39.6	-6.0	4.7	0.79	partially dominant
qDFF-cim-3.2	34.3	s3_20698771 - S3_18430894	2.3	7.8	4.6	5.2	4.0	0.76	partially dominant
qDFF-cim-3.3	41.6	S3_18430894 - S3_18154848	7.0	5.0	47.6	-5.6	5.5	0.98	dominant
qDFF-cim-3.4	46.6	S3_18154848 - S3_17193829	6.0	4.0	16.2	-4.0	2.7	0.66	partially dominant
qDFF-icim-3.2	117.0	S3_14754306 - S3_8040710	3.3	3.4	7.9	-2.6	3.6	1.35	over dominant
qDFF-icim-7.1	24.0	S7_13036645 - S7_12034341	1.7	2.9	6.1	0.3	-4.7	-15.95	recessive

Supplementary Table S3. Epistatic QTLs for seed protein content and agronomic traits identified using inclusive composite interval mapping (ICIM) in five pigeonpea F_2 populations

Trait	Chr1	Pos1	LM1	RM1	Chr2	Pos2	LM2	RM2	LOD	PVE (%)	[aa]	[ad]	[da]	[dd]
Pop1 (ICP 11605 × ICP 14209)														
DFF	1	65	S1_1798766	S1_1906557	11	30	S11_47645855	S11_36456560	5.2	16.6	7.2	-4.1	9.8	6.3
GH	1	35	S1_2693194	S1_4757043	2	80	S2_24059431	S2_24764841	5.0	27.2	-0.2	-0.2	0.4	-0.3
GH	1	55	S1_1575466	S1_1798766	1	60	S1_1798766	S1_1906557	8.4	37.3	-0.2	-0.1	0.6	-0.7
GH	1	70	S1_1798766	S1_1906557	3	65	S3_17441541	S3_21244595	14.0	70.2	0.0	-0.4	0.4	-0.9
GH	1	25	S1_5093650	S1_2693194	10	5	S10_4461663	S10_8216833	5.1	31.7	-0.1	0.3	-0.4	-0.4
SPC	1	30	S1_2693194	S1_4757043	2	75	S2_7491873	S2_36672875	5.0	18.0	-0.4	0.4	-0.9	1.5
SPC	1	45	S1_4757043	S1_1575466	7	25	S7_3522458	S7_12010754	5.1	15.5	0.1	-0.7	-0.5	1.5
SPC	1	45	S1_4757043	S1_1575466	8	100	S8_13310192	S8_4675310	6.6	23.8	0.5	-1.9	-0.2	0.9
SW	1	40	S1_4757043	S1_1575466	2	85	S2_36121093	S2_36167974	5.3	14.7	0.1	-0.8	-0.1	1.2
SW	1	40	S1_4757043	S1_1575466	6	95	S6_21393668	S6_3159471	5.5	18.2	-0.5	-1.1	0.8	0.7
SW	1	0	S1_7045823	S1_4374250	7	85	S7_6037045	S7_6897487	5.5	17.2	-0.3	-1.1	0.3	-0.1
SY	1	45	S1_4757043	S1_1575466	3	20	S3_12603960	S3_23502392	6.1	22.7	0.8	-4.4	-52.4	47.1
SY	1	60	S1_1798766	S1_1906557	10	75	S10_259572	S10_371125	6.5	24.7	17.5	-33.0	-25.1	21.3
SY	1	20	S1_4757283	S1_5093650	8	125	S8_9535782	S8_9669552	5.7	19.1	12.7	16.1	40.6	7.4
GH	2	20	S2_16997696	S2_10643268	4	0	S4_2222488	S4_2168590	5.0	9.4	0.0	0.2	0.0	-0.3
GH	2	65	S2_22546301	S2_7491873	2	80	S2_24059431	S2_24764841	5.5	34.5	-0.4	0.0	-0.4	0.1
GH	2	80	S2_24059431	S2_24764841	7	15	S7_12804834	S7_18463975	5.3	21.9	0.0	0.4	0.1	-0.5
GH	2	80	S2_24059431	S2_24764841	8	50	S8_5791445	S8_912171	9.0	36.9	0.1	0.7	0.1	-0.2
GH	2	80	S2_24059431	S2_24764841	10	90	S10_371125	S10_190277	9.8	33.6	0.2	0.6	0.3	-0.4
GH	2	80	S2_24059431	S2_24764841	11	210	S11_25877230	S11_29782823	9.3	30.1	-0.4	0.0	0.1	0.6
GH	2	90	S2_36167974	S2_25553275	3	65	S3_17441541	S3_21244595	10.0	66.7	0.0	-0.3	0.5	-0.6
GH	2	90	S2_36167974	S2_25553275	6	10	S6_6060973	S6_12750333	5.7	33.7	-0.2	-0.5	0.3	-0.2
SW	2	125	S2_28755005	S2_28751418	6	10	S6_6060973	S6_12750333	5.6	18.8	-0.6	-0.3	-0.2	-1.0
SY	2	10	S2_16348673	S2_16348917	5	5	S5_2580750	S5_4692912	5.2	15.8	-17.1	3.4	-10.7	37.0
SY	2	15	S2_16348917	S2_16997696	8	35	S8_2049156	S8_5791461	6.0	23.3	-16.6	32.6	-22.8	14.4

Trait	Chr1	Pos1	LM1	RM1	Chr2	Pos2	LM2	RM2	LOD	PVE (%)	[aa]	[ad]	[da]	[dd]
SY	2	20	S2_16997696	S2_10643268	6	55	S6_11455119	S6_11140261	5.2	18.0	20.3	30.4	28.7	-0.7
SY	2	55	S2_29055808	S2_15893512	10	85	S10_371125	S10_190277	6.2	23.0	-0.8	17.5	26.6	-43.8
SY	2	145	S2_29614491	S2_36085710	11	225	S11_25050893	S11_30337861	5.6	24.7	28.2	37.6	27.2	-11.2
DFF	3	65	S3_17441541	S3_21244595	8	145	S8_19166126	S8_10333523	5.3	33.4	2.6	10.6	3.3	-12.7
DFF	3	160	S3_20357542	S3_925667	6	45	S6_14282207	S6_14236163	5.0	10.4	4.3	7.9	0.6	-0.1
GH	3	65	S3_17441541	S3_21244595	3	175	S3_3913971	S3_16727150	18.4	75.8	0.0	0.4	0.4	-0.9
GH	3	65	S3_17441541	S3_21244595	5	55	S5_1264439	S5_624899	14.3	76.0	0.0	0.5	0.3	-0.7
GH	3	65	S3_17441541	S3_21244595	6	90	S6_21393668	S6_3159471	7.8	56.2	0.1	0.2	-0.2	-0.3
GH	3	65	S3_17441541	S3_21244595	7	5	S7_15796777	S7_19089666	17.7	79.5	0.2	-0.3	0.5	0.2
GH	3	65	S3_17441541	S3_21244595	8	25	S8_11972776	S8_2049156	11.9	76.4	-0.2	0.1	0.6	-0.7
GH	3	65	S3_17441541	S3_21244595	9	10	S9_9689272	S9_10221364	6.6	66.7	-0.2	0.2	0.4	-0.6
GH	3	65	S3_17441541	S3_21244595	10	5	S10_4461663	S10_8216833	14.0	79.6	0.0	0.3	-0.4	-0.9
GH	3	65	S3_17441541	S3_21244595	11	180	S11_23458079	S11_20186519	9.3	57.2	-0.1	0.4	0.0	-0.4
SPC	3	115	S3_11310314	S3_25423101	11	155	S11_12420322	S11_38211354	5.2	31.2	-1.5	-1.2	-0.3	2.1
SW	3	45	S3_21275059	S3_28586858	4	25	S4_1521545	S4_1311883	5.0	16.5	-0.6	-0.2	-0.5	0.0
SW	3	180	S3_3913971	S3_16727150	8	145	S8_19166126	S8_10333523	5.3	16.9	-0.3	-0.1	-0.9	0.9
SY	3	35	S3_16632580	S3_22917964	3	115	S3_11310314	S3_25423101	5.4	23.9	-19.0	-19.8	19.7	20.7
SY	3	20	S3_12603960	S3_23502392	5	35	S5_312017	S5_1264439	5.1	27.2	9.4	35.3	-9.5	-43.0
SY	3	35	S3_16632580	S3_22917964	11	150	S11_20646142	S11_20816375	7.8	35.2	-15.0	-17.6	4.7	54.2
SY	3	170	S3_16727124	S3_8433349	6	60	S6_21760159	S6_11433399	5.2	18.1	-10.8	-33.9	20.2	13.9
SY	3	125	S3_23737567	S3_8040710	7	60	S7_722957	S7_14067075	6.1	27.6	18.9	3.5	34.4	49.0
SY	3	140	S3_25398665	S3_5325095	8	25	S8_11972776	S8_2049156	5.6	37.0	-13.5	-10.5	45.3	-3.4
SY	3	0	S3_26745908	S3_23529670	10	15	S10_8216833	S10_14964979	6.5	15.9	-30.6	35.5	-21.5	17.0
SPC	4	55	S4_4734626	S4_9854357	6	95	S6_21393668	S6_3159471	5.1	17.3	0.0	1.0	0.8	1.0
GH	5	20	S5_4692888	S5_312017	5	50	S5_1264439	S5_624899	7.8	37.9	0.0	-0.3	0.3	-0.8
GH	5	55	S5_1264439	S5_624899	8	170	S8_17346247	S8_15097397	5.7	22.9	0.1	0.1	0.3	-0.4
GH	5	55	S5_1264439	S5_624899	10	90	S10_371125	S10_190277	5.0	23.0	0.1	0.3	-0.4	-0.3
GH	5	45	S5_312017	S5_1264439	9	5	S9_1280107	S9_9689272	5.8	19.1	0.2	-0.3	0.2	-0.3

Trait	Chr1	Pos1	LM1	RM1	Chr2	Pos2	LM2	RM2	LOD	PVE (%)	[aa]	[ad]	[da]	[dd]
GH	5	40	S5_312017	S5_1264439	11	50	S11_33138038	S11_39568168	5.8	20.6	0.2	-0.2	-0.5	0.0
SPC	5	20	S5_4692888	S5_312017	6	35	S6_14335733	S6_14282201	5.5	19.0	0.9	0.6	-0.3	1.4
SW	5	60	S5_1264439	S5_624899	10	75	S10_259572	S10_371125	5.1	14.6	0.2	-1.3	-0.4	0.1
SY	5	0	S5_2580221	S5_2580750	8	95	S8_6388803	S8_9452602	5.3	20.7	41.5	4.1	-0.6	22.0
SY	5	0	S5_2580221	S5_2580750	10	15	S10_8216833	S10_14964979	5.5	12.9	-21.3	23.5	-34.5	55.2
GH	6	45	S6_14282207	S6_14236163	6	80	S6_14551548	S6_22264720	5.2	12.5	0.0	0.2	0.3	-0.2
GH	6	85	S6_22264720	S6_21393668	11	105	S11_6013633	S11_27172559	5.9	18.6	-0.2	0.3	-0.3	-0.4
GH	6	10	S6_6060973	S6_12750333	9	5	S9_1280107	S9_9689272	5.1	19.3	0.1	-0.2	0.5	-0.3
SPC	6	15	S6_14310524	S6_14313152	8	185	S8_14218715	S8_15026624	5.2	16.7	0.3	-0.6	1.0	0.5
SPC	6	60	S6_21760159	S6_11433399	6	85	S6_22264720	S6_21393668	5.4	25.7	-1.0	-2.9	0.5	0.2
SW	6	65	S6_6237148	S6_16682610	8	145	S8_19166126	S8_10333523	5.1	25.1	-0.2	-0.9	-1.1	0.4
SY	6	60	S6_21760159	S6_11433399	8	35	S8_2049156	S8_5791461	6.4	23.9	-9.9	14.4	-28.0	36.1
SY	6	60	S6_21760159	S6_11433399	11	80	S11_9768472	S11_24859358	5.3	17.8	-8.3	15.5	-28.9	43.9
SY	6	85	S6_22264720	S6_21393668	10	5	S10_4461663	S10_8216833	5.6	22.3	-17.3	23.8	-29.3	13.0
DFF	7	20	S7_3522458	S7_12010754	9	60	S9_8998000	S9_5312289	5.4	18.2	-4.8	-0.6	-8.4	-13.3
DFF	7	20	S7_3522458	S7_12010754	11	70	S11_14777000	S11_30776997	5.5	17.6	2.9	4.9	9.7	-5.4
GH	7	65	S7_14067075	S7_14683829	8	60	S8_1666164	S8_1664745	5.5	19.2	0.1	0.0	-0.3	-0.1
GH	7	5	S7_15796777	S7_19089666	7	25	S7_3522458	S7_12010754	5.3	32.8	0.0	0.5	-0.1	-0.3
GH	7	5	S7_15796777	S7_19089666	9	15	S9_10221364	S9_1358645	6.6	26.3	0.1	-0.5	0.1	-0.2
GH	7	20	S7_3522458	S7_12010754	10	5	S10_4461663	S10_8216833	8.9	35.3	0.1	-0.3	-0.5	-0.3
SPC	7	30	S7_18419460	S7_15005750	8	85	S8_10556549	S8_19075759	5.2	12.8	0.1	-0.9	-1.3	-1.0
SPC	7	75	S7_6012566	S7_9034247	11	90	S11_23469021	S11_46615058	5.6	22.3	0.8	-0.2	0.2	1.4
SY	7	55	S7_18464007	S7_12836334	8	25	S8_11972776	S8_2049156	5.8	32.7	17.4	-45.4	-6.5	-16.6
SY	7	60	S7_722957	S7_14067075	11	150	S11_20646142	S11_20816375	5.4	26.1	12.2	39.4	12.0	21.8
GH	8	30	S8_11972776	S8_2049156	10	20	S10_14964979	S10_7161105	7.8	29.3	0.2	0.3	0.6	-0.3
GH	8	100	S8_13310192	S8_4675310	11	210	S11_25877230	S11_29782823	6.4	23.9	0.4	0.0	0.1	0.4
GH	8	135	S8_4533965	S8_14798091	9	25	S9_9670150	S9_9689983	5.5	17.4	-0.3	-0.2	0.1	0.5
GH	8	115	S8_6353157	S8_1284456	8	120	S8_13150686	S8_9535782	9.6	32.5	-0.4	0.2	0.1	0.4

Trait	Chr1	Pos1	LM1	RM1	Chr2	Pos2	LM2	RM2	LOD	PVE (%)	[aa]	[ad]	[da]	[dd]
SPC	8	30	S8_11972776	S8_2049156	11	195	S11_8456046	S11_39507811	5.5	24.4	-0.1	-1.1	-0.3	-1.7
SY	8	25	S8_11972776	S8_2049156	8	95	S8_6388803	S8_9452602	6.4	31.0	-16.6	-44.0	1.7	56.8
SY	8	150	S8_10333523	S8_4867754	11	150	S11_20646142	S11_20816375	6.8	27.8	-24.4	-29.2	13.6	23.0
SY	8	35	S8_2049156	S8_5791461	9	40	S9_10229309	S9_7755937	6.9	38.7	-30.0	-3.9	-13.5	72.0
SY	8	40	S8_2049156	S8_5791461	10	75	S10_259572	S10_371125	7.8	38.5	23.9	-26.0	-8.3	53.2
GH	9	5	S9_1280107	S9_9689272	10	20	S10_14964979	S10_7161105	7.5	31.6	-0.3	-0.3	0.7	-0.5
GH	10	5	S10_4461663	S10_8216833	10	20	S10_14964979	S10_7161105	10.7	41.6	0.0	-0.4	0.5	-0.8
GH	10	5	S10_4461663	S10_8216833	11	145	S11_32081128	S11_5166783	6.2	35.7	-0.2	0.5	-0.1	-0.2
SY	10	75	S10_259572	S10_371125	11	160	S11_38929174	S11_23233620	8.2	28.0	-28.8	-12.4	34.0	34.8
SY	10	15	S10_8216833	S10_14964979	10	50	S10_4202839	S10_1984771	6.2	16.8	28.2	-27.8	-40.0	16.0
GH	11	15	S11_5868042	S11_40289708	11	85	S11_9768477	S11_27172500	6.1	17.7	0.0	0.1	-0.5	-0.5
SY	11	95	S11_11359055	S11_7007425	11	150	S11_20646142	S11_20816375	5.3	36.4	-3.3	-19.5	23.7	65.8
Pop2 (ICP 8863 × ICP 11605)														
DFF	1	35	S1_12899653	S1_11050274	7	55	S7_12804834	S7_6897487	5.1	20.7	4.9	-11.7	-0.5	9.8
DFF	1	170	S1_13431148	S1_14032441	10	50	S10_21497752	S10_18083837	6.8	20.4	-1.9	1.5	12.1	-19.8
DFF	1	85	S1_14083660	S1_3905212	2	140	S2_13394806	S2_35442671	5.3	16.9	-4.9	-8.2	3.7	13.0
DFF	1	80	S1_652229	S1_14083660	6	55	S6_15449552	S6_18295388	5.4	21.2	-2.9	-8.4	7.5	10.2
DFF	1	80	S1_652229	S1_14083660	9	50	S9_7755937	S9_7756050	5.7	21.9	6.6	-7.5	-6.1	2.7
DFF	1	80	S1_652229	S1_14083660	8	5	S8_4714912	S8_11776420	6.2	26.2	7.0	-4.0	-9.7	-2.2
DFF	1	120	S1_9842833	S1_1158656	3	110	S3_8040710	S3_9200641	5.6	24.3	6.6	-8.6	-17.1	-2.5
GH	1	140	S1_10689415	S1_556023	6	25	S6_6237148	S6_14282225	6.5	13.4	0.3	-0.1	0.0	0.3
GH	1	135	S1_10689415	S1_556023	3	25	S3_21244595	S3_18933167	8.8	35.2	-0.1	0.6	0.3	-0.6
GH	1	155	S1_12591177	S1_13431089	10	135	S10_17099642	S10_6765628	11.1	18.9	0.1	0.1	-0.6	-0.4
GH	1	155	S1_12591177	S1_13431089	11	90	S11_39309852	S11_28894118	9.3	21.4	0.0	0.2	-0.6	-0.5
GH	1	5	S1_16910575	S1_16294075	2	15	S2_20782748	S2_36429055	9.0	16.9	0.3	-0.2	-0.3	0.1
GH	1	10	S1_4759267	S1_15329865	7	5	S7_18419460	S7_6037045	7.2	16.7	0.2	-0.3	-0.2	0.1
GH	1	10	S1_4759267	S1_15329865	8	60	S8_11838449	S8_11838448	5.9	19.2	0.2	-0.3	-0.1	0.5
GH	1	10	S1_4759267	S1_15329865	1	80	S1_652229	S1_14083660	6.4	20.9	0.2	-0.3	-0.1	0.5

Trait	Chr1	Pos1	LM1	RM1	Chr2	Pos2	LM2	RM2	LOD	PVE (%)	[aa]	[ad]	[da]	[dd]
GH	1	80	S1_652229	S1_14083660	4	0	S4_2849401	S4_2014532	6.5	15.6	0.1	-0.2	-0.4	0.1
GH	1	80	S1_652229	S1_14083660	9	75	S9_10177047	S9_10172092	6.8	20.9	-0.4	0.2	-0.2	0.6
GH	1	80	S1_652229	S1_14083660	5	25	S5_4692912	S5_4199522	12.3	37.0	-0.2	0.3	-0.5	0.3
SPC	1	55	S1_5552696	S1_3905151	10	155	S10_19001995	S10_7783201	5.5	60.5	-1.1	1.1	1.1	1.9
SW	1	75	S1_10466763	S1_3905220	2	120	S2_21890021	S2_7683449	16.2	34.0	0.2	0.2	5.1	-5.5
SW	1	40	S1_11050274	S1_748256	3	0	S3_22234078	S3_19578263	13.8	31.9	0.1	5.2	-0.4	-5.0
SW	1	40	S1_11050274	S1_748256	6	70	S6_18322873	S6_18322737	11.4	32.0	-0.2	5.2	0.4	-4.7
SW	1	40	S1_11050274	S1_748256	4	15	S4_1521545	S4_1710877	9.8	32.9	2.6	-2.2	-2.3	2.3
SW	1	95	S1_14067713	S1_9603077	7	10	S7_6037077	S7_18542722	13.9	32.9	-2.8	2.2	-2.8	2.6
SW	1	95	S1_14067713	S1_9603077	8	10	S8_4714912	S8_11776420	10.6	39.0	-2.1	3.3	-2.2	2.9
SW	1	100	S1_14069006	S1_9863131	5	5	S5_5120324	S5_4692888	14.1	29.8	-2.5	-2.6	2.5	2.6
SW	1	85	S1_14083660	S1_3905212	11	110	S11_27472029	S11_20186519	15.1	36.1	2.6	2.9	3.1	2.3
SW	1	5	S1_16910575	S1_16294075	1	10	S1_4759267	S1_15329865	15.6	40.7	-2.1	2.8	-2.1	2.9
SW	1	10	S1_4759267	S1_15329865	9	25	S9_1280000	S9_7212583	16.7	39.8	2.6	3.0	2.1	2.0
SW	1	10	S1_4759267	S1_15329865	10	40	S10_21497771	S10_13762076	16.6	41.8	2.5	3.3	2.3	2.7
SY	1	10	S1_4759267	S1_15329865	10	200	S10_7783201	S10_5097784	6.2	35.6	14.3	-0.2	31.8	-13.1
SY	1	170	S1_13431148	S1_14032441	7	40	S7_10467966	S7_12804834	5.4	13.0	9.5	13.4	1.2	22.1
SY	1	95	S1_14067713	S1_9603077	1	140	S1_10689415	S1_556023	6.2	19.1	13.0	10.1	18.4	17.7
SY	1	60	S1_3905151	S1_5414823	3	20	S3_28933239	S3_21244595	5.1	11.5	2.8	-0.7	11.2	-29.7
SY	1	10	S1_4759267	S1_15329865	11	65	S11_30337861	S11_12765240	6.6	30.7	-12.8	14.3	-28.9	-21.7
DFF	2	170	S2_13394818	S2_26969919	11	145	S11_20304380	S11_38654219	5.3	14.8	-8.3	4.1	-4.0	4.5
DFF	2	170	S2_13394818	S2_26969919	9	50	S9_7755937	S9_7756050	5.5	16.9	-5.6	1.6	-6.7	11.6
DFF	2	15	S2_20782748	S2_36429055	10	55	S10_18083837	S10_17801329	6.0	26.6	-2.3	-4.1	13.7	-17.6
DFF	2	5	S2_20782748	S2_36429055	3	35	S3_18933167	S3_18929445	5.0	44.3	1.5	15.8	2.9	-3.6
DFF	2	95	S2_35584049	S2_22473218	2	185	S2_25523315	S2_28049878	5.7	34.9	-5.7	27.4	5.3	-10.4
GH	2	130	S2_10607410	S2_32197339	11	90	S11_39309852	S11_28894118	8.5	17.4	0.3	0.4	0.4	0.1
GH	2	75	S2_16111815	S2_21929010	8	65	S8_11838449	S8_11838448	6.4	13.7	0.3	-0.2	-0.1	0.4
GH	2	60	S2_19392675	S2_13388933	7	90	S7_14683829	S7_9034247	6.6	13.8	0.1	0.4	0.0	-0.6

Trait	Chr1	Pos1	LM1	RM1	Chr2	Pos2	LM2	RM2	LOD	PVE (%)	[aa]	[ad]	[da]	[dd]
GH	2	10	S2_20782748	S2_36429055	9	70	S9_10229309	S9_10177047	5.9	19.6	0.1	-0.3	0.0	0.7
GH	2	15	S2_20782748	S2_36429055	2	35	S2_36429066	S2_19392689	7.0	22.4	-0.2	0.5	-0.2	-0.4
GH	2	10	S2_20782748	S2_36429055	5	30	S5_4692912	S5_4199522	6.3	24.2	0.1	-0.4	-0.4	0.0
GH	2	115	S2_21890021	S2_7683449	10	135	S10_17099642	S10_6765628	11.4	20.7	0.0	0.1	-0.6	-0.6
GH	2	145	S2_32807766	S2_7683443	6	40	S6_14282226	S6_14335833	5.4	13.0	0.1	-0.4	0.0	-0.5
GH	2	90	S2_35584049	S2_22473218	4	0	S4_2849401	S4_2014532	6.5	10.4	0.3	-0.2	-0.3	0.2
GH	2	35	S2_36429066	S2_19392689	3	45	S3_18929445	S3_18929378	15.8	59.8	-0.3	0.1	0.6	-0.5
SPC	2	80	S2_22473129	S2_18386711	10	155	S10_19001995	S10_7783201	6.3	67.0	0.0	-2.9	1.6	2.5
SW	2	80	S2_22473129	S2_18386711	3	0	S3_22234078	S3_19578263	14.6	31.9	-0.2	5.2	0.2	-4.5
SW	2	80	S2_22473129	S2_18386711	4	25	S4_1710877	S4_839628	15.3	33.7	2.7	-2.1	-2.1	2.1
SW	2	80	S2_22473129	S2_18386711	2	115	S2_21890021	S2_7683449	15.5	34.1	-2.5	-2.5	3.0	2.2
SW	2	55	S2_19392675	S2_13388933	9	75	S9_10177047	S9_10172092	13.6	32.2	0.3	4.8	0.0	-5.7
SW	2	60	S2_19392675	S2_13388933	5	10	S5_4692912	S5_4199522	17.1	36.5	-2.7	-2.3	3.3	1.3
SW	2	115	S2_21890021	S2_7683449	6	65	S6_18295384	S6_18322776	13.9	35.8	-2.4	2.9	-2.4	3.2
SW	2	125	S2_34204720	S2_7683447	11	15	S11_27612418	S11_32832892	16.0	31.9	-0.1	5.3	-0.2	-4.3
SW	2	125	S2_34204720	S2_7683447	7	5	S7_18419460	S7_6037045	15.8	32.7	2.7	-2.1	-2.8	2.7
SW	2	95	S2_35584049	S2_22473218	8	30	S8_11776408	S8_11838449	14.4	38.9	2.9	-2.4	-3.0	1.3
SW	2	40	S2_36429066	S2_19392689	10	130	S10_18083991	S10_17099642	13.9	34.8	-2.6	-2.7	3.0	1.5
SY	2	130	S2_10607410	S2_32197339	2	150	S2_36008860	S2_32734003	5.0	17.0	3.6	7.4	-16.0	-43.1
SY	2	0	S2_20782748	S2_36429055	6	5	S6_2496170	S6_6237148	5.3	14.0	-6.5	2.4	-12.5	23.7
SY	2	10	S2_20782748	S2_36429055	10	110	S10_8438690	S10_13626722	6.3	26.0	-23.7	0.8	-4.6	9.3
SY	2	15	S2_20782748	S2_36429055	11	110	S11_27472029	S11_20186519	5.5	27.3	-8.7	-0.5	-39.8	2.2
SY	2	120	S2_21890021	S2_7683449	7	0	S7_18419460	S7_6037045	5.5	10.6	-21.2	0.2	20.9	7.8
SY	2	120	S2_21890021	S2_7683449	8	25	S8_11776420	S8_11776408	5.2	11.4	5.6	30.3	-18.8	-11.8
SY	2	160	S2_36164833	S2_16111897	3	5	S3_19578263	S3_21274904	7.3	36.4	-7.6	-39.4	8.4	-12.5
DFF	3	135	S3_8772530	S3_1358533	11	150	S11_22893601	S11_10881649	5.8	22.9	-7.8	-11.9	4.3	1.7
DFF	3	80	S3_14758073	S3_11310314	8	5	S8_4714912	S8_11776420	6.2	25.9	-7.2	0.8	-8.4	5.3
DFF	3	75	S3_14758073	S3_11310314	6	25	S6_6237148	S6_14282225	5.9	27.4	6.1	6.7	9.7	9.2

Trait	Chr1	Pos1	LM1	RM1	Chr2	Pos2	LM2	RM2	LOD	PVE (%)	[aa]	[ad]	[da]	[dd]
DFF	3	5	S3_19578263	S3_21274904	10	55	S10_18083837	S10_17801329	6.8	23.2	0.4	0.4	17.9	-12.6
GH	3	45	S3_18929445	S3_18929378	6	25	S6_6237148	S6_14282225	5.3	39.1	0.0	-0.4	0.0	0.2
GH	3	45	S3_18929445	S3_18929378	11	55	S11_33954110	S11_42065843	12.9	55.4	0.3	0.6	0.3	-0.1
GH	3	45	S3_18929445	S3_18929378	5	25	S5_4692912	S5_4199522	15.4	63.7	0.0	0.4	-0.5	0.1
GH	3	30	S3_21244595	S3_18933167	8	60	S8_11838449	S8_11838448	7.5	38.2	0.0	0.3	0.4	-0.7
GH	3	25	S3_21244595	S3_18933167	10	25	S10_14148444	S10_12011468	14.1	45.2	-0.2	0.2	-0.5	-0.4
GH	3	20	S3_28933239	S3_21244595	9	35	S9_7212583	S9_7755937	7.0	47.3	0.2	0.1	-0.5	-0.5
GH	3	20	S3_28933239	S3_21244595	3	45	S3_18929445	S3_18929378	1132.5	99.4	0.0	0.0	-1.0	0.0
SPC	3	130	S3_8772530	S3_1358533	10	155	S10_19001995	S10_7783201	5.5	67.4	-1.1	-0.1	1.1	-0.6
SW	3	90	S3_11310314	S3_10301855	9	20	S9_1280000	S9_7212583	13.1	31.4	2.6	2.5	2.5	2.6
SW	3	90	S3_11310314	S3_10301855	11	110	S11_27472029	S11_20186519	14.6	32.4	2.7	2.5	3.0	3.0
SW	3	70	S3_14758073	S3_11310314	4	15	S4_1521545	S4_1710877	11.1	34.6	2.4	-1.9	-2.3	2.6
SW	3	75	S3_14758073	S3_11310314	7	50	S7_12804834	S7_6897487	10.1	35.0	0.1	5.3	0.1	-4.7
SW	3	80	S3_14758073	S3_11310314	10	25	S10_14148444	S10_12011468	15.2	35.7	2.5	3.0	2.0	2.8
SW	3	75	S3_14758073	S3_11310314	6	0	S6_2496170	S6_6237148	7.8	35.8	-2.8	2.9	-2.5	2.0
SW	3	30	S3_21244595	S3_18933167	3	45	S3_18929445	S3_18929378	14.5	31.1	-2.6	-2.7	2.8	3.2
SW	3	10	S3_21274904	S3_28538775	8	40	S8_11776408	S8_11838449	14.4	34.6	-0.1	-0.5	5.0	-5.5
SW	3	0	S3_22234078	S3_19578263	5	35	S5_4692912	S5_4199522	14.7	33.6	0.3	0.1	-5.2	-5.1
SY	3	5	S3_19578263	S3_21274904	10	120	S10_13626722	S10_18083991	5.5	32.3	7.0	7.1	23.0	-42.2
SY	3	0	S3_22234078	S3_19578263	3	25	S3_21244595	S3_18933167	5.3	19.1	6.8	2.3	30.9	-54.8
GH	4	10	S4_2849401	S4_2014532	4	25	S4_1710877	S4_839628	5.7	10.8	-0.1	-0.6	0.4	0.0
GH	4	5	S4_2849401	S4_2014532	9	70	S9_10229309	S9_10177047	6.0	10.9	0.2	-0.2	-0.1	0.2
GH	4	0	S4_2849401	S4_2014532	11	90	S11_39309852	S11_28894118	9.1	14.5	-0.3	-0.3	0.3	0.4
GH	4	5	S4_2849401	S4_2014532	7	5	S7_18419460	S7_6037045	5.7	15.8	0.2	-0.2	-0.3	-0.1
GH	4	5	S4_2849401	S4_2014532	10	135	S10_17099642	S10_6765628	8.1	16.6	-0.3	-0.3	0.4	0.3
GH	4	5	S4_2849401	S4_2014532	6	25	S6_6237148	S6_14282225	5.7	20.1	0.2	-0.1	-0.2	0.6
GH	4	5	S4_2849401	S4_2014532	5	25	S5_4692912	S5_4199522	5.0	20.6	0.1	-0.5	-0.3	0.0
SW	4	15	S4_1521545	S4_1710877	5	35	S5_4692912	S5_4199522	11.6	33.5	-2.5	-2.9	2.3	2.2

Trait	Chr1	Pos1	LM1	RM1	Chr2	Pos2	LM2	RM2	LOD	PVE (%)	[aa]	[ad]	[da]	[dd]
SW	4	15	S4_1521545	S4_1710877	4	25	S4_1710877	S4_839628	15.4	35.3	-2.5	-3.3	2.2	2.7
SW	4	20	S4_1710877	S4_839628	10	40	S10_21497771	S10_13762076	11.7	32.6	-2.4	-2.2	2.4	3.0
SW	4	20	S4_1710877	S4_839628	9	20	S9_1280000	S9_7212583	11.7	36.0	-2.3	-1.9	2.9	3.4
SW	4	25	S4_1710877	S4_839628	11	55	S11_33954110	S11_42065843	12.0	36.0	-3.0	-2.5	2.1	1.7
GH	5	10	S5_4692912	S5_4199522	11	90	S11_39309852	S11_28894118	8.8	15.1	-0.2	-0.3	0.4	0.2
GH	5	20	S5_4692912	S5_4199522	5	35	S5_4692912	S5_4199522	8.8	27.6	0.1	0.4	-0.3	-0.7
GH	5	30	S5_4692912	S5_4199522	10	150	S10_19001995	S10_7783201	12.0	40.7	-0.2	-0.6	0.5	0.1
SPC	5	30	S5_4692912	S5_4199522	6	5	S6_2496170	S6_6237148	6.0	55.0	0.0	1.5	-0.6	3.6
SPC	5	25	S5_4692912	S5_4199522	10	155	S10_19001995	S10_7783201	6.1	64.8	-0.2	0.9	0.6	4.1
SW	5	20	S5_4692912	S5_4199522	10	115	S10_13626722	S10_18083991	15.0	32.5	-2.5	2.5	-2.8	3.2
SW	5	20	S5_4692912	S5_4199522	6	0	S6_2496170	S6_6237148	12.8	32.8	-2.5	2.4	-2.3	3.2
SW	5	25	S5_4692912	S5_4199522	7	5	S7_18419460	S7_6037045	15.3	36.9	-2.7	2.4	-2.8	3.5
SW	5	15	S5_4692912	S5_4199522	8	40	S8_11776408	S8_11838449	15.6	37.5	-2.6	2.7	-1.9	3.5
SW	5	25	S5_4692912	S5_4199522	11	50	S11_3238944	S11_3724414	16.4	38.7	-2.4	2.8	-3.2	1.6
SW	5	0	S5_5120324	S5_4692888	5	15	S5_4692912	S5_4199522	15.2	36.2	-2.0	2.9	-4.0	3.4
SW	5	5	S5_5120324	S5_4692888	9	50	S9_7755937	S9_7756050	16.4	32.9	-2.6	2.4	-2.1	3.2
DFF	6	50	S6_14335778	S6_15449552	10	95	S10_21365631	S10_16278049	5.5	17.3	6.4	4.5	7.1	14.3
DFF	6	90	S6_22082423	S6_22394558	11	215	S11_10013681	S11_32879360	5.4	27.1	-9.4	6.6	0.2	9.8
DFF	6	10	S6_6237148	S6_14282225	6	20	S6_6237148	S6_14282225	5.0	34.7	-4.3	-18.7	6.2	-8.9
GH	6	0	S6_2496170	S6_6237148	7	5	S7_18419460	S7_6037045	5.7	11.6	-0.2	0.1	-0.1	0.4
GH	6	5	S6_2496170	S6_6237148	10	135	S10_17099642	S10_6765628	7.9	18.9	0.1	0.1	-0.5	-0.5
GH	6	15	S6_6237148	S6_14282225	11	90	S11_39309852	S11_28894118	6.2	16.3	0.0	0.1	-0.6	-0.5
GH	6	30	S6_6237148	S6_14282225	6	45	S6_14336019	S6_14388845	5.3	19.1	0.0	0.7	-0.1	0.0
GH	6	25	S6_6237148	S6_14282225	9	20	S9_1280000	S9_7212583	5.4	21.0	0.2	-0.2	-0.2	0.1
SW	6	60	S6_15449552	S6_18295388	10	205	S10_7783201	S10_5097784	13.1	33.0	-2.5	2.2	-2.7	3.2
SW	6	60	S6_15449552	S6_18295388	6	65	S6_18295384	S6_18322776	13.3	33.9	-2.0	2.6	-2.4	2.7
SW	6	60	S6_15449552	S6_18295388	11	110	S11_27472029	S11_20186519	14.3	34.0	2.2	1.7	3.2	3.4
SW	6	0	S6_2496170	S6_6237148	9	20	S9_1280000	S9_7212583	13.0	31.5	-2.5	-2.5	2.0	2.6

Trait	Chr1	Pos1	LM1	RM1	Chr2	Pos2	LM2	RM2	LOD	PVE (%)	[aa]	[ad]	[da]	[dd]
SW	6	5	S6_2496170	S6_6237148	7	5	S7_18419460	S7_6037045	6.6	34.2	-2.7	2.6	-2.3	3.0
SW	6	15	S6_6237148	S6_14282225	8	35	S8_11776408	S8_11838449	9.6	35.3	-0.1	-0.2	4.6	-5.0
SY	6	15	S6_6237148	S6_14282225	10	105	S10_16278049	S10_8438690	6.3	25.4	20.8	3.2	2.9	28.4
GH	7	85	S7_14683829	S7_9034247	8	60	S8_11838449	S8_11838448	5.3	17.7	0.0	-0.1	0.4	-0.5
GH	7	5	S7_18419460	S7_6037045	11	90	S11_39309852	S11_28894118	7.3	15.9	-0.2	-0.3	0.2	0.4
GH	7	5	S7_18419460	S7_6037045	10	135	S10_17099642	S10_6765628	9.5	17.9	-0.3	-0.3	0.3	0.2
GH	7	70	S7_6897487	S7_14683829	7	80	S7_14683829	S7_9034247	5.7	18.4	-0.2	0.3	-0.3	-0.5
SW	7	50	S7_12804834	S7_6897487	8	40	S8_11776408	S8_11838449	14.1	37.2	0.4	0.0	4.9	-5.8
SW	7	5	S7_18419460	S7_6037045	10	135	S10_17099642	S10_6765628	14.6	32.6	-2.7	-2.8	2.2	2.6
SW	7	5	S7_18419460	S7_6037045	11	110	S11_27472029	S11_20186519	14.6	33.1	-2.8	-2.8	2.9	2.4
SW	7	0	S7_18419460	S7_6037045	7	10	S7_6037077	S7_18542722	13.0	34.3	-2.7	-2.1	1.2	3.2
SW	7	5	S7_18419460	S7_6037045	9	20	S9_1280000	S9_7212583	14.2	35.1	-2.5	-2.1	2.6	3.3
SY	7	5	S7_18419460	S7_6037045	10	205	S10_7783201	S10_5097784	6.5	31.5	-17.3	-32.0	32.4	-17.4
DFF	8	15	S8_4714912	S8_11776420	10	55	S10_18083837	S10_17801329	5.2	21.9	-0.8	-2.3	15.6	-17.1
DFF	8	15	S8_4714912	S8_11776420	8	20	S8_11776420	S8_11776408	5.9	28.6	-1.0	21.4	-22.9	-15.9
GH	8	65	S8_11838449	S8_11838448	11	90	S11_39309852	S11_28894118	10.2	19.3	-0.2	-0.2	0.3	0.5
GH	8	60	S8_11838449	S8_11838448	10	135	S10_17099642	S10_6765628	10.3	20.3	0.0	-0.1	-0.6	-0.5
GH	8	15	S8_4714912	S8_11776420	8	25	S8_11776420	S8_11776408	5.4	22.2	0.0	-0.2	0.3	-0.8
SW	8	25	S8_11776420	S8_11776408	8	35	S8_11776408	S8_11838449	13.9	38.0	-2.0	-3.3	2.7	0.8
SW	8	50	S8_11838449	S8_11838448	10	25	S10_14148444	S10_12011468	11.4	32.2	0.3	-0.1	-4.8	-4.6
SW	8	5	S8_4714912	S8_11776420	11	5	S11_2882386	S11_27612418	13.6	35.3	2.4	-2.8	-2.7	1.4
SW	8	10	S8_4714912	S8_11776420	9	15	S9_1280000	S9_7212583	13.7	39.7	-2.0	-2.7	2.8	2.5
DFF	9	45	S9_7212583	S9_7755937	10	100	S10_21365631	S10_16278049	5.8	18.8	-3.4	-9.1	5.7	18.0
DFF	9	75	S9_10177047	S9_10172092	11	55	S11_33954110	S11_42065843	6.6	23.9	5.9	-4.9	-15.0	4.8
GH	9	35	S9_7212583	S9_7755937	10	135	S10_17099642	S10_6765628	8.3	21.2	-0.2	-0.4	0.4	0.1
GH	9	20	S9_1280000	S9_7212583	11	90	S11_39309852	S11_28894118	9.2	20.5	-0.3	-0.1	0.3	0.7
GH	9	20	S9_1280000	S9_7212583	9	40	S9_7212583	S9_7755937	7.3	27.1	0.1	-0.2	0.3	-0.6
SPC	9	35	S9_7212583	S9_7755937	10	170	S10_19001995	S10_7783201	5.2	56.6	0.0	0.5	0.2	5.1

Trait	Chr1	Pos1	LM1	RM1	Chr2	Pos2	LM2	RM2	LOD	PVE (%)	[aa]	[ad]	[da]	[dd]
SW	9	20	S9_1280000	S9_7212583	10	165	S10_19001995	S10_7783201	13.7	41.4	-2.4	3.1	-1.8	3.9
SW	9	55	S9_7756050	S9_9689983	9	65	S9_9689983	S9_10003418	14.4	31.7	-2.8	-2.1	1.8	2.8
SW	9	55	S9_7756050	S9_9689983	11	170	S11_47251892	S11_7007425	15.3	35.6	-2.6	2.5	-2.1	3.8
SY	9	15	S9_1280000	S9_7212583	10	150	S10_19001995	S10_7783201	5.3	30.1	9.6	2.3	40.4	3.1
DFF	10	55	S10_18083837	S10_17801329	10	70	S10_17702463	S10_21497805	6.4	26.3	-9.6	-7.3	5.0	15.7
DFF	10	55	S10_18083837	S10_17801329	11	25	S11_27612418	S11_32832892	8.9	29.2	2.0	17.3	6.6	-15.6
GH	10	140	S10_19001995	S10_7783201	10	150	S10_19001995	S10_7783201	12.7	36.6	-0.1	-0.5	-0.5	-0.6
GH	10	135	S10_17099642	S10_6765628	11	150	S11_22893601	S11_10881649	10.2	18.9	0.0	0.5	0.1	0.6
SPC	10	155	S10_19001995	S10_7783201	11	5	S11_2882386	S11_27612418	7.5	66.4	0.3	1.1	-2.5	2.7
SPC	10	160	S10_19001995	S10_7783201	10	195	S10_7783201	S10_5097784	6.1	69.8	0.8	1.7	-0.1	-0.6
SW	10	40	S10_21497771	S10_13762076	11	50	S11_3238944	S11_3724414	15.4	33.7	-2.3	2.6	-2.8	3.1
SW	10	10	S10_8791174	S10_14148444	10	15	S10_8791174	S10_14148444	14.8	32.8	-2.7	-2.8	2.4	2.4
SY	10	95	S10_21365631	S10_16278049	11	65	S11_30337861	S11_12765240	6.7	24.2	-7.7	-16.2	28.8	-6.2
DFF	11	55	S11_33954110	S11_42065843	11	215	S11_10013681	S11_32879360	5.7	29.1	11.8	-6.8	-2.0	12.2
GH	11	90	S11_39309852	S11_28894118	11	105	S11_28846566	S11_25427809	9.5	18.4	-0.3	0.3	-0.2	0.5
SPC	11	20	S11_27612418	S11_32832892	11	215	S11_10013681	S11_32879360	7.1	59.8	0.4	-1.5	-0.6	3.1
SW	11	40	S11_177204	S11_250057	11	55	S11_33954110	S11_42065843	15.6	32.0	-2.6	-2.9	2.5	2.5
SY	11	165	S11_41096347	S11_44938548	11	215	S11_10013681	S11_32879360	6.2	34.0	-14.9	7.7	36.1	-13.6
Pop3 (HPL 24 × ICP 11605)														
DFF	1	120	S1_4961612	S1_679068	6	15	S6_20778715	S6_21140545	5.4	10.9	-3.0	2.5	10.2	7.9
DFF	1	105	S1_12001647	S1_14036679	7	85	S7_15005750	S7_18208587	5.5	8.8	2.7	-6.0	-1.5	-11.7
DFF	1	100	S1_12001584	S1_12001647	8	100	S8_18579673	S8_15137210	5.0	6.9	2.5	-1.2	-9.0	5.8
GH	1	20	S1_887236	S1_3399209	2	80	S2_28723848	S2_6405369	6.5	15.7	0.1	-0.1	-0.5	-0.4
GH	1	115	S1_14036679	S1_4961612	1	125	S1_679069	S1_5445088	5.4	17.5	-0.2	0.1	-0.3	0.3
GH	1	120	S1_4961612	S1_679068	3	20	S3_21244595	S3_22913898	17.1	67.1	0.0	0.1	0.4	-0.3
GH	1	125	S1_679069	S1_5445088	4	5	S4_3592410	S4_2761907	15.0	31.0	0.1	0.5	-0.2	-0.7
GH	1	50	S1_11314978	S1_9401795	6	95	S6_6094182	S6_3447497	9.3	18.8	0.4	0.2	0.0	0.6
GH	1	115	S1_14036679	S1_4961612	8	85	S8_10333523	S8_18579673	7.6	18.2	-0.3	0.3	-0.2	-0.1

Trait	Chr1	Pos1	LM1	RM1	Chr2	Pos2	LM2	RM2	LOD	PVE(%)	[aa]	[ad]	[da]	[dd]
GH	1	50	S1_11314978	S1_9401795	9	20	S9_10003418	S9_8998000	6.9	14.0	0.3	0.4	0.1	0.6
GH	1	100	S1_12001584	S1_12001647	10	10	S10_6745618	S10_18754549	6.5	14.1	0.0	0.0	0.5	-0.4
GH	1	95	S1_4066143	S1_12001584	11	50	S11_10798039	S11_27887408	5.2	17.8	0.0	-0.1	0.5	-0.3
SPC	1	20	S1_887236	S1_3399209	3	235	S3_11414215	S3_19102565	5.5	19.3	0.1	0.9	-1.4	-2.3
SW	1	20	S1_887236	S1_3399209	2	75	S2_23068242	S2_36264850	9.0	24.6	-0.9	-0.5	2.6	2.7
SW	1	25	S1_1798766	S1_1575466	1	35	S1_11361258	S1_3361403	6.5	20.1	-0.5	0.3	1.9	2.7
SW	1	35	S1_11361258	S1_3361403	3	160	S3_23347149	S3_5324878	7.6	20.0	-1.6	-1.0	-1.4	-1.5
SW	1	110	S1_12001647	S1_14036679	4	70	S4_839628	S4_1521545	6.1	16.5	1.3	1.9	-1.4	-2.0
SW	1	120	S1_4961612	S1_679068	6	100	S6_6094182	S6_3447497	6.1	21.3	-1.0	0.6	1.7	-2.4
SW	1	35	S1_11361258	S1_3361403	7	80	S7_15005750	S7_18208587	5.4	18.8	1.4	-1.1	1.1	-0.9
SW	1	35	S1_11361258	S1_3361403	8	55	S8_7083582	S8_6798921	6.7	19.0	-1.3	-1.2	-1.3	-0.8
SW	1	35	S1_11361258	S1_3361403	9	25	S9_7756050	S9_8995134	8.1	21.3	1.2	-1.2	1.3	-2.1
SW	1	95	S1_4066143	S1_12001584	10	20	S10_6745618	S10_18754549	7.4	15.1	1.7	-1.5	1.3	-1.1
SW	1	75	S1_7127752	S1_8856852	11	165	S11_48519738	S11_22689710	7.3	24.9	-0.8	1.0	2.2	-2.2
SY	1	155	S1_17365797	S1_16757180	2	225	S2_9110747	S2_20521571	5.6	23.6	20.1	8.1	0.9	15.9
SY	1	85	S1_381039	S1_9111014	6	40	S6_14282200	S6_14335733	5.8	18.7	8.4	15.8	13.5	31.5
SY	1	155	S1_17365797	S1_16757180	7	80	S7_15005750	S7_18208587	5.1	14.1	-10.6	6.8	-14.1	17.3
SY	1	95	S1_4066143	S1_12001584	8	110	S8_14218715	S8_15841326	7.8	26.0	3.7	-7.0	-2.5	38.6
SY	1	155	S1_17365797	S1_16757180	10	10	S10_6745618	S10_18754549	6.3	20.6	15.9	7.1	21.5	28.9
SY	1	155	S1_17365797	S1_16757180	11	110	S11_26654248	S11_39257707	5.7	18.9	12.5	14.1	17.2	-1.9
DFF	2	120	S2_24924004	S2_11759659	3	180	S3_18695411	S3_22966656	5.7	6.3	-4.6	1.8	-3.2	16.4
DFF	2	170	S2_6037490	S2_14842741	5	15	S5_4108250	S5_4692888	5.4	13.7	-9.0	-9.5	0.0	3.2
DFF	2	110	S2_36167974	S2_33146528	10	10	S10_6745618	S10_18754549	6.1	14.6	-0.9	4.3	-13.0	17.2
GH	2	55	S2_7373516	S2_26876108	2	60	S2_26876108	S2_36085710	6.8	17.4	-0.1	-0.4	0.1	-0.4
GH	2	185	S2_3012518	S2_36010450	3	20	S3_21244595	S3_22913898	16.2	67.1	0.2	0.3	0.2	-0.5
GH	2	155	S2_34522392	S2_6500923	4	5	S4_3592410	S4_2761907	11.8	25.8	0.0	0.1	0.0	0.9
GH	2	80	S2_28723848	S2_6405369	5	70	S5_4199522	S5_624899	5.7	16.7	0.2	0.3	0.1	0.2
GH	2	35	S2_5077845	S2_26647031	6	95	S6_6094182	S6_3447497	5.5	20.1	-0.2	-0.1	0.6	-0.3

Trait	Chr1	Pos1	LM1	RM1	Chr2	Pos2	LM2	RM2	LOD	PVE (%)	[aa]	[ad]	[da]	[dd]
GH	2	170	S2_6037490	S2_14842741	7	30	S7_1393902	S7_2664466	5.1	16.2	0.1	-0.4	0.0	-0.5
GH	2	80	S2_28723848	S2_6405369	8	50	S8_7083582	S8_6798921	7.8	19.0	-0.1	-0.3	0.1	-0.7
GH	2	225	S2_9110747	S2_20521571	9	70	S9_1280107	S9_1272426	5.5	16.5	0.2	-0.3	-0.2	0.2
GH	2	55	S2_7373516	S2_26876108	10	40	S10_9867010	S10_18754605	13.7	27.4	0.0	-0.1	0.6	-0.2
GH	2	185	S2_3012518	S2_36010450	11	120	S11_32081128	S11_16965469	7.3	21.1	-0.6	0.0	0.0	0.2
SW	2	60	S2_26876108	S2_36085710	2	115	S2_33146528	S2_31710312	9.7	22.0	1.4	1.4	-1.2	-0.1
SW	2	185	S2_3012518	S2_36010450	3	35	S3_17628375	S3_17145449	9.9	37.1	1.7	0.0	-3.2	-0.6
SW	2	75	S2_23068242	S2_36264850	4	5	S4_3592410	S4_2761907	9.5	28.4	1.7	-2.6	0.7	-3.7
SW	2	75	S2_23068242	S2_36264850	5	10	S5_4108250	S5_4692888	7.8	21.8	-0.9	3.3	-1.0	2.6
SW	2	115	S2_33146528	S2_31710312	6	55	S6_14316872	S6_12750333	7.6	20.3	1.2	-1.9	1.1	-1.5
SW	2	105	S2_14274387	S2_20017568	7	45	S7_2664466	S7_2709523	6.5	22.4	-0.5	-2.0	-1.4	-2.0
SW	2	105	S2_14274387	S2_20017568	8	35	S8_5590896	S8_7083582	8.5	25.2	-1.3	0.2	-1.1	-1.6
SW	2	105	S2_14274387	S2_20017568	9	10	S9_10221364	S9_10172092	9.5	24.2	1.3	-1.6	0.7	-0.4
SW	2	180	S2_20785495	S2_3012518	10	60	S10_4935601	S10_22177883	8.0	18.3	1.7	1.9	-1.3	0.0
SW	2	115	S2_33146528	S2_31710312	11	130	S11_27400742	S11_39685563	8.6	20.3	1.5	-1.3	1.2	-0.7
SY	2	70	S2_27324056	S2_17356325	2	175	S2_36429055	S2_29055889	6.0	22.1	0.6	3.0	-30.2	-37.7
SY	2	175	S2_36429055	S2_29055889	3	45	S3_18154848	S3_17193829	6.2	23.6	19.1	-3.1	14.0	12.5
SY	2	175	S2_36429055	S2_29055889	6	45	S6_14202383	S6_14380087	6.4	17.5	13.6	18.1	16.5	12.9
SY	2	245	S2_11771536	S2_206842	7	80	S7_15005750	S7_18208587	8.0	21.8	15.5	2.8	-3.6	27.2
SY	2	230	S2_20521571	S2_15866804	10	20	S10_6745618	S10_18754549	7.3	21.0	7.3	18.1	16.7	30.8
SY	2	10	S2_17095395	S2_16997696	11	55	S11_8456028	S11_24859368	5.5	25.8	19.4	-8.8	-14.2	41.5
DFF	3	110	S3_9468971	S3_8772530	3	200	S3_1763820	S3_3640530	5.4	10.5	3.2	-8.7	8.9	2.3
DFF	3	155	S3_24585116	S3_6422296	11	35	S11_13558980	S11_24859357	6.2	8.7	2.6	1.1	10.8	-9.6
GH	3	20	S3_21244595	S3_22913898	3	40	S3_17145449	S3_18154873	41.8	96.0	0.0	0.5	0.5	0.0
GH	3	20	S3_21244595	S3_22913898	4	5	S4_3592410	S4_2761907	30.7	69.0	0.0	0.4	0.0	-0.4
GH	3	20	S3_21244595	S3_22913898	5	30	S5_4692912	S5_1202286	8.9	61.5	0.2	-0.2	-0.2	0.1
GH	3	20	S3_21244595	S3_22913898	6	95	S6_6094182	S6_3447497	19.0	65.9	-0.1	-0.4	0.8	-0.4
GH	3	20	S3_21244595	S3_22913898	7	45	S7_2664466	S7_2709523	11.4	65.1	0.0	0.4	0.1	-0.4

Trait	Chr1	Pos1	LM1	RM1	Chr2	Pos2	LM2	RM2	LOD	PVE (%)	[aa]	[ad]	[da]	[dd]
GH	3	20	S3_21244595	S3_22913898	8	20	S8_648217	S8_5590896	17.4	66.4	0.0	0.4	0.1	-0.3
GH	3	20	S3_21244595	S3_22913898	9	50	S9_5329954	S9_1280000	14.9	66.2	0.0	0.8	-0.1	0.0
GH	3	20	S3_21244595	S3_22913898	10	10	S10_6745618	S10_18754549	15.6	65.8	0.2	-0.2	0.6	-0.6
GH	3	20	S3_21244595	S3_22913898	11	50	S11_10798039	S11_27887408	17.5	65.6	-0.2	-0.2	0.6	-0.2
SPC	3	190	S3_23950418	S3_23900756	11	30	S11_34926164	S11_14777000	5.3	21.2	-0.9	1.2	0.7	1.4
SW	3	25	S3_25402258	S3_18933167	3	160	S3_23347149	S3_5324878	7.1	21.6	-1.3	-0.5	-2.2	-1.2
SW	3	160	S3_23347149	S3_5324878	4	60	S4_5232181	S4_839628	8.8	23.7	-1.1	-0.1	-2.1	-1.5
SW	3	160	S3_23347149	S3_5324878	5	10	S5_4108250	S5_4692888	6.4	18.4	-0.2	3.1	0.0	2.2
SW	3	160	S3_23347149	S3_5324878	6	90	S6_6094182	S6_3447497	6.9	21.4	1.5	-1.9	0.9	-0.6
SW	3	160	S3_23347149	S3_5324878	7	55	S7_14683829	S7_19171518	6.3	18.3	1.0	-1.5	0.9	1.0
SW	3	90	S3_24127310	S3_24126743	8	120	S8_14899494	S8_15315643	5.4	15.7	0.3	2.5	-0.3	3.4
SW	3	235	S3_11414215	S3_19102565	9	5	S9_10221364	S9_10172092	9.4	26.3	1.3	-1.8	1.0	-0.3
SW	3	35	S3_17628375	S3_17145449	10	15	S10_6745618	S10_18754549	8.7	24.8	1.9	-1.6	0.7	-0.3
SW	3	160	S3_23347149	S3_5324878	11	165	S11_48519738	S11_22689710	7.2	22.5	1.3	-2.1	1.5	-0.7
SY	3	65	S3_1296061	S3_16632579	6	25	S6_18663394	S6_14282225	5.7	17.3	-12.0	-12.0	19.3	6.6
SY	3	105	S3_24127268	S3_3362024	7	10	S7_6043293	S7_1393902	5.2	15.4	-18.1	10.2	-12.4	16.9
SY	3	200	S3_1763820	S3_3640530	10	10	S10_6745618	S10_18754549	6.1	31.7	28.7	1.1	-6.5	16.0
SY	3	165	S3_7903058	S3_16519959	11	75	S11_26490677	S11_24186931	5.8	24.5	4.5	-6.8	-34.7	-16.0
GH	4	5	S4_3592410	S4_2761907	4	85	S4_2222481	S4_2761945	7.9	19.5	0.0	-0.1	-0.4	-0.4
GH	4	5	S4_3592410	S4_2761907	5	75	S5_4199522	S5_624899	10.1	24.4	0.1	0.0	-0.4	-0.4
GH	4	0	S4_3592410	S4_2761907	6	90	S6_6094182	S6_3447497	7.6	17.9	0.0	0.0	0.4	-0.5
GH	4	5	S4_3592410	S4_2761907	7	60	S7_19171518	S7_10467966	7.6	24.5	-0.1	0.1	0.4	-0.4
GH	4	5	S4_3592410	S4_2761907	8	50	S8_7083582	S8_6798921	13.8	28.6	-0.1	0.1	0.1	0.8
GH	4	5	S4_3592410	S4_2761907	10	40	S10_9867010	S10_18754605	15.5	32.7	0.0	0.1	0.2	-0.7
GH	4	5	S4_3592410	S4_2761907	11	165	S11_48519738	S11_22689710	9.2	26.4	0.0	0.0	-0.1	0.8
SW	4	15	S4_3592410	S4_2761907	4	55	S4_5573457	S4_5232181	7.1	21.3	-0.9	-0.7	3.0	2.9
SW	4	45	S4_5991509	S4_5573457	6	100	S6_6094182	S6_3447497	5.1	16.3	1.9	-1.9	1.0	-1.4
SW	4	90	S4_2222481	S4_2761945	8	0	S8_879539	S8_388862	5.2	20.3	-0.9	1.3	-0.3	-2.4

Trait	Chr1	Pos1	LM1	RM1	Chr2	Pos2	LM2	RM2	LOD	PVE (%)	[aa]	[ad]	[da]	[dd]
SW	4	55	S4_5573457	S4_5232181	10	75	S10_12011472	S10_17897318	8.8	18.8	-1.2	-1.0	-1.0	-0.6
SW	4	55	S4_5573457	S4_5232181	11	200	S11_3246235	S11_3725161	6.1	14.6	0.2	1.4	-0.1	0.9
SY	4	30	S4_9442019	S4_7998411	4	85	S4_2222481	S4_2761945	5.1	21.6	-7.2	-21.8	7.7	14.2
SY	4	30	S4_9442019	S4_7998411	10	10	S10_6745618	S10_18754549	6.9	22.6	-15.5	-7.7	14.4	11.0
GH	5	35	S5_4692912	S5_1202286	5	70	S5_4199522	S5_624899	8.9	24.2	0.1	0.2	-0.6	-0.4
GH	5	70	S5_4199522	S5_624899	11	170	S11_22689710	S11_4453854	6.3	18.9	0.0	-0.4	0.0	-0.2
SW	5	50	S5_1202286	S5_514608	6	100	S6_6094182	S6_3447497	5.2	17.0	1.1	-1.3	0.7	-0.8
SW	5	5	S5_3437906	S5_4108250	10	60	S10_4935601	S10_22177883	5.9	16.7	1.5	2.1	-0.9	-1.3
SW	5	0	S5_3437907	S5_3437906	11	45	S11_11249294	S11_25591691	5.5	14.6	1.3	1.4	-1.7	-1.5
SY	5	75	S5_4199522	S5_624899	10	10	S10_6745618	S10_18754549	7.4	23.3	-1.7	-1.8	-28.6	-23.8
DFF	6	90	S6_6094182	S6_3447497	7	85	S7_15005750	S7_18208587	5.4	10.7	-6.3	9.0	6.1	-13.1
GH	6	95	S6_6094182	S6_3447497	7	15	S7_6043293	S7_1393902	7.9	14.6	0.1	0.5	0.0	-0.3
GH	6	20	S6_21140545	S6_18663445	10	45	S10_9867010	S10_18754605	6.3	17.3	0.1	0.0	-0.6	-0.5
GH	6	95	S6_6094182	S6_3447497	11	20	S11_29782754	S11_40540329	7.0	20.6	0.2	0.6	0.1	-0.1
SW	6	35	S6_14335833	S6_14385396	6	45	S6_14202383	S6_14380087	8.2	17.3	1.5	3.0	-1.7	-0.2
SW	6	100	S6_6094182	S6_3447497	7	60	S7_19171518	S7_10467966	5.9	23.1	-0.8	-2.4	0.6	2.6
SW	6	95	S6_6094182	S6_3447497	8	15	S8_648217	S8_5590896	6.7	39.8	1.1	2.0	-1.5	-2.3
SW	6	95	S6_6094182	S6_3447497	9	10	S9_10221364	S9_10172092	5.0	17.7	-0.8	1.3	1.0	-1.5
SW	6	95	S6_6094182	S6_3447497	10	10	S10_6745618	S10_18754549	7.6	26.8	-1.8	1.6	1.4	-0.1
SW	6	95	S6_6094182	S6_3447497	11	45	S11_11249294	S11_25591691	6.6	19.3	1.3	0.8	-1.1	-0.3
SY	6	20	S6_21140545	S6_18663445	7	10	S7_6043293	S7_1393902	7.2	19.8	-15.5	16.2	-24.1	-0.3
SY	6	100	S6_6094182	S6_3447497	10	10	S10_6745618	S10_18754549	5.4	18.4	-6.8	-8.7	29.5	25.6
SY	6	40	S6_14282200	S6_14335733	11	75	S11_26490677	S11_24186931	6.4	14.7	14.1	18.2	16.2	8.5
DFF	7	85	S7_15005750	S7_18208587	9	5	S9_10221364	S9_10172092	5.7	10.5	4.9	6.3	-5.2	-15.1
GH	7	30	S7_1393902	S7_2664466	7	35	S7_1393902	S7_2664466	8.1	21.0	0.1	-0.3	0.2	-0.9
GH	7	65	S7_19171518	S7_10467966	8	95	S8_18579673	S8_15137210	5.7	18.4	0.3	-0.1	-0.1	0.3
GH	7	30	S7_1393902	S7_2664466	10	10	S10_6745618	S10_18754549	6.7	20.5	-0.1	-0.1	0.3	-0.4
SW	7	65	S7_19171518	S7_10467966	7	70	S7_10467966	S7_15005750	6.3	30.2	-0.8	0.9	-3.8	1.1

Trait	Chr1	Pos1	LM1	RM1	Chr2	Pos2	LM2	RM2	LOD	PVE (%)	[aa]	[ad]	[da]	[dd]
SW	7	5	S7_6043293	S7_1393902	10	60	S10_4935601	S10_22177883	6.1	17.6	-1.1	-2.2	-1.3	-1.2
SY	7	75	S7_10467966	S7_15005750	10	20	S10_6745618	S10_18754549	7.2	17.7	-16.5	-3.4	12.5	14.9
SY	7	10	S7_6043293	S7_1393902	11	195	S11_3724414	S11_366844	8.5	24.1	-1.0	35.6	8.6	-28.8
GH	8	55	S8_7083582	S8_6798921	8	60	S8_6798921	S8_19450430	11.7	24.7	-0.4	0.0	-0.6	0.2
GH	8	50	S8_7083582	S8_6798921	10	45	S10_9867010	S10_18754605	7.5	22.8	0.1	0.1	-0.5	-0.6
SW	8	50	S8_7083582	S8_6798921	10	10	S10_6745618	S10_18754549	7.9	26.4	1.1	-0.9	2.4	-1.8
SW	8	15	S8_648217	S8_5590896	11	50	S11_10798039	S11_27887408	6.0	35.4	0.9	-1.7	1.5	-1.7
SY	8	20	S8_648217	S8_5590896	10	10	S10_6745618	S10_18754549	5.1	26.9	6.9	15.4	14.9	32.1
SY	8	60	S8_6798921	S8_19450430	11	55	S11_8456028	S11_24859368	5.0	20.7	-25.7	7.9	-10.2	39.4
GH	9	55	S9_5329954	S9_1280000	9	70	S9_1280107	S9_1272426	11.8	25.6	0.1	-0.4	0.6	-0.2
GH	9	70	S9_1280107	S9_1272426	10	40	S10_9867010	S10_18754605	11.3	26.1	0.0	0.3	0.3	-0.2
SW	9	25	S9_7756050	S9_8995134	9	35	S9_7755937	S9_7993111	6.8	16.3	0.1	-2.4	0.0	2.5
SW	9	25	S9_7756050	S9_8995134	10	120	S10_17800762	S10_20225996	8.9	19.0	1.0	1.2	-1.0	-2.0
SW	9	45	S9_5329954	S9_1280000	11	60	S11_2583306	S11_36601967	7.1	19.4	1.0	1.2	-0.7	-1.7
SY	9	20	S9_10003418	S9_8998000	10	5	S10_6745618	S10_18754549	7.3	22.7	-5.9	0.9	-30.8	-16.4
GH	10	35	S10_9867010	S10_18754605	10	40	S10_9867010	S10_18754605	13.6	27.3	0.1	0.1	0.2	-0.6
GH	10	10	S10_6745618	S10_18754549	11	65	S11_25591551	S11_36998432	11.7	23.0	0.2	0.2	0.0	-0.5
SW	10	15	S10_6745618	S10_18754549	10	75	S10_12011472	S10_17897318	8.9	23.3	1.5	0.4	-1.4	-0.8
SW	10	75	S10_12011472	S10_17897318	11	165	S11_48519738	S11_22689710	8.7	19.5	1.4	-1.2	1.4	-1.2
SY	10	15	S10_6745618	S10_18754549	10	45	S10_9867010	S10_18754605	6.7	33.1	-23.0	8.8	3.0	22.4
SY	10	10	S10_6745618	S10_18754549	11	180	S11_9114357	S11_2019429	7.7	27.1	7.3	-18.8	2.5	-38.0
GH	11	165	S11_48519738	S11_22689710	11	170	S11_22689710	S11_4453854	7.1	16.0	-0.2	-0.1	0.3	0.3
SW	11	60	S11_2583306	S11_36601967	11	125	S11_7565026	S11_20087622	7.1	19.4	-0.6	-1.3	-1.6	-1.1
SY	11	55	S11_8456028	S11_24859368	11	160	S11_29845620	S11_23864521	5.4	20.0	16.3	-7.2	0.3	19.7
Pop4 (ICP 8863 × ICPL 87119)														
DFF	1	25	S1_1285564	S1_16743053	1	120	S1_1954810	S1_12641799	5.4	16.2	3.9	-7.7	3.6	-0.3
DFF	1	75	S1_1158266	S1_12641760	3	145	S3_4949379	S3_15768334	5.3	19.2	1.8	-1.0	-7.4	5.5
SPC	1	0	S1_3518364	S1_8912598	10	165	S10_11797822	S10_22177616	5.3	9.8	0.5	-0.5	0.8	0.5

Trait	Chr1	Pos1	LM1	RM1	Chr2	Pos2	LM2	RM2	LOD	PVE (%)	[aa]	[ad]	[da]	[dd]
SW	1	105	S1_2823169	S1_11795390	3	105	S3_27536189	S3_13210310	5.3	21.9	0.6	-0.4	-0.2	-1.5
SW	1	110	S1_9402663	S1_11824695	4	55	S4_3978352	S4_3978307	5.1	23.8	-0.2	-1.1	-0.8	0.3
SW	1	110	S1_9402663	S1_11824695	8	50	S8_1870690	S8_14893200	5.1	20.9	0.0	-1.2	0.4	-0.4
SW	1	45	S1_11236615	S1_9402646	11	190	S11_10379800	S11_39387203	5.2	19.6	0.1	0.3	-0.8	-1.1
SY	1	5	S1_3518364	S1_8912598	11	200	S11_10379800	S11_39387203	6.3	23.8	1.8	-20.4	16.8	-63.8
SY	1	45	S1_11236615	S1_9402646	2	20	S2_5465513	S2_27251002	5.3	17.8	-1.0	13.5	16.0	-44.4
SY	1	15	S1_15951980	S1_15951983	7	45	S7_11292795	S7_15538705	5.3	12.4	-20.9	-24.9	23.1	-7.1
SY	1	15	S1_15951980	S1_15951983	8	10	S8_4870225	S8_17923633	5.2	12.0	-13.9	-19.3	38.5	10.4
DFF	2	105	S2_20786266	S2_3909322	3	95	S3_1820861	S3_18837767	5.9	15.5	2.9	0.8	11.2	5.1
DFF	2	170	S2_31369072	S2_2636840	6	100	S6_11368993	S6_16630543	5.6	19.8	0.3	5.0	-8.1	9.0
DFF	2	0	S2_19392681	S2_31090530	7	170	S7_7547477	S7_2556783	5.7	19.1	0.5	6.1	3.9	4.6
SPC	2	5	S2_31090530	S2_11172947	6	120	S6_8998640	S6_22745176	5.3	17.0	0.1	0.4	-1.2	0.7
SW	2	40	S2_10335056	S2_2989918	11	175	S11_41966756	S11_2739522	6.2	24.4	0.2	0.1	-1.4	-0.7
SW	2	10	S2_11172947	S2_11585386	4	100	S4_3982439	S4_10981441	5.1	25.4	0.4	0.5	-0.9	1.0
SW	2	45	S2_2989945	S2_28067338	6	95	S6_21912913	S6_11368997	5.6	21.0	-0.1	0.2	0.7	-1.8
SW	2	125	S2_12561939	S2_16904395	8	90	S8_4088164	S8_11725020	5.6	25.0	-0.1	-0.3	-1.1	1.0
SY	2	20	S2_5465513	S2_27251002	6	75	S6_12492736	S6_19839881	5.1	14.1	3.8	11.6	-6.9	-48.9
SY	2	105	S2_20786266	S2_3909322	8	100	S8_12763827	S8_2545645	6.1	19.6	3.8	4.0	-25.2	-55.6
DFF	3	110	S3_517899	S3_5034170	4	60	S4_3978352	S4_3978307	6.2	16.7	5.3	7.1	-2.0	6.2
DFF	3	0	S3_17585835	S3_6422190	5	75	S5_1033048	S5_2154363	5.2	14.7	0.6	9.8	-5.7	-3.9
DFF	3	95	S3_1820861	S3_18837767	10	80	S10_14607250	S10_15235469	5.7	16.2	-2.3	7.2	-6.1	0.2
SW	3	130	S3_3207601	S3_28801734	7	40	S7_5257435	S7_19515938	5.5	23.7	-0.5	0.0	-0.2	-1.7
SW	4	55	S4_3978352	S4_3978307	4	95	S4_3982439	S4_10981441	5.5	25.6	-0.1	0.7	-1.0	1.7
SW	4	45	S4_3978352	S4_3978307	7	145	S7_4934324	S7_16759426	7.0	14.2	0.3	-0.8	0.8	0.1
SW	4	50	S4_3978352	S4_3978307	10	55	S10_7592635	S10_16594094	5.2	17.5	0.1	-0.8	0.1	-1.2
SW	4	30	S4_3978304	S4_11052197	11	195	S11_10379800	S11_39387203	5.6	19.5	-0.5	-0.6	-0.6	-0.3
SY	4	60	S4_3978352	S4_3978307	7	50	S7_17391305	S7_629308	5.2	15.1	-6.9	-19.4	-39.9	-53.8
SW	5	55	S5_4439127	S5_1033048	6	165	S6_19397317	S6_22274548	5.2	14.6	-0.4	-0.9	0.0	0.1

Trait	Chr1	Pos1	LM1	RM1	Chr2	Pos2	LM2	RM2	LOD	PVE (%)	[aa]	[ad]	[da]	[dd]
SY	5	5	S5_2134834	S5_2133997	8	100	S8_12763827	S8_2545645	7.2	21.1	-5.2	8.6	-47.6	-29.4
DFF	6	100	S6_11368993	S6_16630543	6	135	S6_18650949	S6_10118855	5.1	23.1	0.8	-4.7	6.6	11.6
DFF	6	100	S6_11368993	S6_16630543	7	165	S7_7547426	S7_7547477	5.4	12.0	-3.6	1.1	6.5	3.3
DFF	6	185	S6_9119076	S6_1641600	11	155	S11_26961748	S11_11799678	5.5	19.5	-6.6	4.4	2.8	5.7
SPC	6	95	S6_21912913	S6_11368997	7	40	S7_5257435	S7_19515938	6.3	30.5	0.0	1.2	-0.2	1.5
SPC	6	120	S6_8998640	S6_22745176	8	75	S8_4817492	S8_18097152	5.5	18.6	0.3	-0.8	-0.2	1.5
SPC	6	145	S6_4528744	S6_11344426	10	25	S10_8682310	S10_22432012	5.0	19.1	0.4	-0.7	-0.7	0.7
SW	6	95	S6_21912913	S6_11368997	7	40	S7_5257435	S7_19515938	6.1	18.6	0.4	0.6	-0.8	-1.1
SW	6	10	S6_5168768	S6_21993984	6	95	S6_21912913	S6_11368997	5.3	22.1	0.1	-0.1	0.8	-1.8
SW	6	95	S6_21912913	S6_11368997	10	150	S10_11797843	S10_17661180	5.3	21.7	0.1	1.1	-0.1	-1.4
SW	6	95	S6_21912913	S6_11368997	11	190	S11_10379800	S11_39387203	5.1	19.6	0.1	-0.5	0.6	1.2
SW	7	40	S7_5257435	S7_19515938	8	5	S8_4870225	S8_17923633	5.5	25.3	0.0	1.1	0.2	1.4
SY	8	100	S8_12763827	S8_2545645	8	140	S8_19001637	S8_19307650	5.4	16.4	0.5	-31.6	18.2	-28.0
SY	8	100	S8_12763827	S8_2545645	10	125	S10_21275558	S10_8446246	6.1	16.0	1.1	-38.8	13.8	-55.8
SY	8	115	S8_12196669	S8_8905244	11	200	S11_10379800	S11_39387203	5.5	23.1	1.3	-1.5	21.3	-68.0
SPC	10	130	S10_12572056	S10_11360684	11	175	S11_41966756	S11_2739522	5.2	21.4	-0.3	0.6	-1.2	1.7
SPC	10	115	S10_16591318	S10_19313155	10	165	S10_11797822	S10_22177616	5.5	12.8	-0.1	0.0	1.1	0.7
SY	10	140	S10_11129172	S10_22177741	11	35	S11_29910199	S11_1427973	6.1	14.3	-26.5	-16.0	4.6	30.7
DFF	11	30	S11_6866243	S11_22800082	11	185	S11_27825781	S11_27703059	5.1	30.1	-0.6	-4.0	2.3	14.1
SPC	11	10	S11_9655513	S11_11782549	11	25	S11_39259070	S11_5870873	5.2	28.9	-0.3	0.2	0.7	2.4
SW	11	175	S11_41966756	S11_2739522	11	190	S11_10379800	S11_39387203	5.7	24.8	0.6	0.9	-0.5	1.0
Pop5 (ICP 5529 × ICP 11605)														
GH	1	80	S1_17462230	s1_16873606	5	45	S5_4199096	S5_3228176	5.1	10.6	0.2	0.3	0.3	0.1
SW	1	90	S1_16910575	S1_17478283	2	15	S2_4089442	S2_36164833	5.1	13.6	1.0	-1.4	1.2	-0.9
SW	1	90	S1_16910575	S1_17478283	7	175	S7_14683829	S7_14588865	5.2	14.6	1.1	-1.6	0.9	-1.2
SW	1	90	S1_16910575	S1_17478283	11	95	S11_42473528	S11_40289708	6.9	13.5	-1.3	-1.2	-1.3	-0.9
SW	1	95	S1_17486758	S1_17365797	3	125	S3_5052534	S3_8600411	5.5	14.7	1.0	-1.5	1.4	-1.4
SW	1	35	s1_4415753	S1_4839845	1	90	S1_16910575	S1_17478283	6.2	11.5	0.0	1.1	-0.6	-0.5

Trait	Chr1	Pos1	LM1	RM1	Chr2	Pos2	LM2	RM2	LOD	PVE (%)	[aa]	[ad]	[da]	[dd]
SY	1	50	S1_1575466	S1_12652912	3	85	S3_23698867	S3_8011694	5.5	17.4	22.1	-18.8	-21.4	41.8
SY	1	80	S1_17462230	s1_16873606	2	5	S2_6500923	S2_36141669	5.6	22.9	2.5	-38.8	5.3	-35.3
SY	1	75	S1_3905217	S1_17462230	5	85	S5_2154598	S5_3299149	7.2	32.7	-33.8	2.8	1.3	57.2
SY	1	75	S1_3905217	S1_17462230	8	160	S8_17090863	S8_5491195	7.2	33.3	4.4	-44.5	-12.6	-46.8
SY	1	75	S1_3905217	S1_17462230	9	30	S9_7304246	S9_5330124	5.6	27.2	-0.2	-29.3	0.6	-55.8
SY	1	75	S1_3905217	S1_17462230	11	150	S11_772476	S11_17940886	5.9	31.5	23.6	15.4	30.5	-20.0
SY	1	55	S1_435014	S1_14036692	1	60	S1_435014	S1_14036692	6.8	29.6	-11.8	-24.6	71.9	-18.8
SY	1	20	S1_5944791	S1_5173345	6	55	S6_6094182	S6_3312026	6.7	40.0	-10.5	-11.0	45.6	-34.4
DFF	2	10	S2_16974864	S2_6623704	7	175	S7_14683829	S7_14588865	5.1	21.2	-3.3	-3.4	8.7	1.8
GH	2	140	S2_13395941	S2_13388912	4	50	S4_5573457	S4_7149747	5.4	16.6	0.1	0.5	0.1	-0.3
GH	2	130	s2_16726731	S2_16216536	2	165	S2_36104004	S2_7680557	5.5	26.7	0.1	0.3	0.3	-0.6
GH	2	170	S2_36104004	S2_7680557	3	65	S3_14618126	S3_22872410	6.7	22.6	0.2	-0.1	-0.5	-0.3
GH	2	160	S2_36104004	S2_7680557	6	95	S6_14282225	S6_14311546	5.7	13.1	-0.2	0.6	0.0	-0.3
GH	2	160	S2_36104004	S2_7680557	8	70	S8_16623818	S8_8700231	5.2	22.3	-0.2	0.6	-0.2	-0.2
GH	2	50	S2_9110747	S2_12221357	5	40	S5_2081547	S5_4199096	5.9	14.9	-0.1	0.1	-0.4	-0.7
GH	2	50	S2_9110747	S2_12221357	7	110	S7_6037064	S7_2639387	8.9	22.0	-0.2	-0.1	0.3	0.2
GH	2	50	S2_9110747	S2_12221357	11	70	S11_43548291	S11_32240905	6.9	21.7	0.3	-0.1	0.0	0.4
SW	2	10	S2_16974864	S2_6623704	11	25	S11_6210775	S11_39507811	5.3	24.4	0.3	-1.2	1.0	-1.3
SY	2	10	S2_16974864	S2_6623704	3	115	S3_14754306	S3_8040710	5.3	19.0	0.7	31.2	-19.7	-46.9
SY	2	10	S2_16974864	S2_6623704	7	175	S7_14683829	S7_14588865	5.7	27.9	17.8	-47.8	-14.1	56.9
SY	2	25	S2_30868559	S2_3713697	6	70	S6_14548839	S6_12512558	7.0	28.3	-1.0	13.9	46.7	-68.2
SY	2	40	S2_31385744	s2_1201138_	11	160	S11_38144723	S11_27887800	6.6	25.7	3.2	-0.9	-60.1	40.4
SY	2	5	S2_6500923	S2_36141669	8	105	S8_9267520	S8_9779781	6.9	31.4	-2.5	-9.8	66.5	-41.5
DFF	3	80	S3_8195933	S3_3563061	11	70	S11_43548291	S11_32240905	5.5	23.6	-4.4	-4.5	-3.7	12.3
GH	3	35	s3_20698771	S3_18430894	3	65	S3_14618126	S3_22872410	16.6	74.8	-0.1	-0.1	-0.6	-0.6
GH	3	35	s3_20698771	S3_18430894	4	25	S4_1710877	s4_496463	15.3	73.8	0.1	-0.1	0.7	-0.6
GH	3	35	s3_20698771	S3_18430894	5	135	S5_4199522	S5_3988054	8.7	74.3	-0.1	-0.2	0.2	-0.6
GH	3	35	s3_20698771	S3_18430894	6	65	S6_16310378	S6_14551548	15.6	74.2	0.2	-0.2	0.5	-0.6

Trait	Chr1	Pos1	LM1	RM1	Chr2	Pos2	LM2	RM2	LOD	PVE (%)	[aa]	[ad]	[da]	[dd]
GH	3	35	s3_20698771	S3_18430894	8	90	S8_7083922	S8_8700211	12.5	74.1	0.4	-0.4	0.0	0.1
GH	3	35	s3_20698771	S3_18430894	9	65	S9_1280107	S9_7212593	5.8	69.4	-0.1	-0.1	0.0	-0.3
GH	3	35	s3_20698771	S3_18430894	11	165	S11_35533615	S11_24152330	10.2	73.0	0.2	-0.2	0.5	-0.6
GH	3	25	S3_22234078	S3_16681929	7	20	S7_19090687	S7_13036645	8.8	33.7	-0.2	0.1	0.6	-0.6
GH	3	90	S3_8011694	S3_22090943	10	45	S10_5310960	S10_4472034	5.3	16.4	0.2	0.0	-0.1	0.5
SW	3	20	S3_24127385	S3_21274904	3	100	S3_10301855	S3_7318897	5.2	16.4	1.3	1.8	-0.4	-1.3
SW	3	20	S3_24127385	S3_21274904	10	60	S10_4472034	S10_234727	5.6	19.6	1.1	0.3	-0.5	0.5
SW	3	15	S3_28538775	S3_21244595	11	95	S11_42473528	S11_40289708	5.8	15.8	1.0	1.7	-1.2	-0.3
SY	3	100	S3_10301855	S3_7318897	11	140	S11_31114457	S11_28353104	8.7	26.8	-15.8	-61.7	37.2	-5.3
SY	3	40	S3_18430894	S3_18154848	8	155	S8_9779799	S8_17090863	5.4	31.8	-13.3	52.3	-20.5	-45.5
SY	3	130	S3_2106166	S3_6531705	6	125	S6_18245488	S6_18663445	5.6	26.1	2.3	-34.0	13.6	-79.5
SY	3	85	S3_23698867	S3_8011694	3	105	S3_925660	S3_8994380	6.8	22.4	-31.1	-26.1	27.4	35.3
SY	3	155	S3_29089318	S3_517761	7	115	S7_16759426	S7_18208587	5.8	17.4	-33.9	-18.7	42.7	20.3
DFF	4	40	S4_3887219	S4_3355054	11	80	S11_18575052	S11_38654219	7.0	22.2	4.8	4.3	-2.7	10.8
GH	4	25	S4_1710877	s4_496463	8	5	S8_18016282	S8_879539	5.6	18.2	0.0	0.5	0.0	-0.4
GH	4	25	S4_1710877	s4_496463	11	10	S11_36998432	S11_26636997	5.5	17.3	-0.2	-0.1	0.3	0.2
GH	4	30	s4_428893	S4_2900796	6	65	S6_16310378	S6_14551548	5.2	10.9	0.3	-0.2	-0.2	0.1
SPC	4	10	S4_1867372	S4_2222488	5	90	S5_2154598	S5_3299149	6.9	15.4	-0.2	-0.5	1.0	0.7
SPC	4	40	S4_3887219	S4_3355054	7	175	S7_14683829	S7_14588865	5.2	15.0	0.2	-0.2	0.4	-1.7
SW	4	25	S4_1710877	s4_496463	7	70	S7_7974084	S7_2627909	5.1	19.9	-0.9	-0.3	-0.6	-2.3
SW	4	25	S4_1710877	s4_496463	11	120	S11_2412606	S11_5977933	5.1	19.5	-0.7	-2.1	-1.6	0.3
SY	4	5	S4_2313990	S4_1867372	11	165	S11_35533615	S11_24152330	5.3	20.8	-22.7	-6.4	20.4	39.9
SY	4	30	s4_428893	S4_2900796	7	20	S7_19090687	S7_13036645	5.1	17.4	30.5	33.6	35.3	10.2
DFF	5	120	S5_703897	S5_4199522	11	135	S11_13945965	S11_24152343	5.4	23.1	-1.3	-0.4	1.2	-15.9
GH	5	115	S5_2827511	S5_703897	11	25	S11_6210775	S11_39507811	8.5	25.5	-0.4	0.2	0.1	0.1
GH	5	30	S5_3908353	S5_2511713	6	140	S6_22076489	S6_15818390	7.4	19.2	-0.3	0.1	-0.1	0.6
GH	5	45	S5_4199096	S5_3228176	5	70	S5_2154719	S5_3512197	6.4	18.1	-0.1	0.2	-0.5	-0.2
GH	5	45	S5_4199096	S5_3228176	10	15	S10_7783201	S10_7567880	5.6	11.1	-0.2	0.3	-0.1	0.4

Trait	Chr1	Pos1	LM1	RM1	Chr2	Pos2	LM2	RM2	LOD	PVE (%)	[aa]	[ad]	[da]	[dd]
GH	5	130	S5_4199522	S5_3988054	7	100	S7_10731962	S7_15294781	8.6	26.8	0.0	0.3	-0.1	-0.6
GH	5	130	S5_4199522	S5_3988054	8	10	S8_18016282	S8_879539	8.4	28.0	0.0	0.4	0.1	-0.6
SPC	5	105	S5_3512215	S5_3512203	7	65	S7_19133038	S7_19133012	5.8	9.5	0.2	-0.2	-0.1	-1.2
SPC	5	85	S5_2154598	S5_3299149	11	55	S11_4453854	S11_4725362	5.2	21.2	0.3	0.7	0.4	1.9
SW	5	115	S5_2827511	S5_703897	11	95	S11_42473528	S11_40289708	6.0	19.6	0.1	0.2	1.3	3.0
SY	5	105	S5_3512215	S5_3512203	11	160	S11_38144723	S11_27887800	5.5	16.9	-6.0	0.8	46.6	-31.5
SY	5	65	S5_2154419	S5_3511745	6	70	S6_14548839	S6_12512558	5.9	35.1	30.5	4.8	-11.4	79.8
SY	5	65	S5_2154419	S5_3511745	9	25	S9_7304246	S9_5330124	5.0	22.0	-5.5	-39.6	-26.0	-55.6
SY	5	75	S5_2154719	S5_3512197	5	95	S5_3299149	S5_5120324	5.0	18.0	-35.5	-0.1	-12.3	44.8
SY	5	125	S5_4199522	S5_3988054	7	170	S7_14130607	S7_14683829	6.2	25.3	-20.9	26.7	-27.0	68.5
DFF	6	45	S6_60533	S6_3186747	6	110	S6_18663394	S6_17420283	5.1	14.7	1.5	2.8	-7.1	-3.9
GH	6	80	S6_12512558	S6_3630897	8	10	S8_18016282	S8_879539	6.8	28.5	-0.1	0.7	-0.2	-0.2
GH	6	70	S6_14548839	S6_12512558	6	80	S6_12512558	S6_3630897	6.8	19.3	-0.4	0.1	0.0	0.5
GH	6	70	S6_14548839	S6_12512558	7	145	S7_4126324	S7_4191819	5.9	19.9	0.1	0.4	0.1	-0.1
GH	6	15	S6_3630463	S6_6794468	11	85	S11_23930022	S11_45876012	6.4	27.6	0.0	-0.6	0.3	-0.7
SW	6	95	S6_14282225	S6_14311546	10	65	S10_4472034	S10_234727	5.1	21.8	-0.9	1.0	0.2	0.4
SW	6	15	S6_3630463	S6_6794468	11	25	S11_6210775	S11_39507811	7.3	30.0	1.2	-1.6	0.4	-1.3
SY	6	125	S6_18245488	S6_18663445	8	105	S8_9267520	S8_9779781	6.8	32.6	-3.9	7.2	56.5	-80.4
SY	6	125	S6_18245488	S6_18663445	10	75	S10_4472034	S10_234727	5.5	17.5	-4.1	-5.9	23.3	50.6
SY	6	120	S6_18322873	S6_18322737	7	25	S7_360074	S7_396455	5.2	12.4	16.9	-27.3	-13.0	13.9
SY	6	50	S6_3186747	S6_6094182	11	160	S11_38144723	S11_27887800	8.6	32.3	-9.0	1.9	45.3	-76.8
SY	6	55	S6_6094182	S6_3312026	6	60	S6_3312026	S6_16310378	5.9	33.3	7.5	36.8	-4.9	-73.3
GH	7	15	S7_19090687	S7_13036645	9	10	S9_10172092	S9_7756050	5.7	13.3	0.3	0.1	0.0	0.2
GH	7	55	S7_4753261	S7_16343301	8	10	S8_18016282	S8_879539	6.4	21.2	0.1	-0.6	-0.2	-0.3
GH	7	75	S7_566440	S7_5435193	10	30	S10_16425032	S10_5310960	7.3	18.8	-0.1	0.3	0.1	0.6
GH	7	130	S7_5677691	S7_2549635	11	195	S11_33160406	S11_23457576	7.1	16.7	-0.3	0.0	0.0	0.4
GH	7	110	S7_6037064	S7_2639387	7	160	S7_6897487	S7_6219852	7.2	20.7	-0.3	0.1	0.0	0.4
SW	7	155	S7_10971518	S7_6897487	9	65	S9_1280107	S9_7212593	5.6	23.0	-0.8	0.6	0.6	-2.5

Trait	Chr1	Pos1	LM1	RM1	Chr2	Pos2	LM2	RM2	LOD	PVE (%)	[aa]	[ad]	[da]	[dd]
SW	7	115	S7_16759426	S7_18208587	11	190	S11_33160406	S11_23457576	6.1	23.4	-1.1	-1.5	-1.2	-0.6
SY	7	155	S7_10971518	S7_6897487	8	130	S8_14208514	s8_14254343	6.6	23.9	-22.5	-38.5	43.9	34.7
SY	7	170	S7_14130607	S7_14683829	9	45	S9_7212583	S9_1662492	7.6	15.9	-12.3	-26.3	-13.5	35.8
SY	7	20	S7_19090687	S7_13036645	11	160	S11_38144723	S11_27887800	7.9	25.7	-39.9	12.2	-12.4	62.6
SY	7	110	S7_6037064	S7_2639387	7	175	S7_14683829	S7_14588865	6.9	31.4	6.7	22.7	55.1	-51.9
GH	8	90	S8_7083922	S8_8700211	10	70	S10_4472034	S10_234727	7.1	24.2	0.2	-0.5	-0.2	-0.3
GH	8	85	S8_7083922	S8_8700211	11	80	S11_18575052	S11_38654219	7.0	24.3	0.3	0.0	0.0	0.6
GH	8	60	S8_8700222	S8_5616255	8	85	S8_7083922	S8_8700211	5.8	19.0	-0.4	0.0	0.0	0.5
SW	8	5	S8_18016282	S8_879539	11	25	S11_6210775	S11_39507811	5.9	26.8	1.1	-0.8	0.8	0.0
SY	8	130	S8_14208514	s8_14254343	11	160	S11_38144723	S11_27887800	6.8	30.2	6.6	-1.9	-58.8	79.1
SY	8	105	S8_9267520	S8_9779781	8	120	S8_10333523	S8_14208514	5.5	28.6	-22.6	-37.6	30.2	47.9
GH	9	50	S9_7212583	S9_1662492	11	70	S11_43548291	S11_32240905	5.6	17.7	0.3	-0.1	0.0	0.2
SW	9	40	S9_5330124	S9_5329954	11	25	S11_6210775	S11_39507811	6.1	23.0	-0.2	0.2	-2.0	2.1
SY	9	40	S9_5330124	S9_5329954	11	160	S11_38144723	S11_27887800	8.1	24.5	13.2	7.5	64.5	-49.5
GH	10	30	S10_16425032	S10_5310960	11	80	S11_18575052	S11_38654219	5.7	19.3	0.3	-0.1	-0.1	0.4
SW	10	65	S10_4472034	S10_234727	11	25	S11_6210775	S11_39507811	5.4	29.0	1.2	-1.2	0.6	0.2
SY	10	65	S10_4472034	S10_234727	11	160	S11_38144723	S11_27887800	6.8	32.6	39.5	-26.1	-13.3	68.7
GH	11	10	S11_36998432	S11_26636997	11	80	S11_18575052	S11_38654219	7.4	20.9	0.1	0.2	0.5	0.0
SW	11	50	S11_42065843	S11_12498183	11	95	S11_42473528	S11_40289708	6.3	17.0	-1.5	-0.3	-1.4	-0.7
SY	11	25	S11_6210775	S11_39507811	11	135	S11_13945965	S11_24152343	6.5	22.8	-21.4	-48.1	23.3	35.3

Supplementary Figure S1. Genetic and QTL maps for Pop1 (ICP 11605 × ICP 14209). Markers are shown on right side of the linkage group while map distances are indicated on left side. QTLs for the different traits are indicated by different colored bars with red, purple, blue, black and green showing QTLs for seed protein content, seed weight, seed yield, growth habit and days to first flowering, respectively.

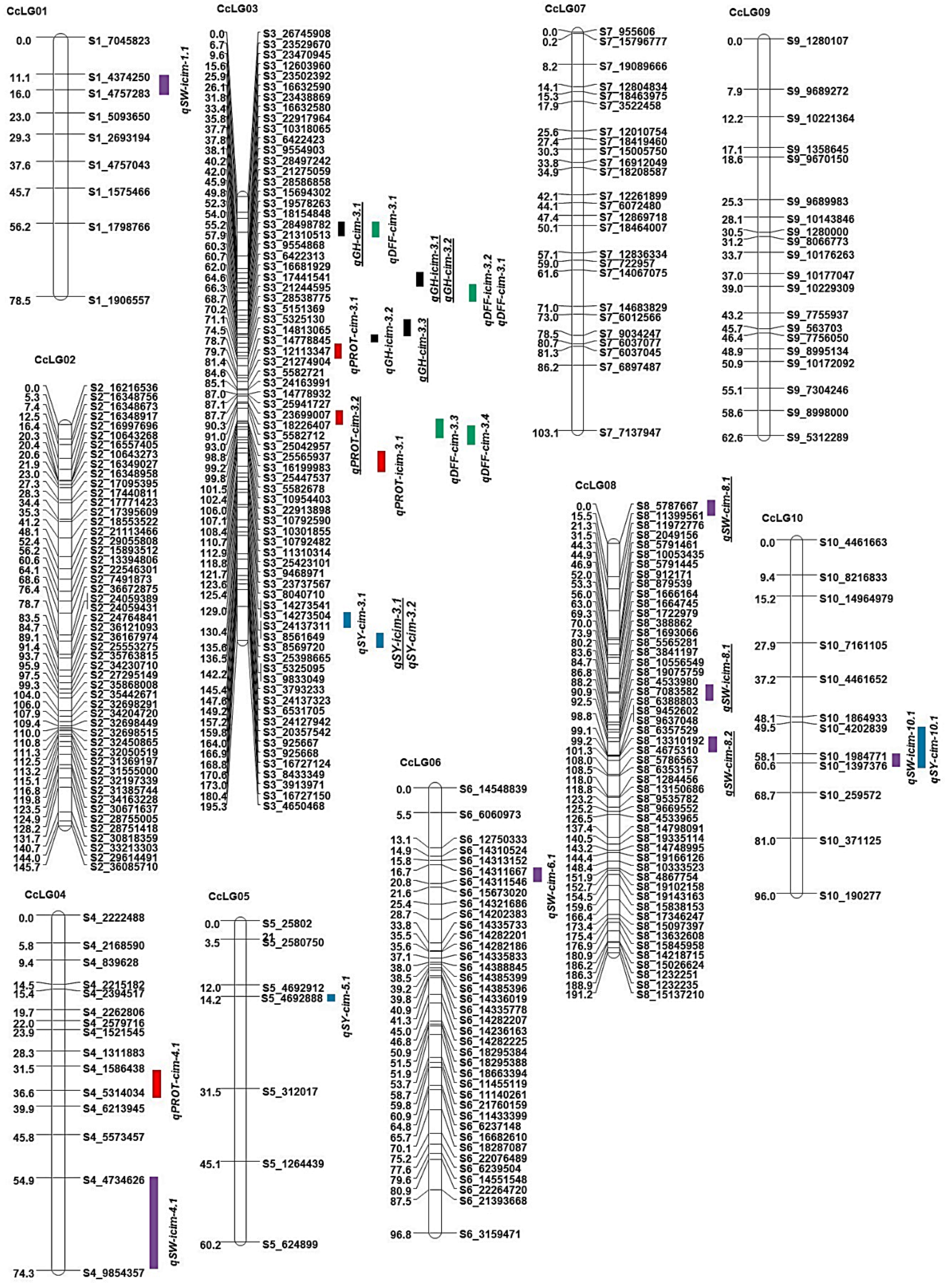

Supplementary Figure S1 (continued)

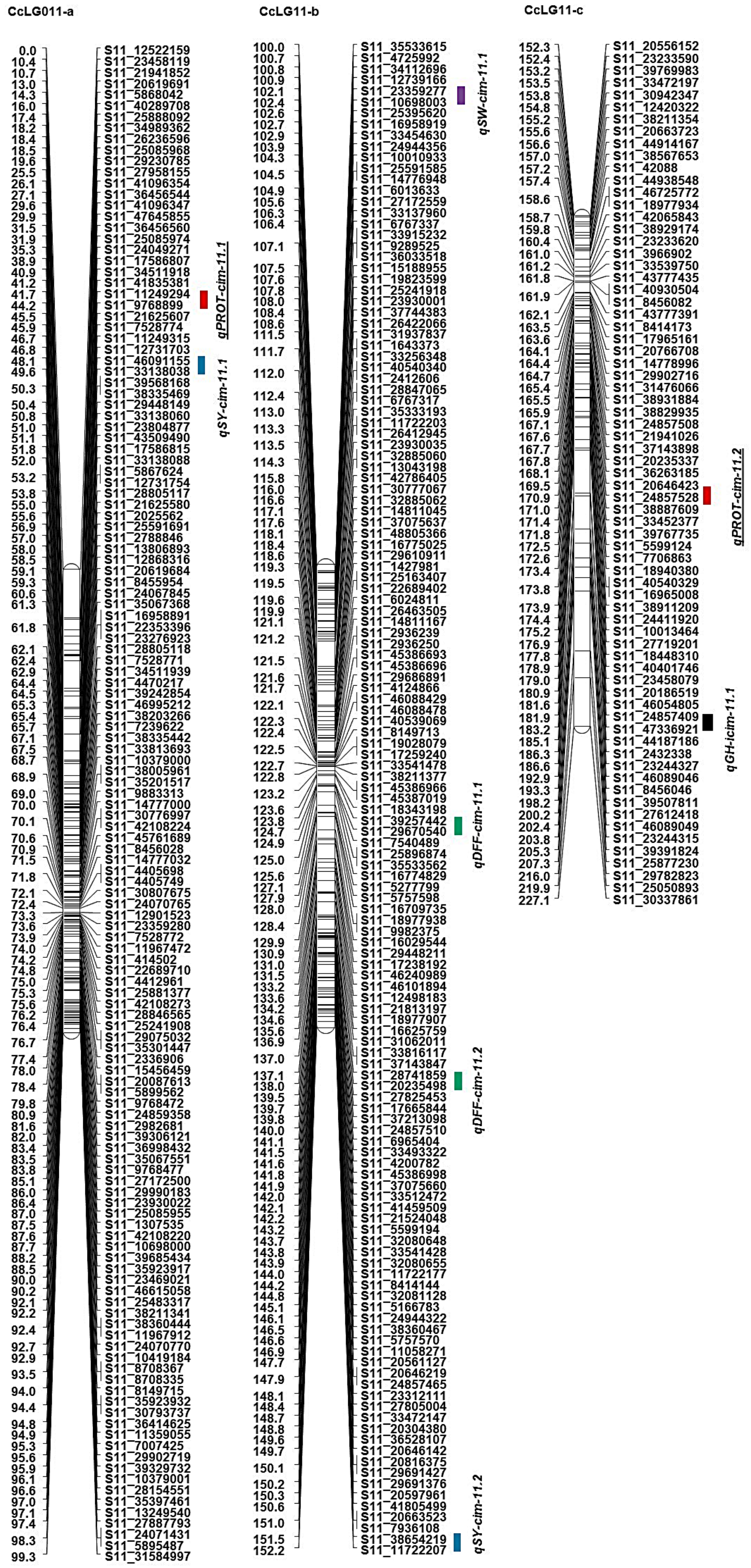

Supplementary Figure S2. Genetic and QTL maps for Pop2 (ICP 8863 × ICP 11605). Markers are shown on right side of the linkage group while map distances are indicated on left side. QTLs for the different traits are indicated by different colored bars with red, purple, blue, black and green showing QTLs for seed protein content, seed weight, seed yield, growth habit and days to first flowering, respectively.

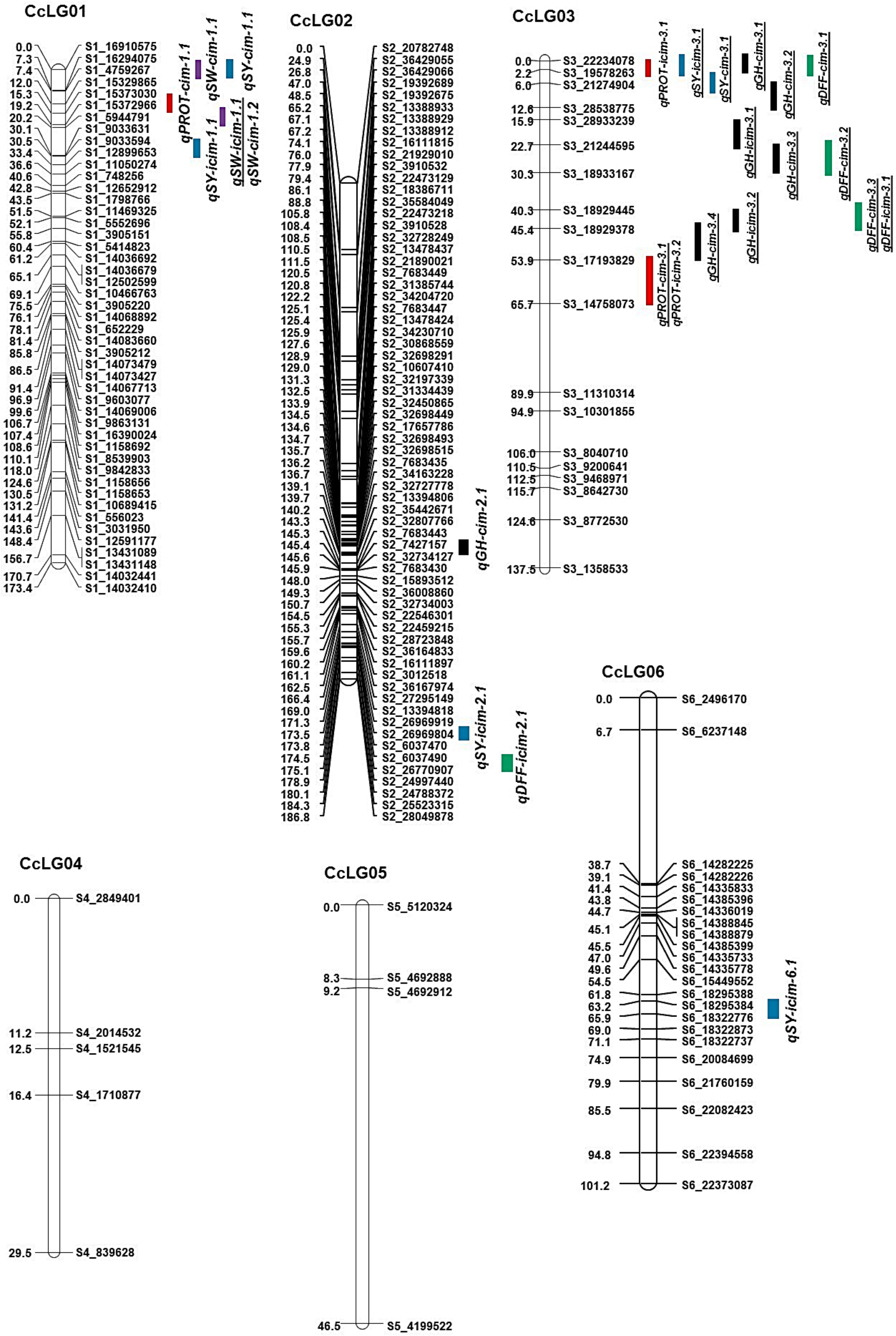

Supplementary Figure S2 (continued)

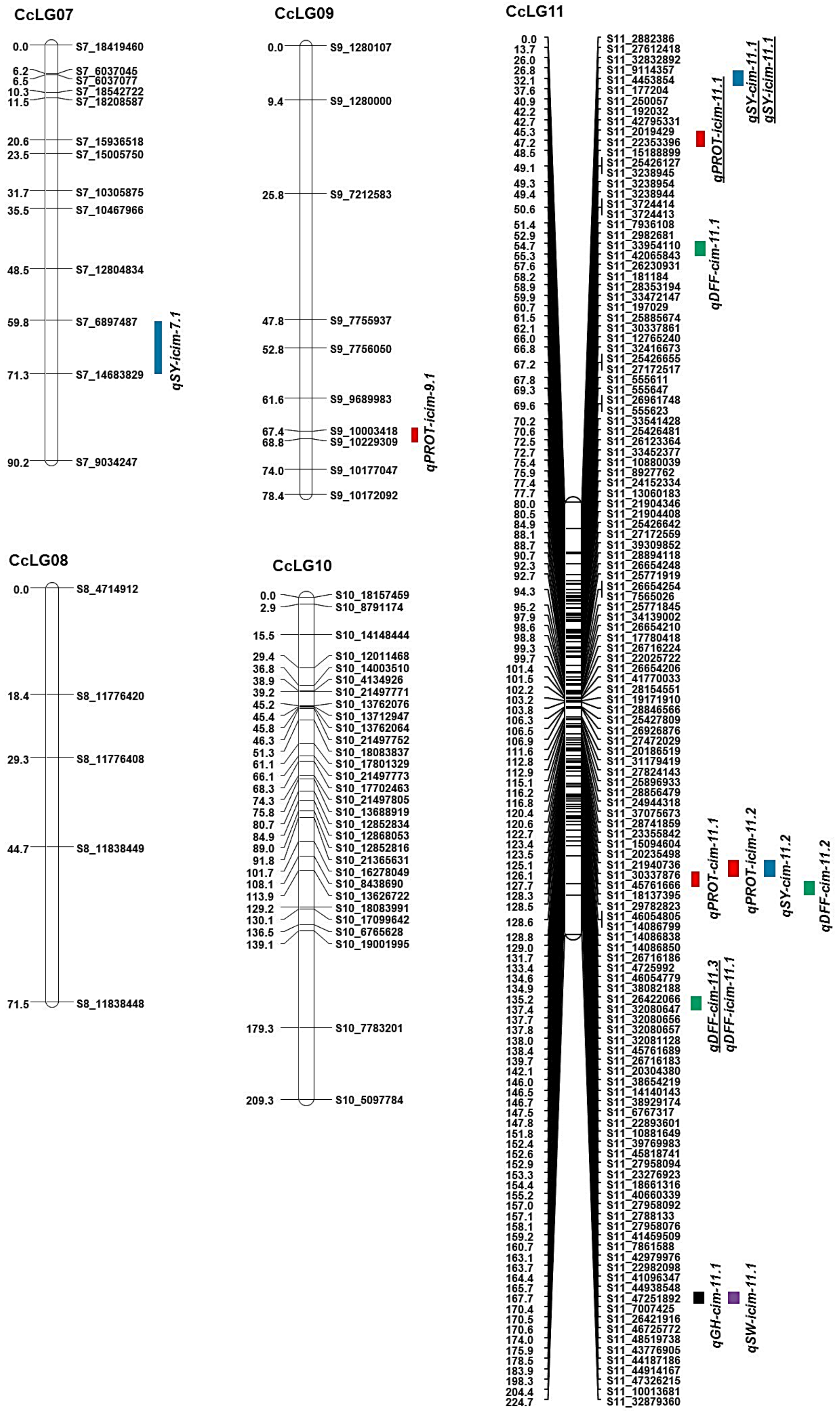

Supplementary Figure S3. Genetic and QTL maps for Pop3 (HPL 24 × ICP 11605). Markers are shown on right side of the linkage group while map distances are indicated on left side. QTLs for the different traits are indicated by different colored bars with red, purple, blue, black and green showing QTLs for seed protein content, seed weight, seed yield, growth habit and days to first flowering, respectively.

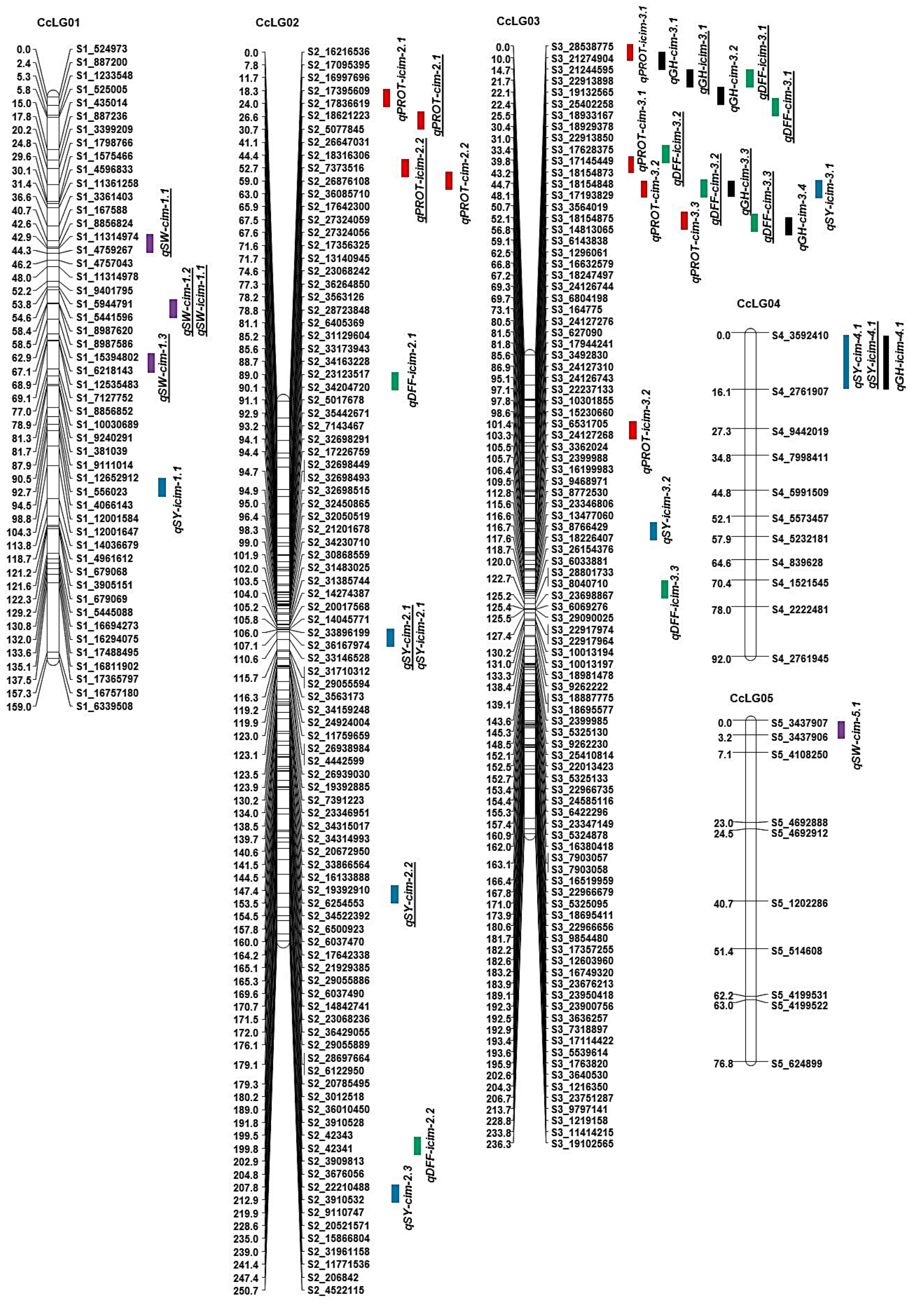

Supplementary Figure S3 (continued)

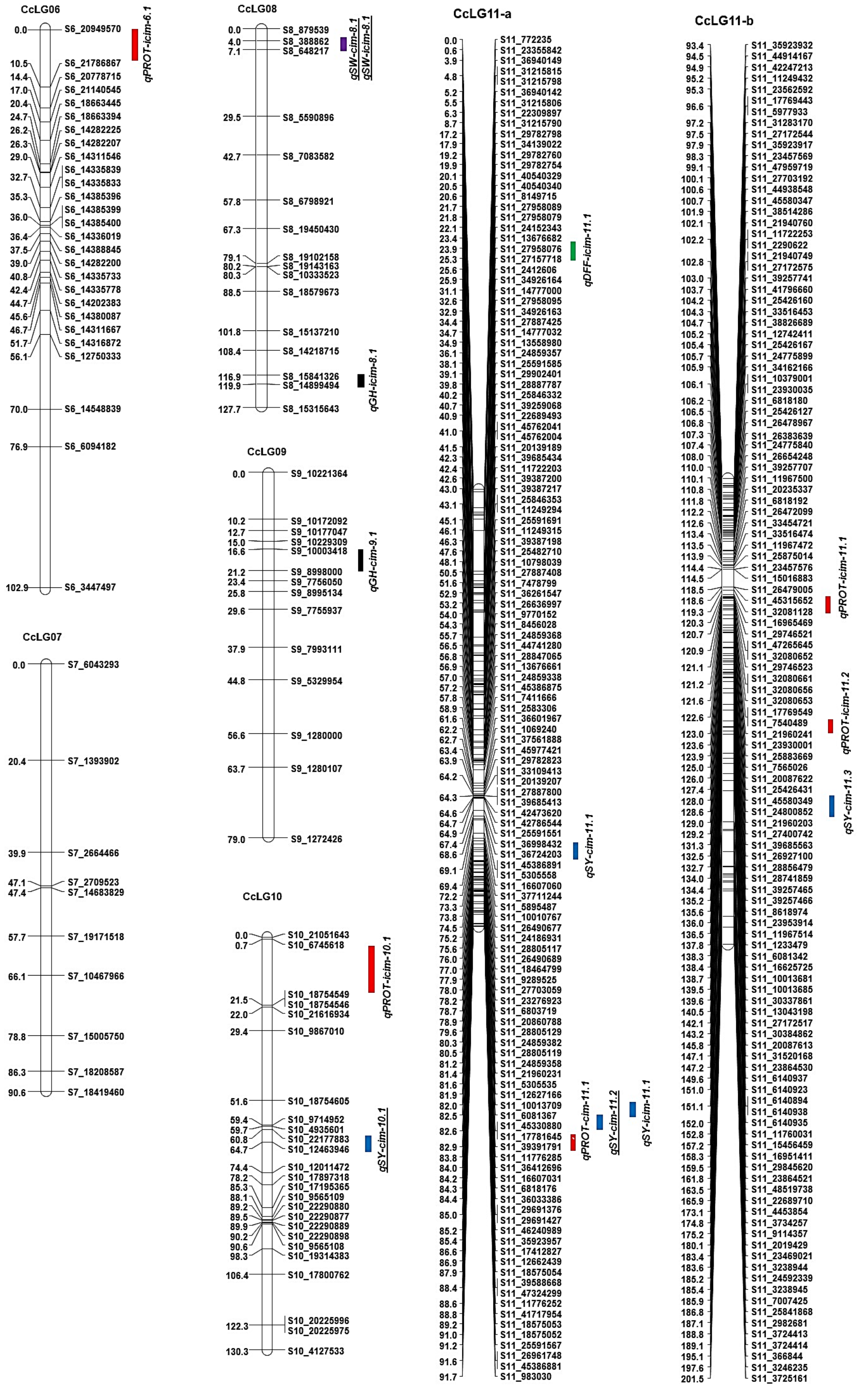

Supplementary Figure S4. Genetic and QTL maps for Pop4 (HPL 24 × ICP 11605). Markers are shown on right side of the linkage group while map distances are indicated on left side. QTLs for the different traits are indicated by different colored bars with red, purple, blue, black and green showing QTLs for seed protein content, seed weight, seed yield, growth habit and days to first flowering, respectively

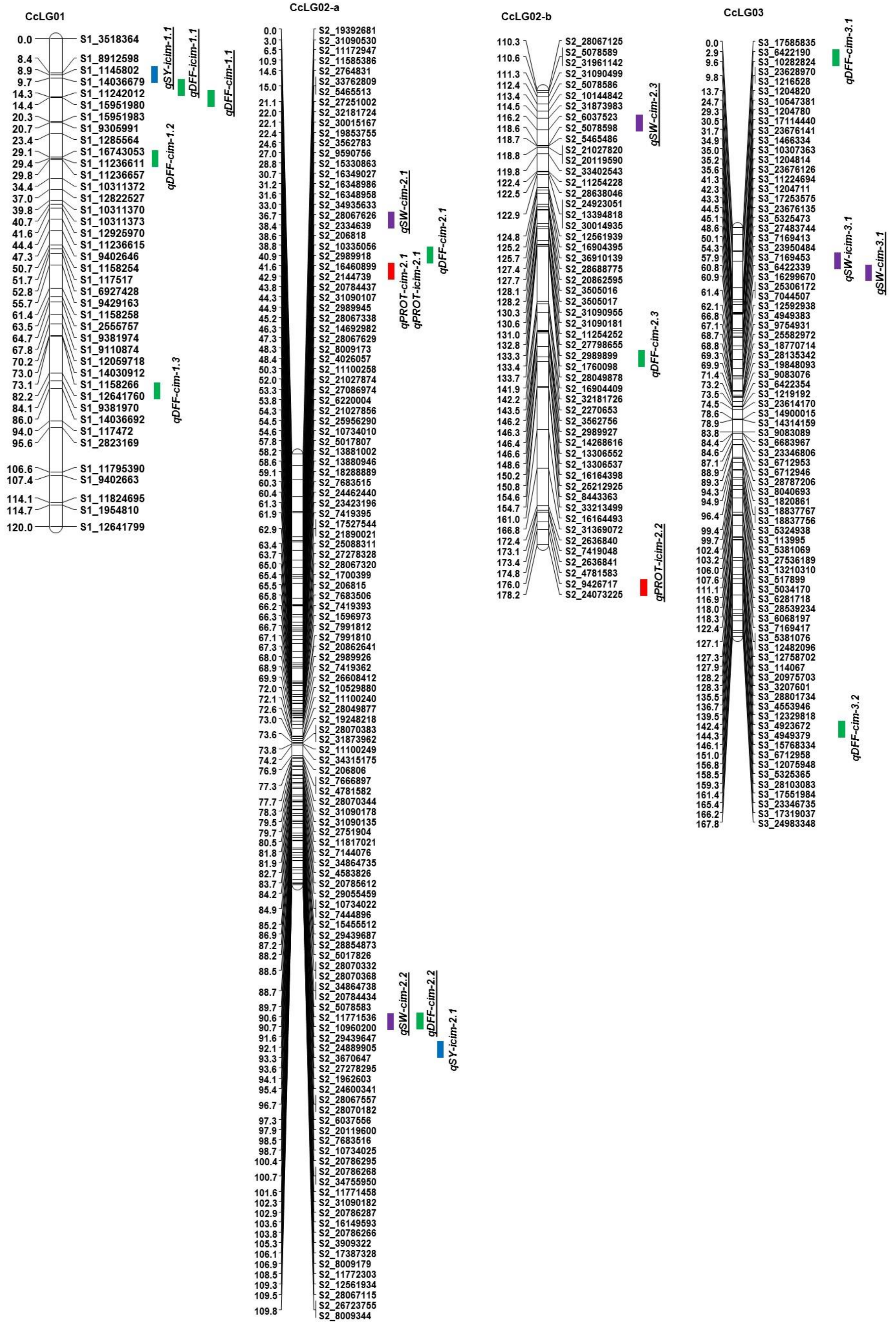

Supplementary Figure S4 (continued)

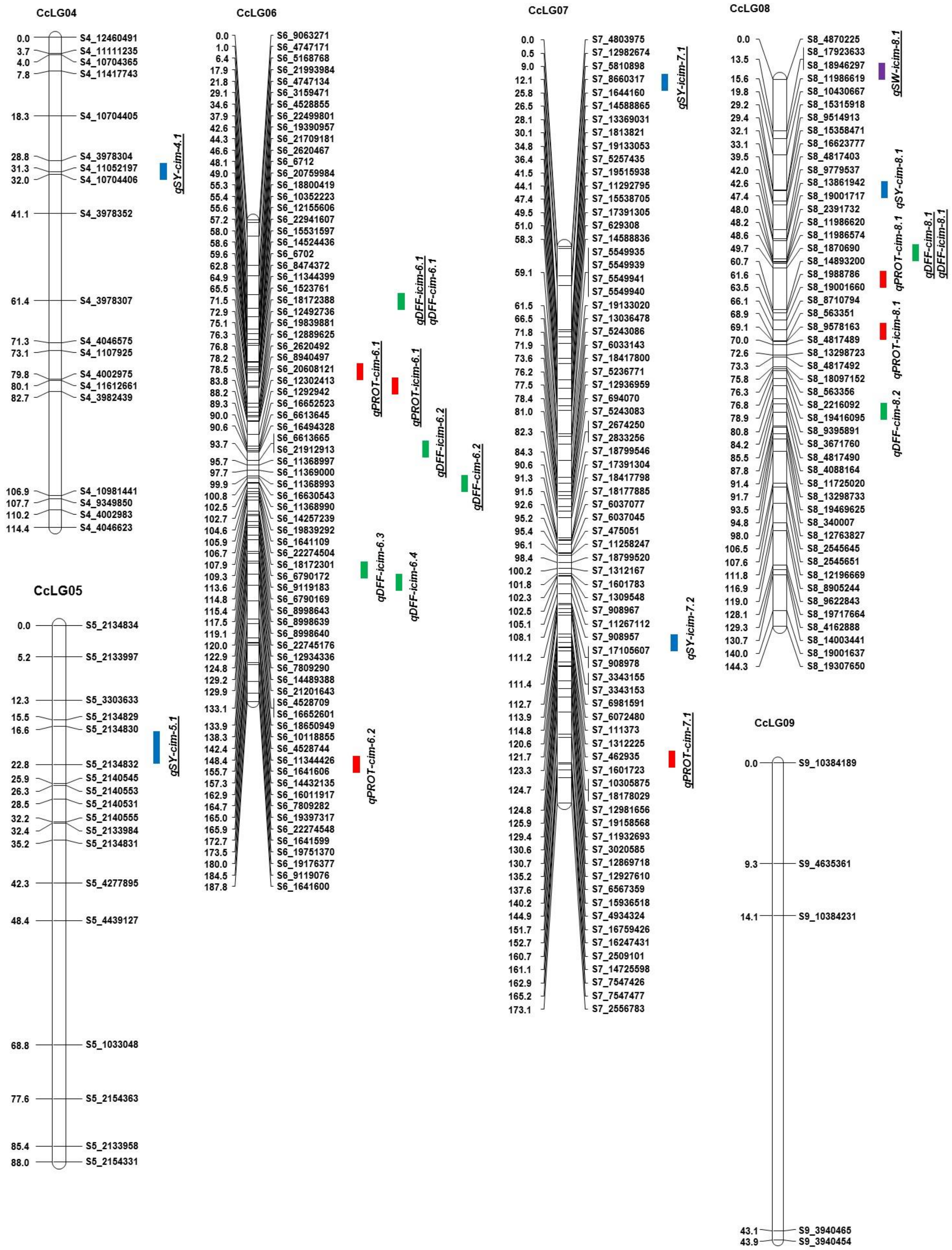

Supplementary Figure S4 (continued)

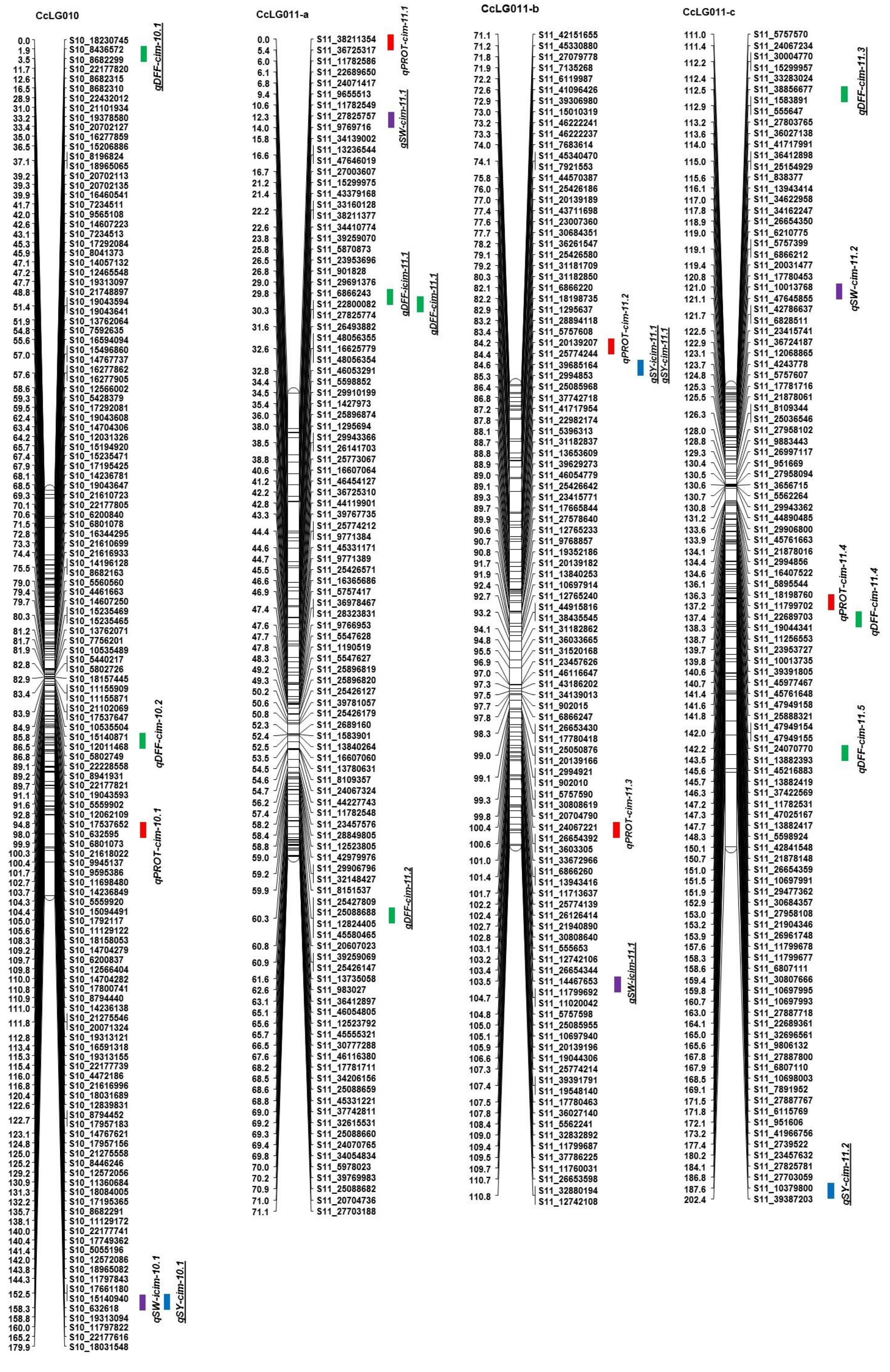

Supplementary Figure S5. Genetic and QTL maps for Pop5 (ICP 5529 × ICP 11605). Markers are shown on right side of the linkage group while map distances are indicated on left side. QTLs for the different traits are indicated by different colored bars with red, purple, blue, black and green showing QTLs for seed protein content, seed weight, seed yield, growth habit and days to first flowering, respectively. Gene names are in parentheses;

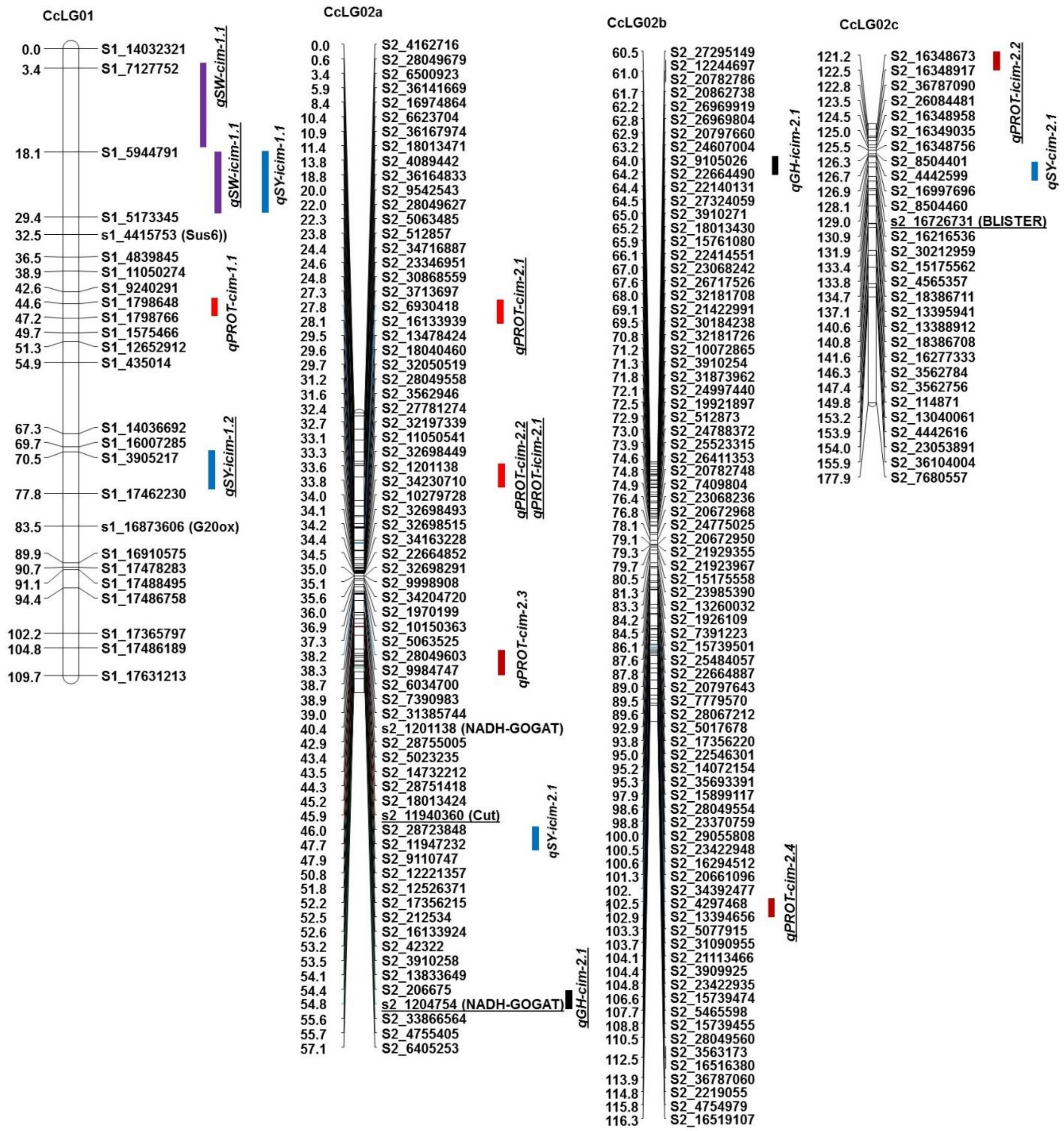

Supplementary Figure S5 (continued)

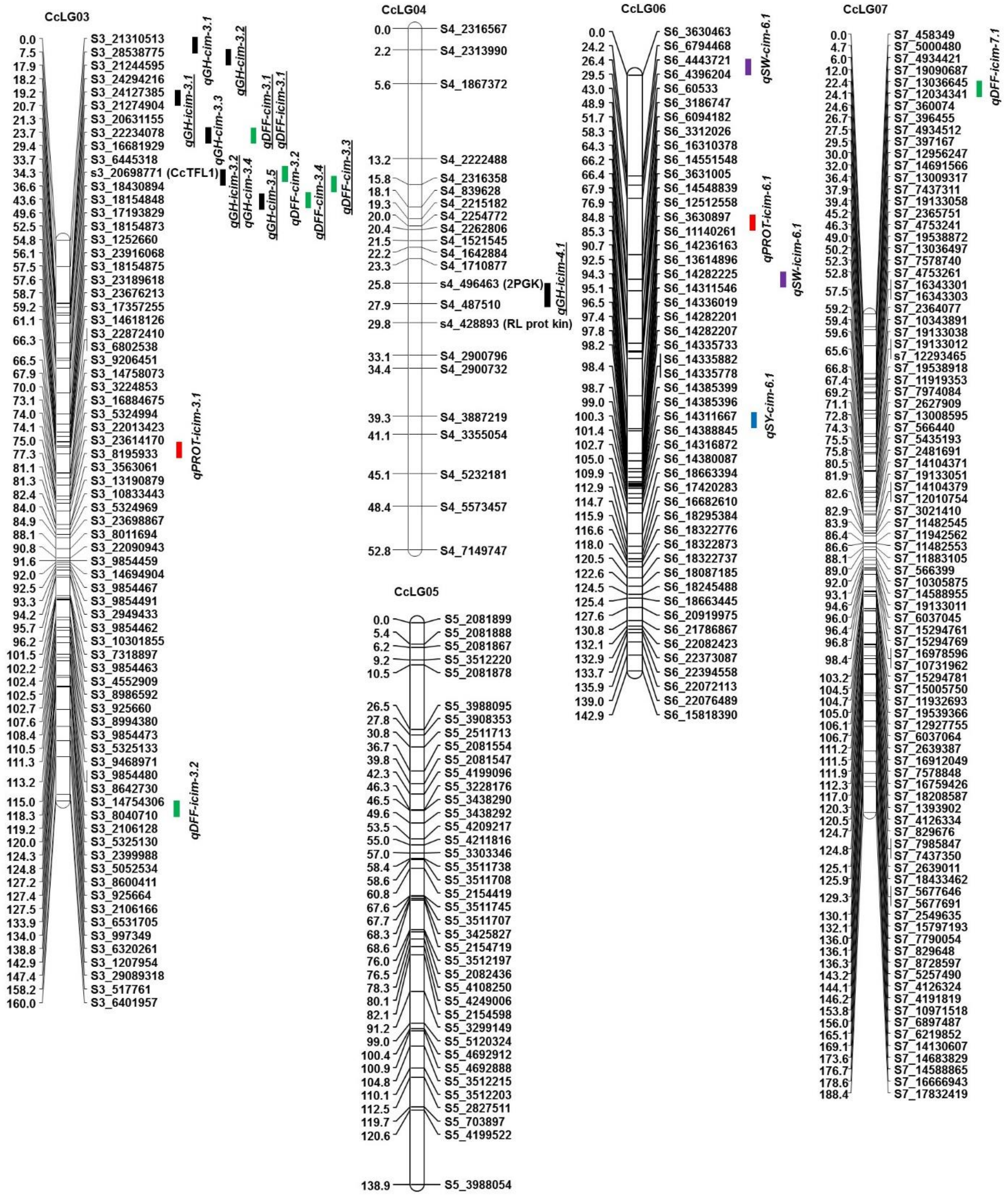

Supplementary Figure S5 (continued)

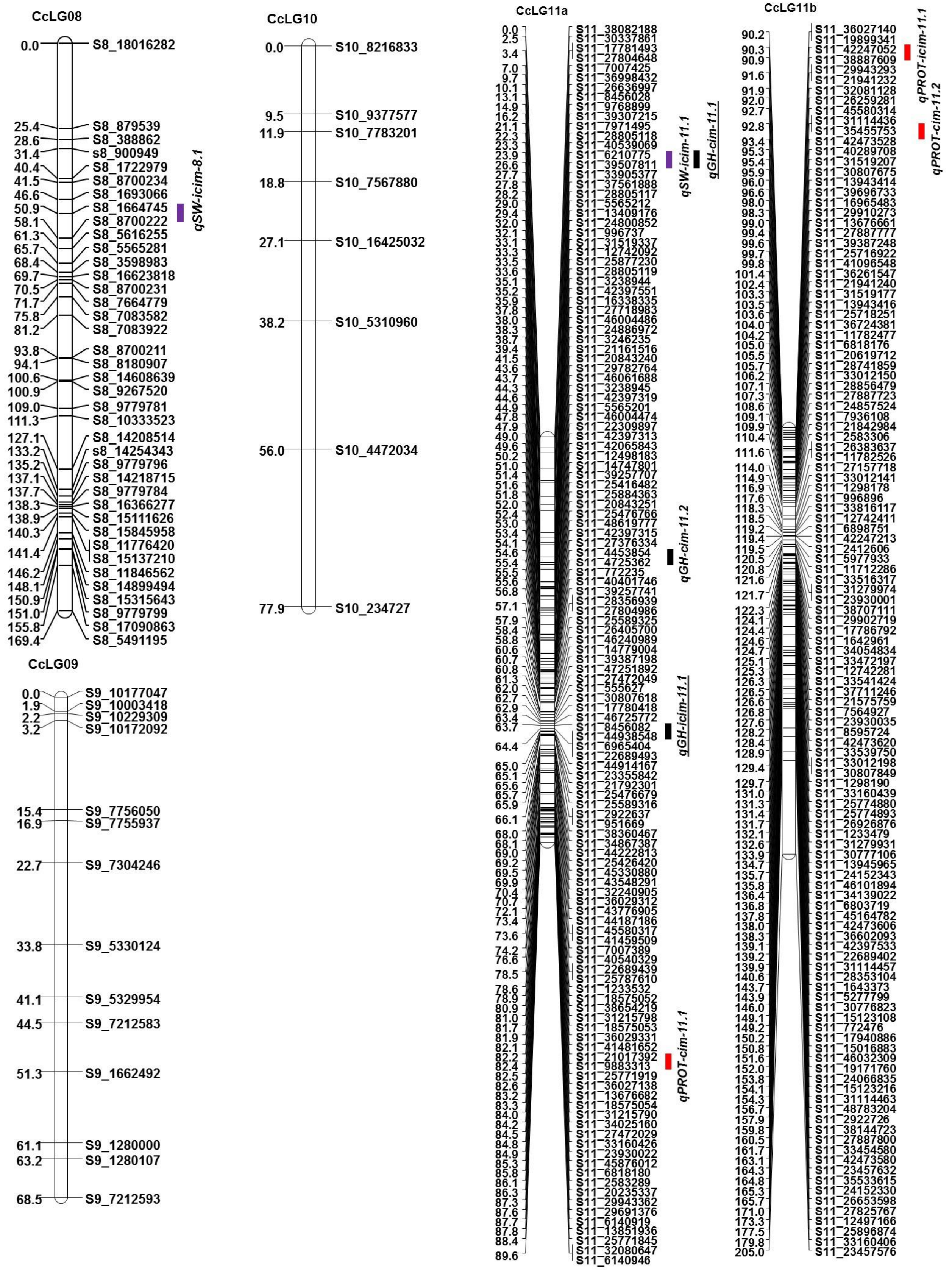

Supplementary Figure S6. Charts depicting marker-based correspondences of consensus with individual genetic maps. a, b, c, d, e, f, g, h, i, j and k are CcLG01, CcLG02, CcLG03, CcLG04, CcLG05, CcLG06, CcLG07, CcLG08, CcL09, CcLG10 and CcLG11, respectively. Only common markers are included to visually asses the collinearity of marker orders and marker positions. Linkage groups were aligned together using comparative mapping programme CMap version 1.01

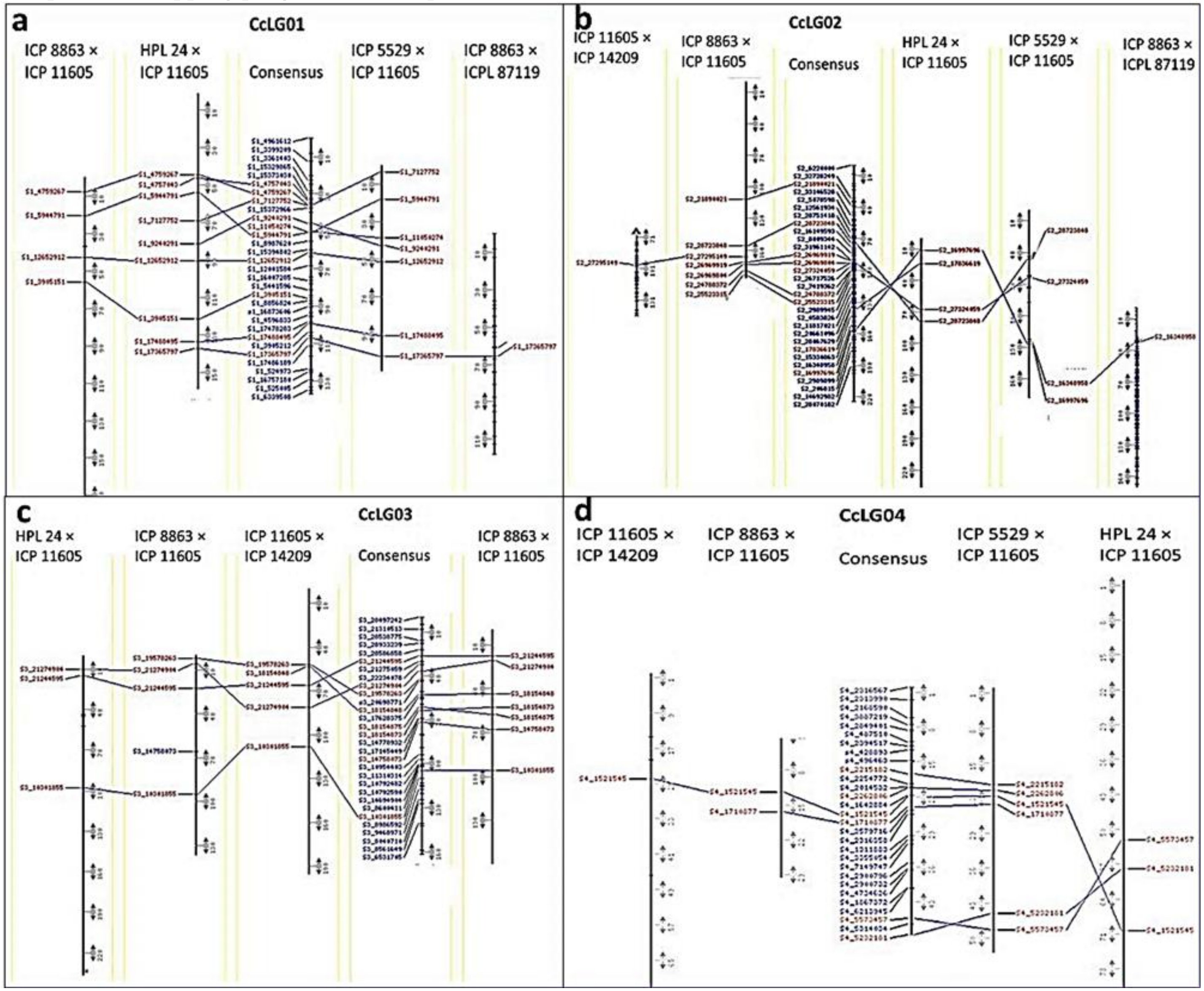

Supplementary Figure S6 (continued)

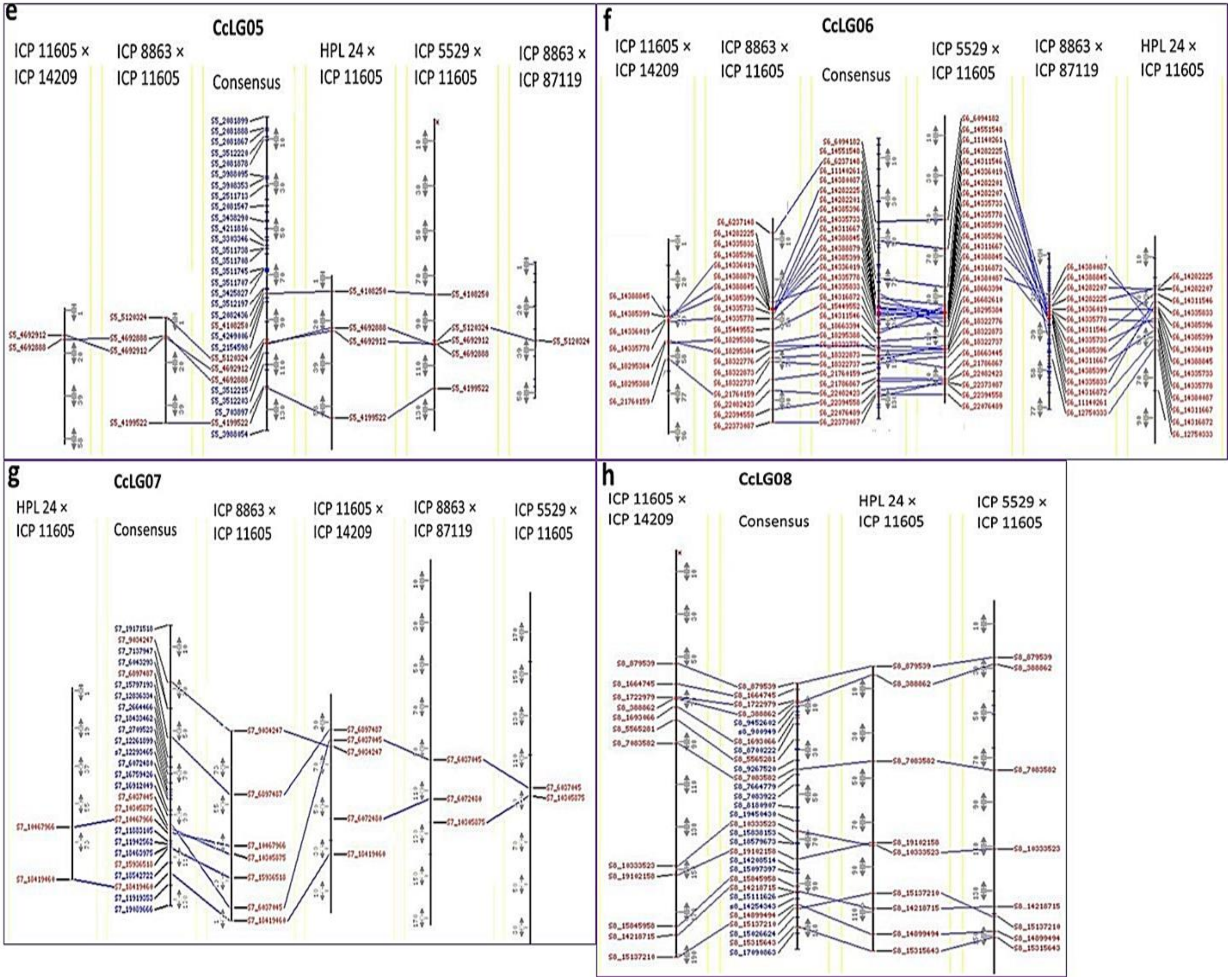

Supplementary Figure S6 (continued)

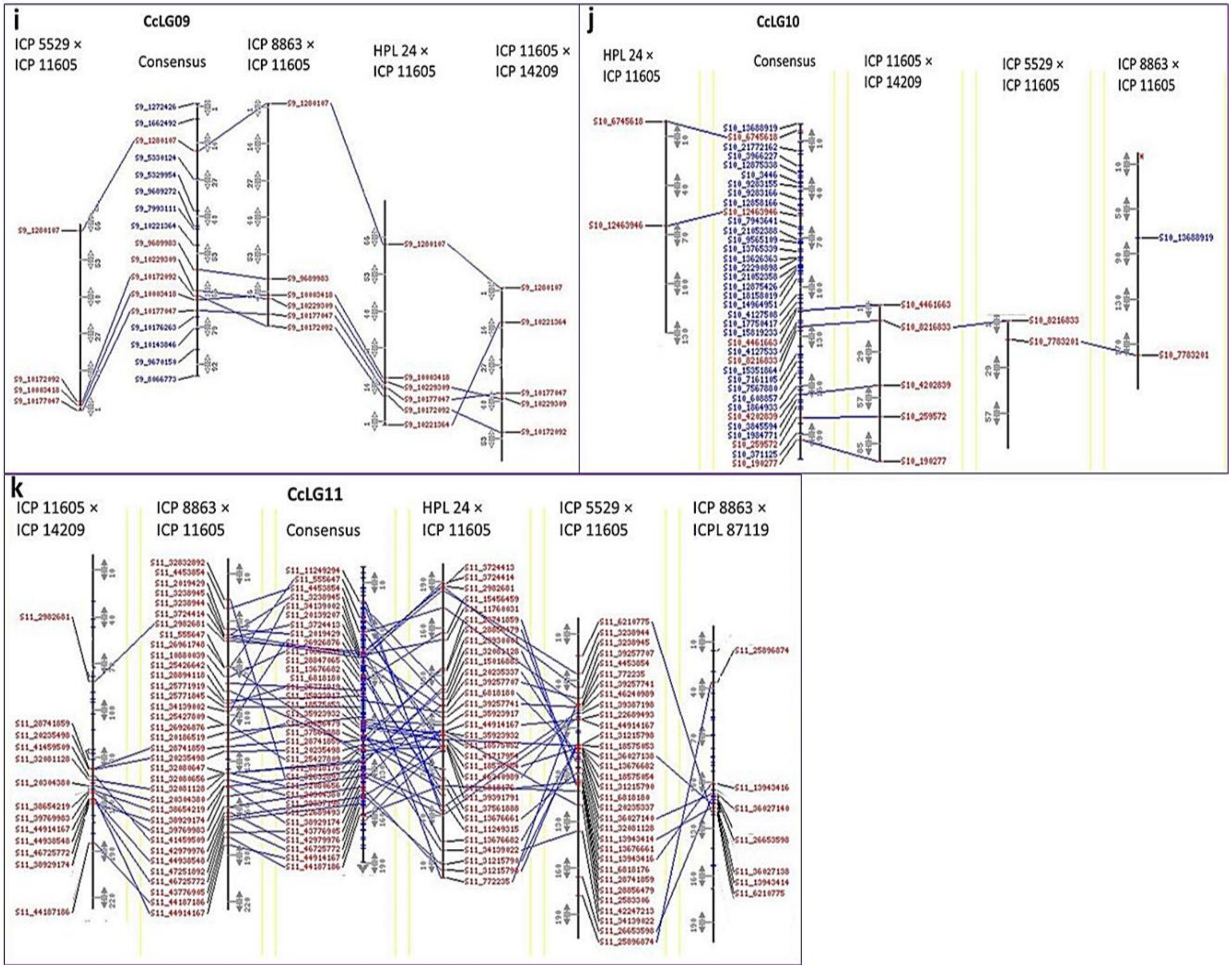

Supplementary Figure S7. Consensus genetic and QTL maps. Markers are shown on right side of the linkage group while map distances are indicated on left side. Markers unique to mapping populations, common between two, three and four mapping populations have been shown by black, red, blue and green colours, respectively. QTLs for the different traits are indicated by colored bars with brown, green, grey, purple and red showing QTLs for GH, SY, DTFF, HSW and SPC, respectively. Source populations of projected QTLs are indicated by dots of different colours where yellow, blue, green, purple and black represent Pop1, Pop2, Pop3, Pop4 and Pop5, respectively. The position of the dot on the QTL bar indicates whether both or only one of the flanking markers were projected on to the consensus map. Where the dot lies at the center of the QTL bar it indicates that both flanking markers of the QTL are present, while if the dot lies either on the upper or lower part of the QTL bar it shows that only one marker closest to the dot was projected.

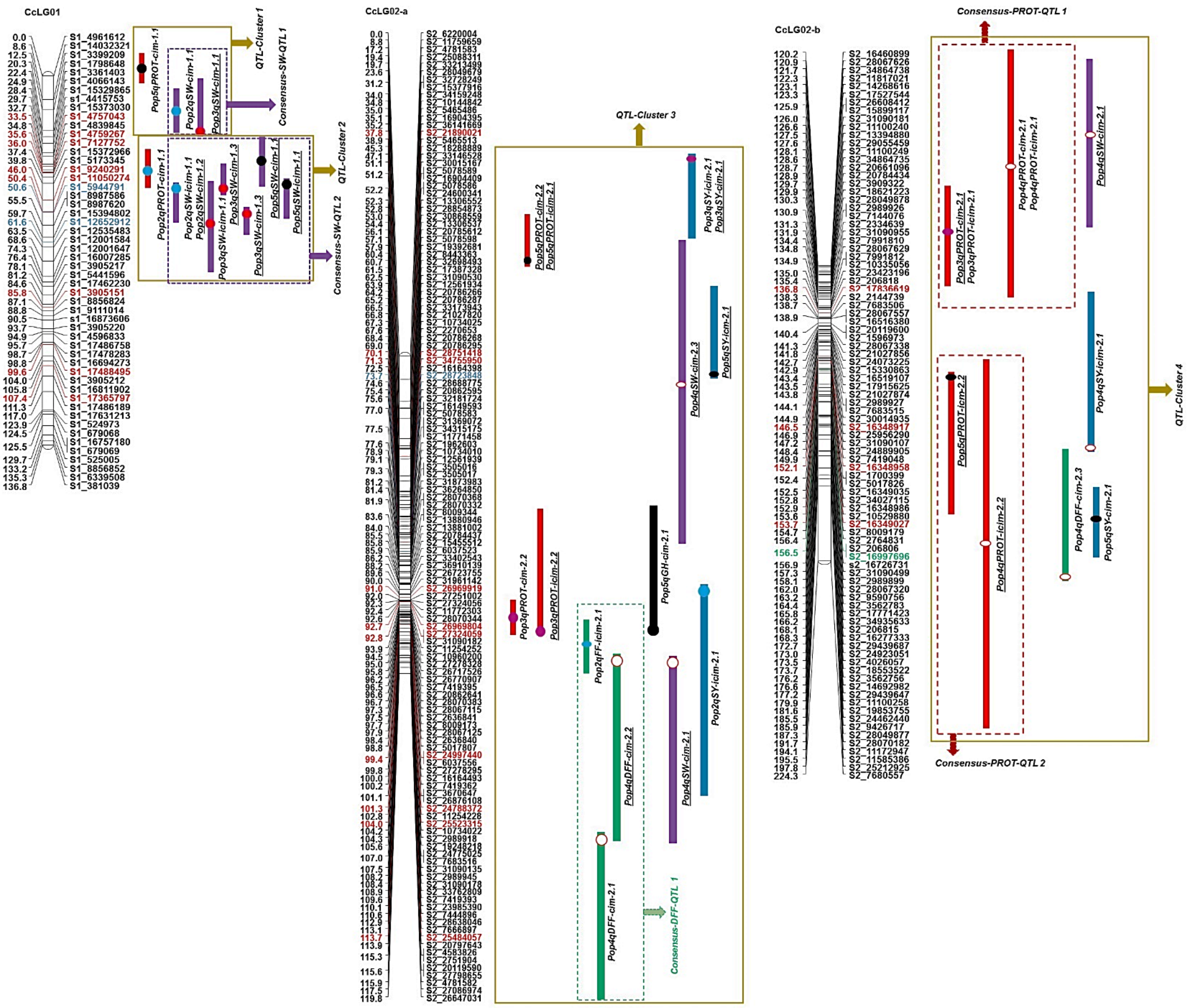

Supplementary Figure S7 (continued)

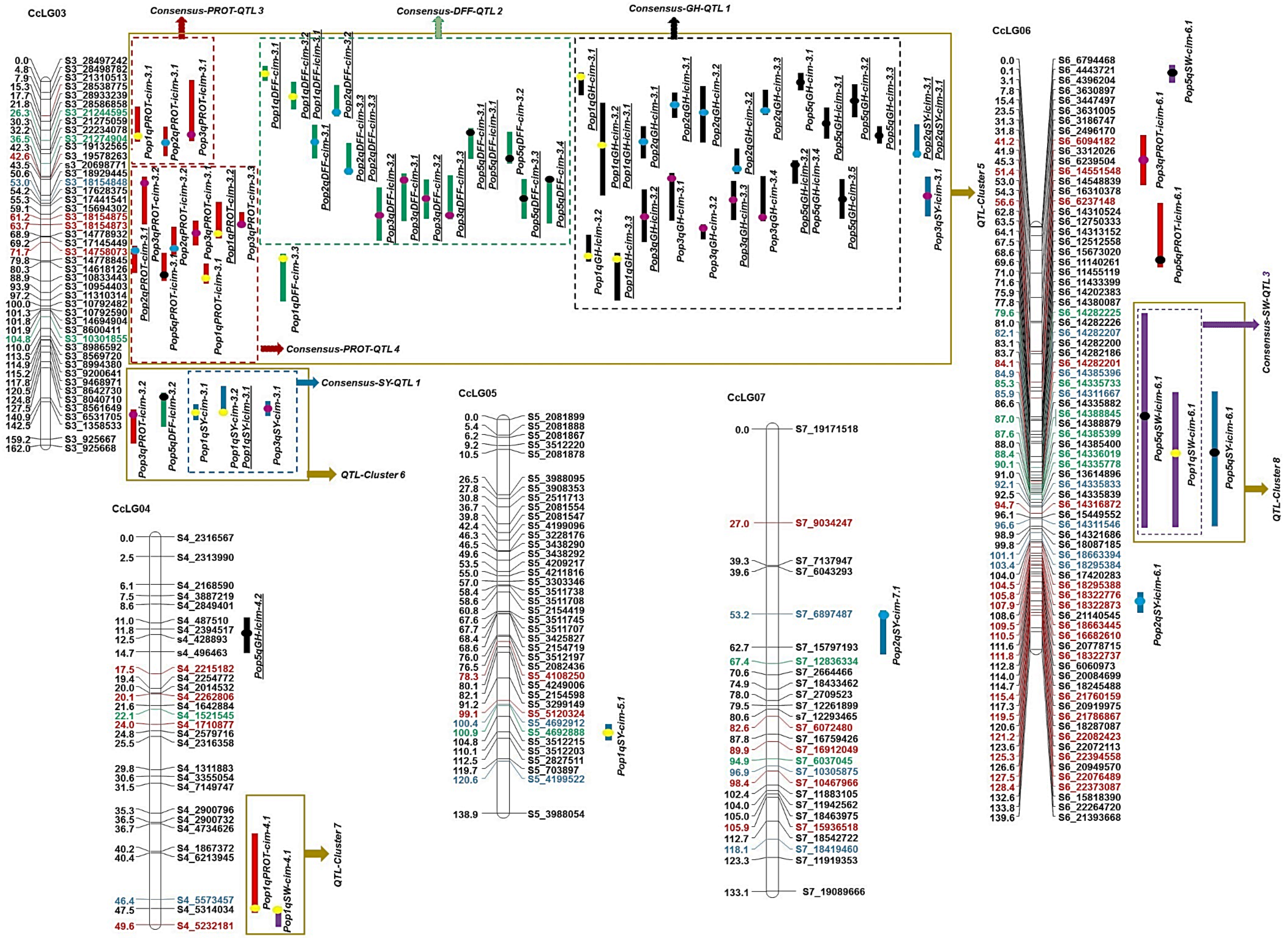

Supplementary Figure S7 (continued)

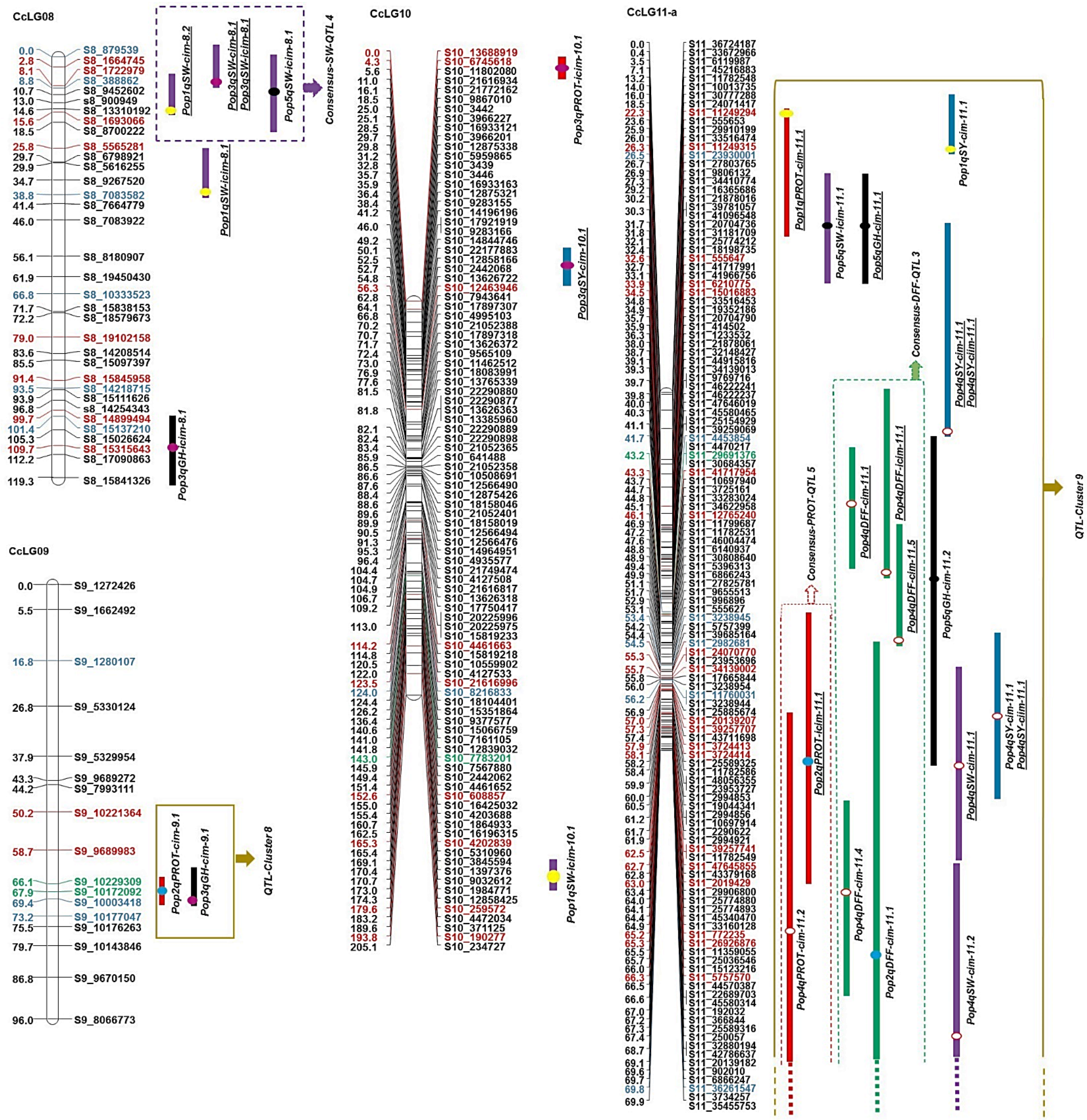

Supplementary Figure S7 (continued)

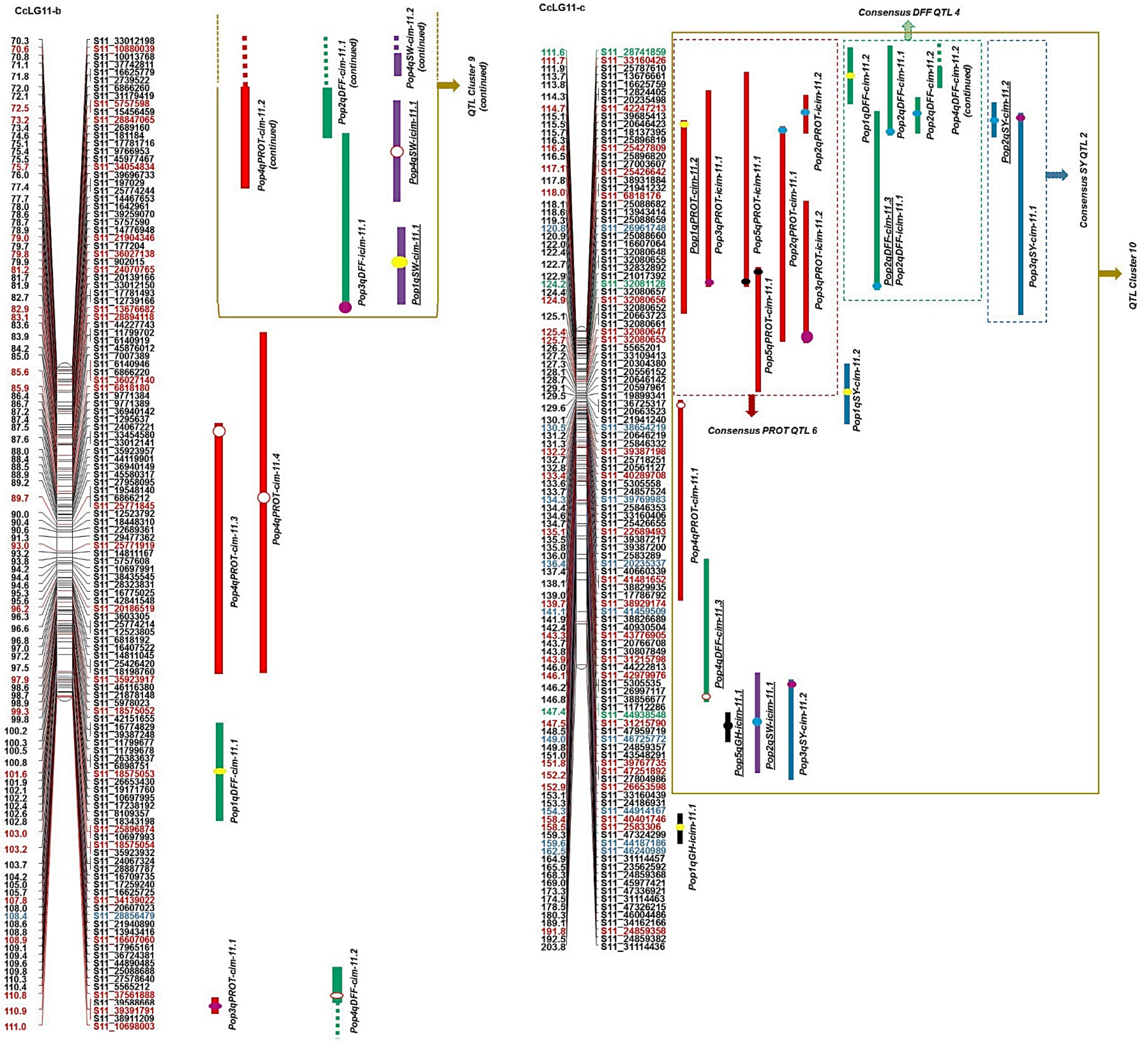

Supplement: Supplementary file 1 — Supplementary Information. [file 41598_2019_56903_MOESM1_ESM.pdf]
